# Quantitative Mass Spectrometry Analysis of PD-L1 Protein Expression, *N*-glycosylation and Expression Stoichiometry with PD-1 and PD-L2 in Human Melanoma*[Fn FN2]

**DOI:** 10.1074/mcp.RA117.000037

**Published:** 2017-05-25

**Authors:** Carlos A. Morales-Betanzos, Hyoungjoo Lee, Paula I. Gonzalez Ericsson, Justin M. Balko, Douglas B. Johnson, Lisa J. Zimmerman, Daniel C. Liebler

**Affiliations:** From the ‡Department of Biochemistry, Vanderbilt University School of Medicine, Nashville, Tennessee;; §Hematology/Oncology Division, Department of Medicine, Vanderbilt University School of Medicine, Nashville, Tennessee 37232

## Abstract

Quantitative assessment of key proteins that control the tumor-immune interface is one of the most formidable analytical challenges in immunotherapeutics. We developed a targeted MS platform to quantify programmed cell death-1 (PD-1), programmed cell death 1 ligand 1 (PD-L1), and programmed cell death 1 ligand 2 (PD-L2) at fmol/microgram protein levels in formalin fixed, paraffin-embedded sections from 22 human melanomas. PD-L1 abundance ranged 50-fold, from ∼0.03 to 1.5 fmol/microgram protein and the parallel reaction monitoring (PRM) data were largely concordant with total PD-L1-positive cell content, as analyzed by immunohistochemistry (IHC) with the E1L3N antibody. PD-1 was measured at levels up to 20-fold lower than PD-L1, but the abundances were not significantly correlated (r^2^ = 0.062, *p* = 0.264). PD-1 abundance was weakly correlated (r^2^ = 0.3057, *p* = 0.009) with the fraction of lymphocytes and histiocytes in sections. PD-L2 was measured from 0.03 to 1.90 fmol/microgram protein and the ratio of PD-L2 to PD-L1 abundance ranged from 0.03 to 2.58. In 10 samples, PD-L2 was present at more than half the level of PD-L1, which suggests that PD-L2, a higher affinity PD-1 ligand, is sufficiently abundant to contribute to T-cell downregulation. We also identified five branched mannose and N-acetylglucosamine glycans at PD-L1 position N192 in all 22 samples. Extent of PD-L1 glycan modification varied by ∼10-fold and the melanoma with the highest PD-L1 protein abundance and most abundant glycan modification yielded a very low PD-L1 IHC estimate, thus suggesting that N-glycosylation may affect IHC measurement and PD-L1 function. Additional PRM analyses quantified immune checkpoint/co-regulator proteins LAG3, IDO1, TIM-3, VISTA, and CD40, which all displayed distinct expression independent of PD-1, PD-L1, and PD-L2. Targeted MS can provide a next-generation analysis platform to advance cancer immuno-therapeutic research and diagnostics.

Immune checkpoint proteins, such as programmed cell death 1 (PD-1; PDCD1) and its ligand programmed cell death 1 ligand 1 (PD-L1^1^, CD274) mediate inhibition of CD8+ effector T cells (1). In the microenvironment of many tumors, binding of T-cell PD-1 to PD-L1 on tumor cells, lymphocytes or macrophages inactivates CD8+ T cells, thereby protecting tumors from destruction by the immune system (2). Inhibition of the PD-1/PD-L1 interaction with therapeutic antibodies, such as pembrolizumab, nivolumab, or atezolizumab has emerged as an effective treatment for several cancers, including melanoma (3–5), renal clear cell carcinoma (6), nonsmall cell lung cancer (7–9), urothelial carcinoma (10, 11), and head and neck carcinoma (12). Durable responses to immune checkpoint therapies in typically lethal cancers have generated widespread, intense interest in immunotherapeutics (13).

One of the most critical questions in immuno-oncology is which patients will benefit from immune checkpoint therapeutics. Objective responses are seen in only 15–45% of patients with solid tumors treated with immune checkpoint drugs (14), which indicates both incomplete mechanistic understanding of the tumor-immune interface and a paucity of biomarkers for critical features. The principal biomarker used to predict therapeutic response to immune checkpoint therapeutics is the PD-L1 protein, which is measured by immunohistochemistry (IHC) (15). PD-L1 IHC tests are approved as a companion diagnostic for pembrolizumab and atezolizumab. However, PD-L1 IHC is an unreliable predictor of individual therapeutic responses. Across several reported clinical trials, up to half of PD-L1 IHC-positive tumors failed to respond to therapy, whereas ∼15% of PD-L1 IHC-negative tumors did respond (16). Clinical application of PD-L1 IHC is further complicated by the availability of four commercially available tests, which employ different antibodies and different cutoffs for assessment (15). Several other factors also influence responses to immune checkpoint therapeutics, including tumor neoantigen load and expression (17–19), extent of T-cell infiltration and composition of T-cell subsets (20, 21), and activities of other immune checkpoint proteins, costimulators and inhibitory molecules (14, 22–24).

Although PD-L1 is the most extensively studied PD-1 ligand, programmed cell death 1 ligand 2 (PD-L2, PDCD1LG2) also can inactivate T cells by binding to PD-1 (25). PD-L2 binds to PD-1 with ∼2–6 fold higher affinity than does PD-1, as estimated by flow cytometry and surface plasmon resonance (26). Interest in PD-L2 has been tempered by the apparently low expression of the protein in solid tumors (27), which is reportedly restricted to myeloid cells (14). However, Danilova *et al.* recently noted limitations of available PD-L2 antibodies for IHC and used mRNA transcript data to compare the expression of PD-L2 with PD-L1 and PD-1 (28). Measurements of PD-1, PD-L1 and PD-L2 also may be affected by glycosylation, which has been reported for PD-L1 in breast cancer cells (29) and by proteolysis of PD-L1 and PD-L2 by matrix metalloproteinases (30).

Given the well-documented limitations of antibody-based immune checkpoint measurements, we considered measurements based instead on targeted MS by parallel reaction monitoring (PRM), which has emerged as a sensitive, highly specific and robust platform for the quantitative analysis of proteins (31, 32). PRM analysis with stable isotope labeled internal standards (stable isotope dilution, SID) yields molar quantitation, which enables direct comparison of protein stoichiometry in functional cellular systems (33). Moreover, targeted MS is ideal for the analysis of clinical specimens, including formalin-fixed, paraffin-embedded (FFPE) sections (34). PRM provides a platform to systematically configure multiplexed, targeted assays for simultaneous analysis of dozens of proteins, thereby enabling focused quantitative analysis of biological systems and pathways (35, 36).

Here we describe the development of an addressable fractionation-PRM platform, which provides measurements of PD-1, PD-L1, and PD-L2 at femtomole per microgram tissue protein levels in FFPE sections of human melanoma biopsies. We compared our analyses to IHC measurements of PD-L1 in adjacent sections and further characterized the effects of variable cellular composition on measured PD-1, PD-L1, and PD-L2. Our measurements demonstrate substantial between-tumor variation in expression ratios of these proteins and suggest that PD-L2 is present in some tumors at levels sufficient to contribute to PD-1-dependent T-cell regulation and possibly to affect responses to PD-1- and PD-L1-blocking drugs. We further report the identification and quantitation of several N-glycosylated forms of PD-L1 in the same samples and provide evidence that post-translational modifications of PD-L1 may affect PD-L1 detection by IHC. Finally, we demonstrate the extension of our addressable fractionation-PRM platform to analyze several other immune checkpoint and coregulator proteins, which are targets for new drug development or which affect responses to immunotherapeutics. The data demonstrate the potential utility of targeted MS as a next-generation analysis platform to advance cancer immuno-therapeutic research and diagnostics.

## MATERIALS AND METHODS

### 

#### 

##### Materials and Reagents

Recombinant human PD-L1 protein (containing a C-terminal lG1-Fc tag) and PNGase-F were purchased from Thermo Fisher Scientific (Waltham, MA) and Promega (Madison, WI), respectively. Sep-Pak C18 desalting cartridges (1cc, 100 mg) and XBridge C18 (4.6 × 250 mm, 5 μm) columns were from Waters (Milford, MA). ReproSil C18-AQ resin (3 μm particle size) was purchased from Dr. Maisch, Gmbh (Ammerbuch-Entringen, Germany). Picofrit self-pack columns (75 μm ID, 10 μm ID tip) were from New Objective (Woburn, MA). An equimolar predigested bovine 6 protein mix used as an MS system performance standard was purchased from Bruker-Michrom, Inc. (Auburn, CA). Trypsin (Trypsin Gold) was from Promega (Madison, WI). C-terminal isotopically labeled peptides containing U-^13^C_6_, U-^15^N_4_-arginine, or U-^13^C_6_, U-^15^N_2_-lysine and unlabeled peptide standards were purchased from New England Peptide (Gardner, MA). Isotope labeled peptides were of greater than 99 and 95% isotopic and chemical purity, respectively; absolute concentration was determined by amino acid analysis.

##### Human Melanoma Biopsy Specimens

Tissue sections (5 μm thickness) were obtained from archival FFPE tumor blocks from patients treated for melanoma at Vanderbilt University Medical Center. All participants provided informed consent for use of tissue specimens and the study was approved by the Vanderbilt Institutional Review Board (IRB Approval numbers 100178 and 030220). Participants all received immune checkpoint inhibitors as second or third line therapy. Individual demographic information, therapies and responses, biopsy site(s) sampled and other relevant information are provided in supplemental Table S1. Samples were fixed in 10% buffered neutral formalin, processed and paraffin-embedded by standard methods in automated tissue processor. Five-micrometer sections were subjected to hematoxylin and eosin (H&E) staining and dual IHC using antibodies against PD-L1 (clone E1L3N, Cell Signaling Technology, Danvers, MA) and SOX10 (clone BC34, Biocare Medical, Pacheco, CA). Sections analyzed by hematoxylin and eosin (H&E) staining and IHC were serially adjacent to those analyzed by MS.

##### IHC Analysis of PD-L1

Tissue was incubated overnight at 4 °C with both rabbit monoclonal antibody against PD-L1 (clone E1L3N, Cell Signaling Technology, Catalogue# 13684, 1:100 dilution) and mouse monoclonal antibody against SOX-10 (clone BC34, Biocare, dilution 1:200). (SOX10 analyses were part of a separate studies and are not discussed here further.) Antigen retrieval was performed using Citrate Buffer pH6 (Biocare Decloaking Chamber). Visualization was preformed using MACH2 system (Biocare), DAB (SOX10) and Fast Red (PD-L1) as chromogens, and counterstained with hematoxylin. The slides were scanned using a high-resolution scanner (Leica SCN400 Slide Scanner) at 20× magnification. PD-L1 was scored and expressed as a percentage of stained cells for tumor cells, peri- and intratumoral immune infiltrate (mostly mononuclear phagocytic cells), and other nontumor tissue cells.

##### Cell Culture and Preparation of Lysates

HEK-293 cells (CRL-1573, ATCC) were grown in DMEM (Life Technologies, Carlsbad, CA) supplemented with 10% fetal bovine serum (Atlas Biologicals, Fort Collins, CO) at 37 °C in a humidified atmosphere with 5% CO_2_. Cells were harvested, washed twice in phosphate-buffered saline, and frozen at 80 °C until used. Frozen cell pellets were lysed on ice in 8 m urea supplemented with 1× HALT protease and phosphatase inhibitor (Life Technologies). Lysates were clarified by centrifugation at 10,000 × *g* for 10 min at 4 °C and desalted with a Sep-Pak C18 1cc Vac Cartridge (100 mg, 55–105 μm particle size (Waters, Milford, MA). Protein in the lysates was measured with the bicinchoninic acid (BCA) assay (Pierce, Rockford, IL).

##### Extraction and Digestion of Proteins from FFPE Melanoma Sections

The material from two 5 μm FFPE tissue sections was scraped from the glass slides using a clean razor blade and transferred into a clean 1.5 ml Eppendorf tube. FFPE tissue was deparaffinized and hydrated as previously described (34) with the following modifications. After hydration, the proteins were re-suspended in 100 μl of 100 mm ammonium bicarbonate, pH 8.0 (AmBic). Proteins were extracted from the rehydrated specimens by a two-step procedure. First, 100 μl of trifluoroethanol was added to each sample, followed by the addition of 100 μl of AmBic. Each tube was sonicated 3 times continuously for 20 s using a Sonic Dismembrator probe at level 2 (Model 100, Fisher Scientific, Waltham, MA), with cooling of samples on ice for 30 s between each sonication step. Samples then were gently shaken for 60 min at 60 °C. For the second extraction step, 100 μl of 10 m urea was added to each tube and sonication steps, described above, were repeated. Samples then were gently shaken for 60 min at 60 °C. Protein concentration in lysates was measured using the BCA assay (ThermoFisher Scientific, Waltham, MA).

For protein digestion, aliquots of lysate corresponding to 100 μg protein were diluted to a volume 200 μl with AmBic. A 50-mM solution of dithiothreitol, prepared in 50 mm Ambic, was added to a final concentration of 5 mm and solutions were incubated for 30 min at 60 °C. Iodoacetamide (100 mm prepared in HPLC-grade water) was added to a concentration of 10 mm and the solution was incubated in the dark for 20 min at room temperature. Samples were diluted further with 800 μl of AmBic to achieve final concentrations of ≤10% trifluoroethanol and ≤ 1 m urea before digestion. Trypsin (2 μg) was added to each sample to achieve a 1:50 (w/w) trypsin/protein ratio and the samples were incubated overnight at 37 °C. After 16 h digestion, the samples were frozen at −80 °C and evaporated to dryness under vacuum.

##### Basic Reverse Phase Liquid Chromatography (bRPLC) Peptide Fractionation

Dried sample residues were re-suspended in 350 μl deionized water and desalted with an Oasis HLB μElution Plate (30 μm particle size, Waters). Plates were prewashed with 500 μl of acetonitrile and equilibrated with 750 μl of HPLC-grade water. The flow-through was discarded and the plates were washed with 500 μl of HPLC-grade water and the peptides were eluted with 80% acetonitrile. A mixture of 50 fmol each of the isotopically labeled peptide SID and LRP standards (see below) was added to each eluted peptide mixture. The tryptic peptides from the 100 μg digests then were fractionated using bRPLC separation with an Agilent 1260 Infinity HPLC System with a XBridge C18, 250 mm × 4.6 mm analytical column (130A, 5 μm particle size) and a XBridge C18 Sentry guard cartridge at a flow rate of 0.5 ml/min. The mobile phase consisted of 10 mm triethylamine bicarbonate pH 7.5 (TEAB) in water as solvent A and 10 mm TEAB in 90% acetonitrile as solvent B. The mobile phase was programmed from zero to 5% B in 5 min, from 5% to 35% B in 45 min and then held at 90% B for 10 min before returning to initial conditions. A total of 12 fractions were collected over time slices of 4.75 min each for the first 50 min of the program. The fractions were evaporated to dryness under vacuum and stored at −80 °C until PRM analysis.

##### Parallel Reaction Monitoring (PRM) Targeted MS

PRM assays were performed on a Q Exactive Plus mass spectrometer (ThermoFisher Scientific, San Jose, CA) equipped with a Easy nLC1000 LC and autosampler system (ThermoFisher Scientific). For each analysis, 5 μl of each sample was injected onto a PicoFrit capillary column (New Objective, 30 cm × 75 μm) packed ReproSil-Pur C18 AQ 3 μm resin (Dr. Maisch GmbH). The column was heated to 45 °C. Solvent A was 0.1% formic acid in water and solvent B was 0.1% formic acid in acetonitrile. Peptides were separated at a flow rate of 400 nL/min using a linear gradient of 2% solvent B for 1 min followed by increase to 28% solvent B over 48 min, then to 60% solvent B over 5 min, followed by an increase to 90% solvent B. The mobile phase was then held at that composition for 7 min before returning to initial conditions.

The acquisition method consisted of a full scan selected ion monitoring event followed by 14 targeted MS2 scans as triggered by a scheduled inclusion list, with a 5-min retention time window containing the precursor *m*/*z* values. Retention times were determined from prior analyses of synthetic peptide standards. The MS1 scan was collected at a resolution of 17,500, an automatic gain control (AGC) value of 3e6, a max injection time of 64 msec, and a scan range from *m*/*z* 380–1500. MS1 data were recorded in profile mode. The MS1 scan was followed by 14 targeted MS2 scans at a resolution of 70,000, an AGC value of 1e6, a max injection time of 240 msec, 0.7 *m–z* isolation window, fixed first mass of 200 *m–z*, an optimized collision energy for each target of 20, 23, or 27%. MS2 data were recorded in profile mode. Lists of all peptides targeted in the PRM analyses are provided in supplemental Table S2.

##### Stable Isotope Dilution (SID) analysis of PD-1, PD-L1, and PD-L2 Peptides

Information on peptides and labeled standards representing PD-1, PD-L1, and PD-L2, including bRPLC fractions, precursor *m*/*z*, charge state, normalized collision energy and PRM transitions extracted is presented in supplemental Table S2. Reverse calibration curves were generated for each peptide pair by spiking a constant level of 2.5 fmol/μl of light peptide and heavy peptide standard at concentrations from a low value of 0.01562 fmol/μl to a high value of 10 fmol/μl in 0.56 μg/μl HEK-293 lysate background. Linearity of calibration curves was assessed and an equation for determining the analyte concentration using the peak area ratio of light/heavy isotopologs was defined using QuaSAR (37), which was implemented through the Skyline interface (38). The lower limit of quantitation (LLOQ) and the lower limit of detection (LLOD) for each peptide also were calculated with QuaSAR. PRM transitions were extracted from raw datafiles and analyzed with Skyline (39). Peptide peak areas were calculated as the sum of three most abundant transitions.

##### Labeled Reference Peptide (LRP) Analysis of IDO1, LAG3, HAVCR2 (TIM-3), C10orf54 (VISTA), and CD40 Peptides

Information on peptides and LRP standards representing IDO1, LAG3, HAVCR2 (TIM-3), C10orf54 (VISTA), and CD40, including bRPLC fractions, precursor *m*/*z*, charge state, normalized collision energy, and PRM transitions extracted is presented in supplemental Table S2. PRM transitions were extracted with Skyline and peak areas for the target peptides were normalized to the peak areas for isotope labeled standard peptides that were contained in the same bRPLC fractions as the target peptides. The ratio of target peptide peak area to LRP peptide peak area was used to compare abundances of the target peptides across the sample set (40).

##### Analysis of PD-L1 N-linked Glycopeptides

Recombinant human PD-L1 was reduced, alkylated, and digested with trypsin as described above. The digest (100 μg) was treated with 1 μl of protein N-glycanase F (PNGase F; Promega, Madison, WI) in 25 μl AmBic, with incubation overnight at 37 °C. The PD-L1 *N*-linked site was identified based on the difference in mass (0.98 Da) from the native sequence associated with conversion of the asparagine glycosylation site to aspartate after PNGase F treatment.

Structures of the intact *N*-linked glycopeptides were initially identified by untargeted MS/MS analyses of the tryptic digest of recombinant human PD-L1. The chromatographic retention of heterogeneous *N*-linked glycopeptides is like that of the corresponding unmodified peptide in reverse phase chromatography, because the primary stationary phase interaction is with the hydrophobic peptide backbone (41, 42). Accordingly, PD-L1 tryptic peptides and their glycopeptides eluted in the same bRPLC fraction and eluted closely in reverse phase LC-MS/MS analyses. Possible glycan structures for N-glycosylated peptides were identified from MS1 data with the GlycoMod tool (http://web.expasy.org/glycomod/). The structures of *N*-linked glycopeptides from both recombinant PD-L1 and from melanoma specimens were manually characterized from MS/MS spectra. Only peak assignments with mass accuracy within 10 ppm of theoretical *m*/*z* were accepted for manual characterization.

##### Statistical Design and Experimental Rationale

Analyses of the recovery of PD-L1 peptides LQDAGVYR and VNAPYNK from recombinant PD-L1 protein spiked into HEK-293 cell lysates were done in duplicate. Analyses of the reproducibility of measurement of peptides from PD-1, PD-L1, and PD-L2 were done with four full process replicates. Because of limiting amounts of available samples, a single protein preparation and tryptic digest was fractionated for each melanoma sample and each bRPLC fraction was analyzed once by targeted MS. Calibration curves were analyzed with the QuaSAR utility as implemented in Skyline and QuaSAR was used to determine LLOD and LLOQ. MS-measured abundances of PD-1, PD-L1, and PD-L2 peptides were compared by Pearson correlation with a two-tailed *t* test for significance. MS and IHC-measured abundances of PD-L1 were compared by Spearman rank correlation with a two tailed *t* test for significance. Raw data files can be downloaded at: ftp://massive.ucsd.edu/MSV000080433. Processed Skyline files can be downloaded at https://panoramaweb.org/labkey/Liebler_IO_checkpoints.url.

## RESULTS

### 

#### 

##### Optimization of an Addressable Fractionation PRM Assay for PD-1, PD-L1, and PD-L2 and other Immune Checkpoint Proteins

Our initial analyses of FFPE melanoma sections employed a single long gradient (150 min) run of unfractionated tryptic digest. These analyses detected PD-L1 proteotypic peptides VNAPYNK and LQDAGVYR, but with low signal intensity, which would likely be inadequate for robust quantitation (supplemental Fig. S1). To improve assay sensitivity, we developed an addressable basic reverse phase fractionation (bRPLC) strategy based on the approach described by Shi *et al.* (43). We used unlabeled peptide standards of moderate (∼85%) purity to establish the elution “address” for each peptide in 12 bRPLC fractions. For most peptides, the majority of detectable signal eluted in a single bRPLC fraction. The list of proteins, peptides, and their corresponding bRPLC fractions is presented in supplemental Table S2.

Once the fraction addresses for all peptides was established, it was possible to configure assays for specific targets and combinations by selecting the corresponding bRPLC fractions for PRM analysis. To minimize effects of sample loss during fractionation, SID standards were spiked into tryptic digests immediately prior to bRPLC fractionation. For PD-1, PD-L1 and PD-L2, preliminary analyses of several target peptides and corresponding SID standards identified a set of four peptides without significant interferences in FFPE melanomas and with robust, linear calibration curves for standards spiked into HEK-293 cell lysates. Calibration curves are shown in supplemental Fig. S2.

To estimate the spike recovery of the SID assays for PD-L1 peptides VNAPYNK and LQDAGVYR, we spiked recombinant human PD-L1 protein at 0, 0.031, 0.063, 0.125, 0.25, 0.5, 1, or 5 fmol in 100 μg HEK-293 cell lysate. Duplicate response curves for both peptides were linear across the spike range with slopes of unity, which corresponded to ∼100% recovery (supplemental Fig. S3). The peptides VNAPYNK and LQDAGVYR were released from PD-L1 in a 1:1 ratio, as indicated by Pearson correlation (*r* = 1.000) for their yields in the spike-recovery experiments (supplemental Fig. S3). We next performed four full process replicate analyses of a single FFPE melanoma specimen. Coefficients of variation for the measurements were 6.6% (VNAPYNK; PD-L1), 5.8% (LQDAGVYR; PD-L1), 10.9% (TPEGLYQVTSVLR; PD-L2), and 20.1% (LAAFPEDR; PD-1) (supplemental Table S3).

##### Histologic and IHC Analysis of PD-L1 in Human FFPE Melanomas

We obtained 22 sections of FFPE melanoma tissue from pretreatment biopsies or surgical resections from patients treated with immune checkpoint inhibitors. Clinical and demographic information about the participants is listed in supplemental Table S1. Although clinical outcome information is available for these individuals, the cohort does not correspond to a clinical trial and is not designed to test hypotheses linking molecular features to outcomes. The specimens nevertheless represent multiple tissue sites typically encountered in diagnosis and therapeutic assessment of melanoma. The specimens include primary skin tumors and lung, intestinal, lymph node, and other metastases. The specimens also vary in morphology and cellular composition.

**Fig. 1.**
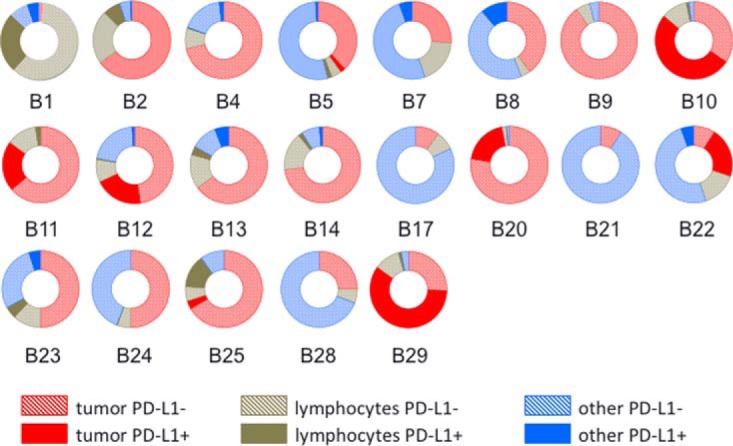
**Cellular composition and IHC PD-L1-positive fraction of cell classes in 22 melanoma samples.** Donut plots indicate tumor cells (red), lymphocytes/histiocytes (dark green) and other cell types (blue). Solid color indicates PD-L1 positive cells, hashed color indicates PD-L1 negative cells. Detailed composition of cell components and cell types that comprise the “other cell types” category are provided in supplemental Table S4.

We performed IHC analyses with the E1L3N antibody, which recognizes an intracellular epitope near the PD-L1 C terminus. IHC detected PD-L1 immunoreactivity in tumor cells, histiocytes, and tissue macrophages, as well as in peripheral nerves, ganglion cells, skeletal muscle, intestinal epithelial cells and salivary epithelial cells supplemental Table S4.) Micrographs of H&E stained sections and adjacent sections stained for PD-L1 are presented in supplemental Data set S1. The cellular composition of the samples and the fractions of each cell type component that stained positive for PD-L1 are represented in Fig. 1. The cell types represented are tumor cells, inflammatory infiltrate (mostly mononuclear phagocytic cells, including histiocytes, macrophages and dendritic cells) and other cell types and where solid coloring indicates the PD-L1 positive fractions. (Estimates for all cell types in each sample are presented in supplemental Table S4. In some samples, most of the PD-L1-positive staining was in tumor cells (*e.g.* B10 and B29), whereas in others PD-L1 positive staining was primarily in histiocytes (B1, B25) or in other cells, such as alveolar macrophages and skeletal muscle (B7 and B8).

##### MS Analysis of PD-L1, PD-1, and PD-L2

**Fig. 2.**
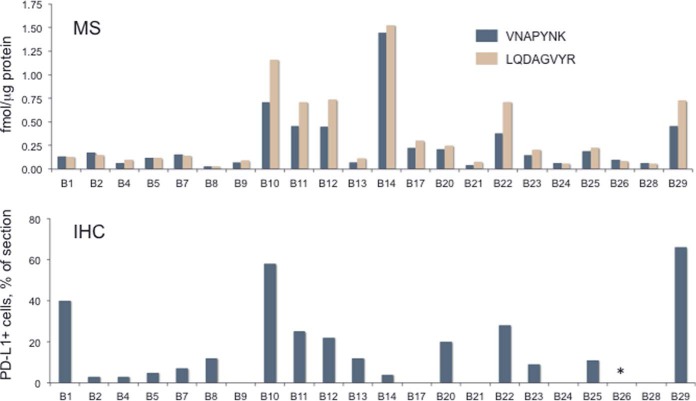
**Comparison of PD-L1 abundance in 22 melanoma samples by MS (upper panel) and IHC (lower panel).** MS measurements for PD-L1 LADAGVYR and VNAPNYK peptides are shown in fmol/mg protein. IHC measurements indicate percentage PD-L1 positive cells among all cells in each section.

We measured PD-L1 via SID assays for the peptides VNAPYNK and LQDAGVYR, as described above (Fig. 2). (PRM traces for detection and quantitation of all peptides are presented in supplemental Data set S2. Peak areas and calculations for peptide quantitation are presented in supplemental Tables S5 and S6.) Measurements for both peptides were above the lower limit of quantitation (LLOQ) in all samples and ranged 50-fold, from ∼0.03 to 1.5 fmol/μg protein. VNAPYNK and LQDAGVYR measurements were highly correlated (r^2^ = 0.9161, *p* < 0.0001), but VNAPYNK was often measured at lower abundance (mean 0.85 ± 0.22). This may reflect variable loss of the lysine C-terminal peptide VNAPYNK because of formalin fixation, as we have reported previously (34). Comparison of the PD-L1 abundances measured by MS with total cellular PD-L1 (Fig. 2) detected by IHC indicated a significant correlation between the platforms (Spearman *r* = 0.5841, *p* = 0.0054). A notable outlier was sample B14 (from a small intestinal metastasis), which indicated little PD-L1 by IHC (3% of entire section), but the highest MS-measured PD-L1 abundance of all the samples.

**Fig. 3.**
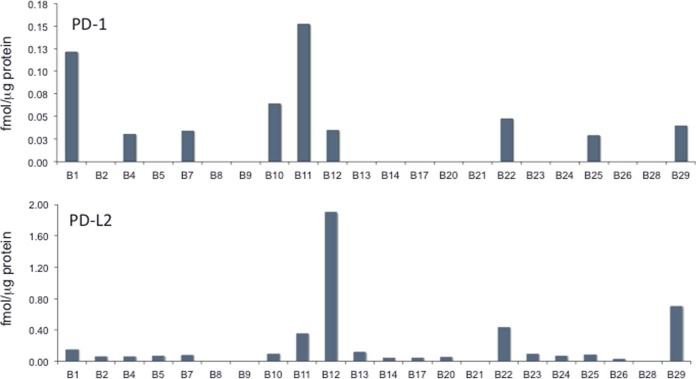
**PD-1 (upper panel) and PD-L2 (lower panel) abundance in 22 melanoma samples measured by MS.**

**Fig. 4.**
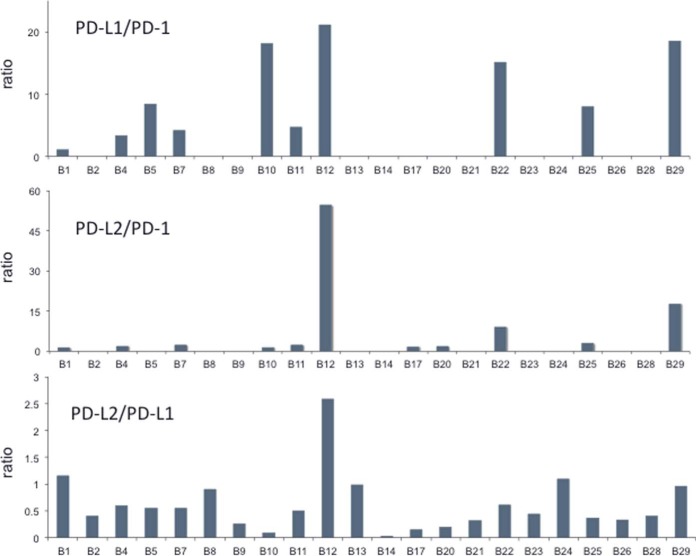
**Abundance ratios for PD-L1:PD-1 (upper panel), PD-L2:PD-1 (middle panel) and PD-L2:PD-L1 (lower panel) in 22 melanoma samples measured by MS.** Ratios are presented only for samples in which both proteins were quantifiable above the LLOQ. Calculated ratios are shown in supplemental Table S7.

PD-1 was quantifiable via the peptide LAAFPEDR in 9 of the 22 samples and measured values ranged 5-fold from 0.03 to 0.15 fmol/μg protein (Fig. 3). PD-1 abundance was weakly correlated (r^2^ = 0.3057, *p* = 0.009) with the fraction of lymphocytes/histiocytes in sections. PD-L1 abundance was up to 20-fold greater than PD-1 abundance in samples where both were quantifiable (Fig. 4, supplemental Table S7), but the abundances were not significantly correlated (r^2^ = 0.062, *p* = 0.264). PD-L2 was quantifiable via the peptide TPEGLYQVTSVLR in 18 of the 22 samples and measured values ranged 95-fold from 0.03 to 1.90 fmol/μg protein (Fig. 3). The ratio of PD-L2 to PD-L1 abundance ranged from 0.03 to 2.58 (Fig. 4). In 10 samples, PD-L2 was present at more than half the level of PD-L1. PD-L2 and PD-L1 abundances were not significantly correlated (r^2^ = 0.118, *p* = 0.1169).

##### MS Analysis of PD-L1 N-glycosylation

PD-L1 has been reported to be N-glycosylated at several residues (29, 44). We first analyzed a purchased recombinant human PD-L1 containing residues 1–239 of the extracellular domain fused to the Fc region of human IgG1 at the C terminus. Tryptic digestion of the protein, followed by treatment of the digest with PNGase-F hydrolyzed N-glycosylated peptides to sequences with aspartate residues at sites of glycosylated asparagines. This analysis identified N35, N192, and N200 as sites of N-glycosylation in the recombinant protein (supplemental Figs. S4–S6). MS/MS analysis of the tryptic N-glycopeptide forms of LFNVTSTLR, which contains the N192 residue, identified 5 glycan forms (supplemental Figs. S7–S11). The MS/MS spectra of putative glycopeptides contained characteristic low molecular weight oxonium ions, including *m*/*z* 204 (HexNAc+), *m*/*z* 366 (Hex-HexNAc+), and *m*/*z* 528 (Hex-Hex-HexNAc+). Candidate structures generated from the precursor ion *m*/*z* using the GlycoMod tool (http://web.expasy.org/glycomod/) were high-mannose glycans. Although these structures presented in supplemental Figs. S7–S11 are consistent with the MS data, the number and exact identities of isomeric structures for the high mannose glycans cannot be determined from the MS data alone.

**Fig. 5.**
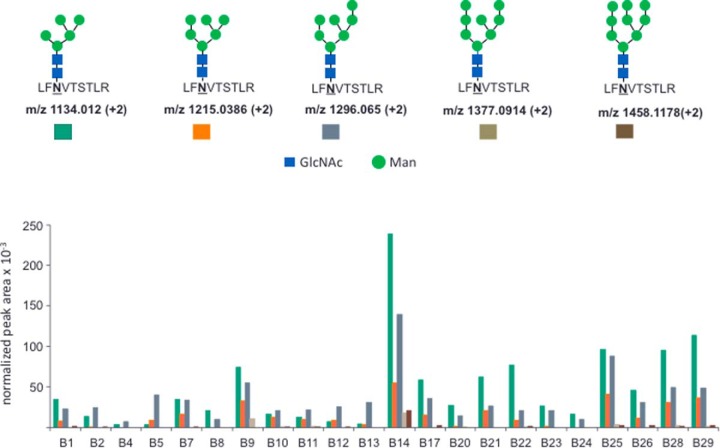
**Analysis of PD-L1 N-glycosylation at position N192 in 22 melanoma samples.** The upper panel depicts structures and precursor *m*/*z* for the five LFNVTSTLR glycopeptides detected in all samples. The lower panel depicts relative quantitation of the five glycopeptides, as indicated by color key. Relative quantitation was calculated from the ratio of peak area for the most abundant MS2 product ion for all glycopeptides (*m*/*z* 1253.6806), which was normalized to measured amount of PD-L1 peptide LQDAGVYR for each sample. Structures for the Man_6_, Man_7_ and Man_8_ glycans are representative of multiple possible positional isomers.

We detected these five N192-glycopeptide forms in the tryptic digests of the 22 melanomas described above (Fig. 5). MS/MS spectra matched those of five N192 glycopeptides detected in the recombinant PD-L1 protein (supplemental Figs. S7–S11). We also found that all of the glycopeptide forms eluted together in the same bRPLC fraction (Fraction 12, supplemental Table S2). This enabled us to design a relative quantitation approach in which the integrated peak areas for the highest intensity MS/MS product ion (LFN(HexNAc)VTSTLR^+^, *m*/*z* 1253.6806) were normalized to the PD-L1 protein amounts calculated from SID measurements of the LQDAGVYR peptide in each sample. PD-L1 N192 glycopeptides were detectable in all the 22 melanomas, with the Man5, Man6, and Man7 structures having the highest relative abundance (supplemental Table S8). The relative abundances for each glycopeptide varied ∼50-fold (Fig. 5). Sample B14 had the most abundantly N-glycosylated PD-L1. This sample also had the highest PD-L1 protein abundance (Fig. 2), but had very low PD-L1 positive IHC staining—only 3% of the cells in the section and no staining in tumor cells (Fig. 1, supplemental Table S4). Moreover, samples B9, B17, B21, and B28 had no IHC-detectable PD-L1 (Fig. 2), but did have MS-detectable PD-L1 (Fig. 2) and N-glycosylated PD-L1 (Fig. 5).

##### MS Analysis of Other Immune Checkpoint and Coregulator Proteins

**Fig. 6.**
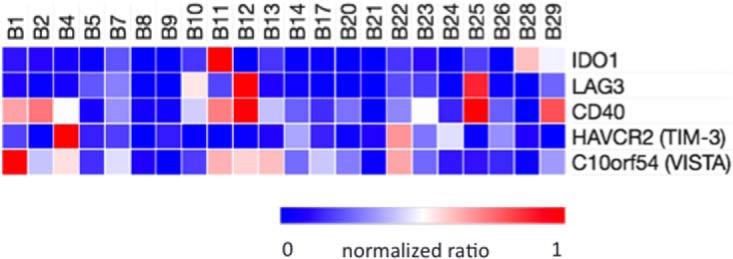
**Heatmap representation of relative quantitation of IDO1, LAG3, HAVCR2 (TIM-3), C10orf54 (VISTA) and CD40 by LRP PRM analysis of proteotypic peptides.** Signals from the three most abundant PRM transitions for each target peptide were normalized to integrated signal for the three most abundant transitions for an LRP peptide present in the same bRPLC fraction. The bRPLC fractions, LRP peptides and transitions are presented in supplemental Table S2.

To explore the possibility of multiplexed analysis of a broader group of protein regulators of the tumor-immune interface, we configured PRM assays for the immune checkpoint proteins indolamine-2,3-dioxygenase 1 (IDO1), lymphocyte activating 3 (LAG3), hepatitis A virus cellular receptor 2 (HAVCR2 (TIM-3)), chromosome 10 open reading frame 54 (C10orf54 (VISTA)), and CD40 molecule (CD40). We adapted the labeled reference peptide (LRP) method (40), in which a single heavy isotope-labeled peptide is used for normalization of multiple other peptides. Because the target peptides for all five proteins eluted in different bRPLC fractions, we employed isotope-labeled LRP standards that eluted in the same fractions (supplemental Table S2). Comparisons with the LRP method are based on ratios of the integrated transitions to those of the standard. This permits comparison of relative amounts between samples, but not molar amounts of different proteins in the same sample. (Peak areas for target peptides, LRP standards and normalized peak areas are shown in supplemental Tables S9 and S10). Nevertheless, the LRP analyses detected IDO1, LAG3, TIM-3, VISTA, and CD40 in the 22 melanomas (Fig. 6). These measurements indicated abundance differences between samples of approximately an order of magnitude for all five proteins. Moreover, the patterns of expression for these proteins were distinct for each and did not correlate with those for PD-1, PD-L1, and PD-L2.

## DISCUSSION

Quantitative assessment of key proteins that control the tumor-immune interface is one of the most formidable analytical challenges in immunotherapeutics. Here we demonstrate that quantitative MS can measure multiple immune checkpoint proteins, identify and quantify N-glycosylated forms, and determine stoichiometric relationships between immune checkpoint binding partners. Our MS measurements of PD-L1 are largely concordant with IHC assessments, but the differences reveal additional information not accessible through IHC. Histological characterization is central to the assessment of cancers and IHC will remain an essential element of diagnostics for immuno-therapeutics. However, our findings demonstrate that quantitative MS measurement of immune checkpoint proteins provides a valuable layer of new information that can substantially advance therapeutic and diagnostic development in immuno-oncology.

Targeted measurements of the peptides LQDAGVYR and VNAPYNK provided similar measurements of PD-L1 in FFPE melanoma sections. Although the peptides were released in equal amounts from recombinant PD-L1 in a cell lysate matrix, VNAPYNK was measured in FFPE sections at, on average, a 15% lower abundance than LQDAGVYR. This effect is consistent with our previous observations on how formaldehyde crosslinks impact the yields of lysine C-terminal tryptic peptides (34). Nevertheless, the two peptides provide consistent measurements of PD-L1 abundance differences across the sample set.

A comparison between the MS and IHC results should consider the different characteristics of the measurements. MS measures molar quantities of PD-L1, whereas IHC measures percentages of cells in a field that exceed a minimum threshold staining intensity. Percentages of cells that exceed the threshold may be proportional to the overall molar amount of PD-L1 in a sample. This was confirmed by the moderate rank correlation (Spearman *r* = 0.5841) for measurements with the two platforms. Nevertheless, cells that exceed a staining intensity threshold may contain substantially different amounts of PD-L1. For example, sample B29 had the highest PD-L1 positive percentage of cells (61%), but had only the fourth highest PD-L1 MS measurement. IHC staining intensity for the tumor cells was only modestly above the detection threshold. Similarly, sample B1 had the third highest IHC rank, with 31% PD-L1 positive cells, but was the 13th highest MS rank for PD-L1 abundance. This specimen showed extended areas with low intensity staining. Thus, although IHC can distinguish tissues with PD-L1 staining above or below a threshold, IHC is nevertheless a poor measure of PD-L1 quantity in tissues.

A key difference between the IHC and MS platforms is that only IHC enables assignment of PD-L1 staining to specific cell types in a section. We found PD-L1 staining in multiple cell types, including tumor cells, histiocytes, macrophages, skeletal muscle, epithelia, and nerve cells. The cell types displaying PD-L1 positive staining also varied considerably. For example, samples B10, B11, B12, B20, and B29 all had >65% tumor cells, which accounted for almost all the PD-L1 positive staining. On the other hand, in samples B1 and B2, both from lymph node metastases, the principal PD-L1 positive staining was in histiocytes. In samples B7 (lung metastasis) and B8 (primary tumor), PD-L1 positive staining was localized to macrophages and skeletal muscle, respectively. Comparison of the IHC and MS data indicates that the highest MS measurements usually corresponded to samples with a significant fraction of PD-L1 positive tumor cells. Nevertheless, the key point of these comparisons is that the cellular source of MS-measured PD-L1 cannot be assigned to any particular cell type without accompanying IHC measurement.

Our studies confirmed PD-L1 N-glycosylation, which has been reported previously (29, 44), although sites of modification and glycan structures had not been characterized in melanomas. We found consistent N-glycosylation at position N192 with high mannose structures, which were detected directly in tryptic digests. We could estimate the abundance of the glycoforms based on the intensity of the major glycopeptide MS/MS fragment ion shared by all of the glycopeptides. Further, we could normalize these measurements to the quantified amount of PD-L1 in each sample. These normalized measurements allowed comparison of the fractional glycosylation at position N-192 for PD-L1 across the sample set.

These comparisons revealed considerable variability in fractional N192 glycosylation of PD-L1 in the samples. The unmodified N192-containing peptide LFNVTSTLR was not detected in any of the samples in the bRPLC fraction containing an isotope labeled synthetic LFNVTSTLR standard (data not shown). Sample B14 had the highest degree of N192 glycosylation, together with the highest PD-L1 protein abundance, but with little detectable PD-L1 by IHC. This sample was a small intestinal metastasis containing 73% tumor cells, but with PD-L1 positive IHC staining only in histiocytes, peripheral nerve and ganglion cells. The discordance between MS and IHC measurements, together with the high degree of N-glycosylation suggests that the posttranslational modification interfered with recognition by the E1L3N antibody. We also found that samples B9, B17, B21, and B28 contained significant N192 PD-L1 glycosylation, but no PD-L1 was detected by IHC.

Interestingly, the E1L3N antibody recognizes an intracellular, C-terminal PD-L1 epitope, whereas the glycosylated residue N192 is in the PD-L1 extracellular domain. Extracellular modification thus may alter an intracellular C-terminal PD-L1 epitope. Position N192 is not contained within the PD-1 recognition sequence, which includes residues between I54 and R125 (45) or the recognition sequence for the recently approved PD-L1 inhibitor avelumab, which includes residues between Y56 and S117 (46). It is also not clear whether N192 glycosylation would affect recognition of PD-L1 by other antibodies used in diagnostic tests, such as the Dako 22C3 and 28–8 antibodies (15). Any effect of glycosylation at N192 on these other interactions would be difficult to predict and would require experimental verification. Nevertheless, these observations suggest testable hypotheses for the response of “PD-L1-negative” cancers to immunotherapeutics—that IHC negative tumors may contain functionally competent PD-L1 that is significantly N-glycosylated and therefore not detectable by some antibodies.

Targeted MS affords a systematic approach to develop assays for proteins for which high quality antibody reagents are unavailable. PD-L2 has been recognized as a regulator of PD-1 with higher affinity than PD-L1 (25, 26). However, PD-L2 has been suggested to be of lower importance because of apparent low expression in solid tumors (27), although the poor quality of available antibodies has been noted recently (28). Our MS measurements demonstrate PD-L2 expression in melanomas at levels comparable to PD-L1 (Fig. 3). PD-L2 was more abundant than PD-L1 in sample B12, was at equal abundance with PD-L1 in five other samples and was at least half the level of PD-L1 in half of the entire sample set (Fig. 4). Given that PD-L2 is reported to bind PD-1 with 2–6-fold higher affinity than does PD-L1 (26), our data suggests that PD-L2 is present in sufficient abundance to contribute to PD-1-dependent T cell downregulation. PD-L2-dependent effects thus may impact the responsiveness of cancers to immune checkpoint therapeutics, particularly for drugs directed against PD-L1. We also note that both PD-L1 and PD-L2 were more abundant than PD-1 in all samples for which abundance ratios could be measured.

The immune-tumor interface is known to be regulated by at least a dozen other sets of protein switches, which can modulate PD-1/PD-L1/PD-L2 mediated signaling and provide independent regulatory signals (2). Many of these proteins are targets for new therapeutics in preclinical development and clinical trials (48). We selected IDO1, LAG3, HAVCR2 (TIM-3), C10orf54 (VISTA), and CD40 as representative of this growing group of drug targets and coregulators. IDO1 negatively regulates T cells in the tumor microenvironment by catabolizing tryptophan, thereby inducing an amino acid starvation response to which T cells are highly sensitive (49). LAG3 and HAVCR2 (TIM-3) are T-cell receptors that mediate inhibition of T-cell activation (50, 51). C10orf54 (VISTA) is a V domain Ig family suppressor of T-cell activation (52, 53). CD40 is an activator of cytotoxic T cells (54).

To explore the feasibility of measuring these proteins in melanomas, we used the LRP method for quantitative comparisons. The LRP approach does not require isotope labeled standards for quantitation, but can instead utilize other labeled peptide standards for signal normalization (40). Peptide signals for the five additional proteins were normalized to signals from isotope-labeled standards for PD-1, PD-L1, and PD-L2 peptides that eluted in the same bRPLC fractions. Although the LRP measurements do not yield molar amounts, the normalized signals enabled relative quantitative comparisons of abundance across the sample set. Our measurements revealed that IDO1, LAG3, HAVCR2 (TIM-3), C10orf54 (VISTA), and CD40 all display different abundance patterns across the samples and that none are predicted by measurements of PD-1, PD-L1, or PD-L2. This suggests that these immuno-modulators function independently and that measurement of these molecules provides independent, new information about the tumor-immune interface.

Full development of a suite of several dozen SID MS assays for components of the tumor-immune interface is clearly feasible, particularly based on recent work by us (35, 36) and others (55, 56). Moreover, the National Cancer Institute Clinical Proteomic Tumor Analysis Consortium has demonstrated the ability of multiple laboratories to implement standardized, reproducible, and robust targeted peptide quantitative MS analysis platforms (38, 57, 58).

The combination of MS analyses with IHC data could overcome limitations of IHC as the sole protein biomarker measurement platform in immunotherapeutic research and diagnostics. Moreover, a targeted MS platform to analyze multiple critical immune checkpoint and co-regulator proteins could address important, unresolved issues through retrospective analyses of well-annotated, archival specimens from completed immunotherapeutics clinical trials. For example, in trials of PD-1 inhibitors for melanoma, the KEYNOTE-001 trial (pembrolizumab) (59) and the CheckMate 067 trial (nivolumab) (5) both reported benefit in patients who were either positive and negative for PD-L1 in two different IHC tests. The results raise the question of why IHC PD-L1 negative patients responded to the drugs. Our results suggest testable hypotheses: (1) IHC failed to detect functionally competent PD-L1 because of posttranslational modification, or (2) expression of PD-L2 may have mediated T-cell suppression in the absence of PD-L1. Given that over 1000 clinical trials with 200,000+ participants in over 35 cancers have been initiated for immune checkpoint therapeutics (www.clinicaltrials.gov), such retrospective studies offer a tremendous opportunity to advance this field.

## DATA AVAILABILITY

Raw data files can be downloaded at: ftp://massive.ucsd.edu/MSV000080433. Processed Skyline files can be downloaded at https://panoramaweb.org/labkey/Liebler_IO_checkpoints.url.

## Supplementary Material

Supplemental Data

## Footnotes

^1^ The abbreviations used are:

PD-L1: programmed cell death- ligand 1
AmBic: ammonium bicarbonate
AGC: automatic gain control
bRPLC: basic reverse phase liquid chromatography
BCA: bicinchoninic acid
BSA: bovine serum albumin
DTT: dithiothreitol
FFPE: formalin-fixed, paraffin-embedded
H&E: hematoxylin and eosin
HPLC: high performance liquid chromatography
IHC: immunohistochemistry
LC-MS/MS: liquid chromatography-tandem mass spectrometry
LLOD: lower limit of detection
LLOQ: lower limit of quantitation
LRP: labeled reference peptide
MS/MS: tandem mass spectrometry
PBS: phosphate buffered saline
PNGase F: protein N-glycanase F
PRM: parallel reaction monitoring
RT: room temperature
SID: stable isotope dilution
TBST: tris-buffered saline plus 0.05% tween-20.
