# Supplementary material for: Quantitative Mass Spectrometry Analysis of PD-L1 Protein Expression, N-glycosylation and Expression Stoichiometry with PD-1 and PD-L2 in Human Melanoma
Source: Mol Cell Proteomics. 2017 May 25;16(10):1705–17. doi: 10.1074/mcp.RA117.000037 (PMC5629259; doi:10.1074/mcp.RA117.000037)
Supplement: Supplemental Data [file supp_16_10_1705__index.html]

Quantitative Mass Spectrometry Analysis of PD-L1 Protein Expression, N-glycosylation and Expression Stoichiometry with PD-1 and PD-L2 in Human Melanoma — MS of Immune Checkpoints — Supplemental Data 

# Quantitative Mass Spectrometry Analysis of PD-L1 Protein Expression, *N*-glycosylation and Expression Stoichiometry with PD-1 and PD-L2 in Human Melanoma

## Supplemental Data

- Supplemental Dataset 2 - PRM traces for all quantified peptides
- Supplemental Tables - Supplemental Tables
- Supplemental Figures - Supplemental Figures
- Supplemental Dataset 1 - H&E and IHC images
