# Supplementary material for: Quantitative Mass Spectrometry Analysis of PD-L1 Protein Expression, N-glycosylation and Expression Stoichiometry with PD-1 and PD-L2 in Human Melanoma
Source: Mol Cell Proteomics. 2017 May 25;16(10):1705–17. doi: 10.1074/mcp.RA117.000037 (PMC5629259; doi:10.1074/mcp.RA117.000037)

Morales-Betanzos et al.

Supplemental Dataset 2

PRM traces for detection of target peptides in all samples

- Part I. PD-1, PD-L1, PD-L2 (PRM with stable isotope dilution)
- Part II. IDO1, LAG3, HAVCR2 (TIM-3), C10orf54 (VISTA), CD40 (PRM with labeled reference peptide normalization)

Part I. Quantitation of PD-1, PD-L1, and PD-L2 by PRM with stable isotope dilution

PD1

PD-1 was quantified via the proteotypic peptide LAAFPEDR. PRM transitions traces for the endogenous LAAFPEDR peptide and the (13C15N standard for each sample are shown in the following 3 pages.

PD1 endogenous= LAAFPEDR **standard** = LAAFPED**R** (C^{13} N^{15})

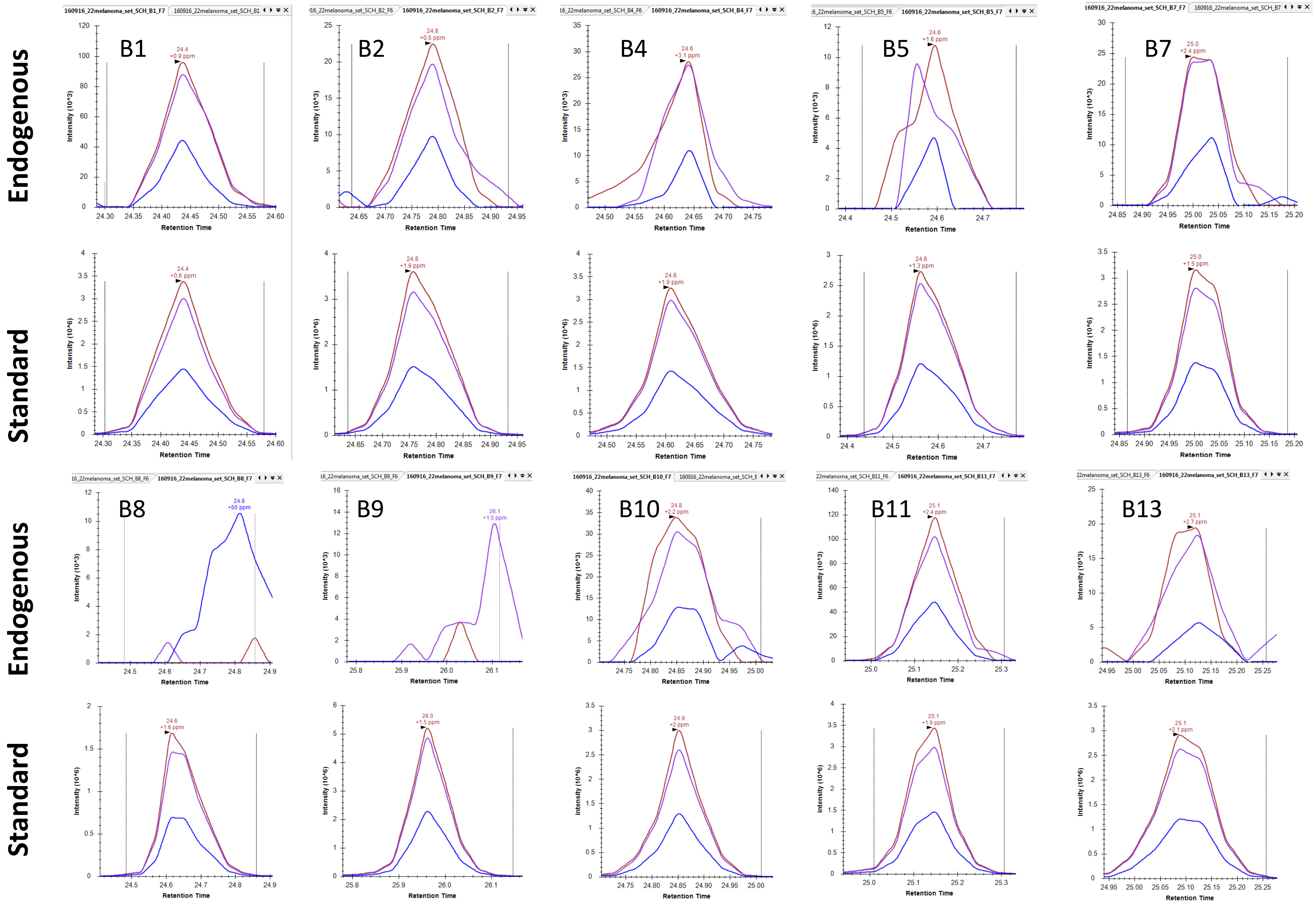

PD1 endogenous= LAAFPEDR **standard** = LAAFPED**R** (C^{13} N^{15})

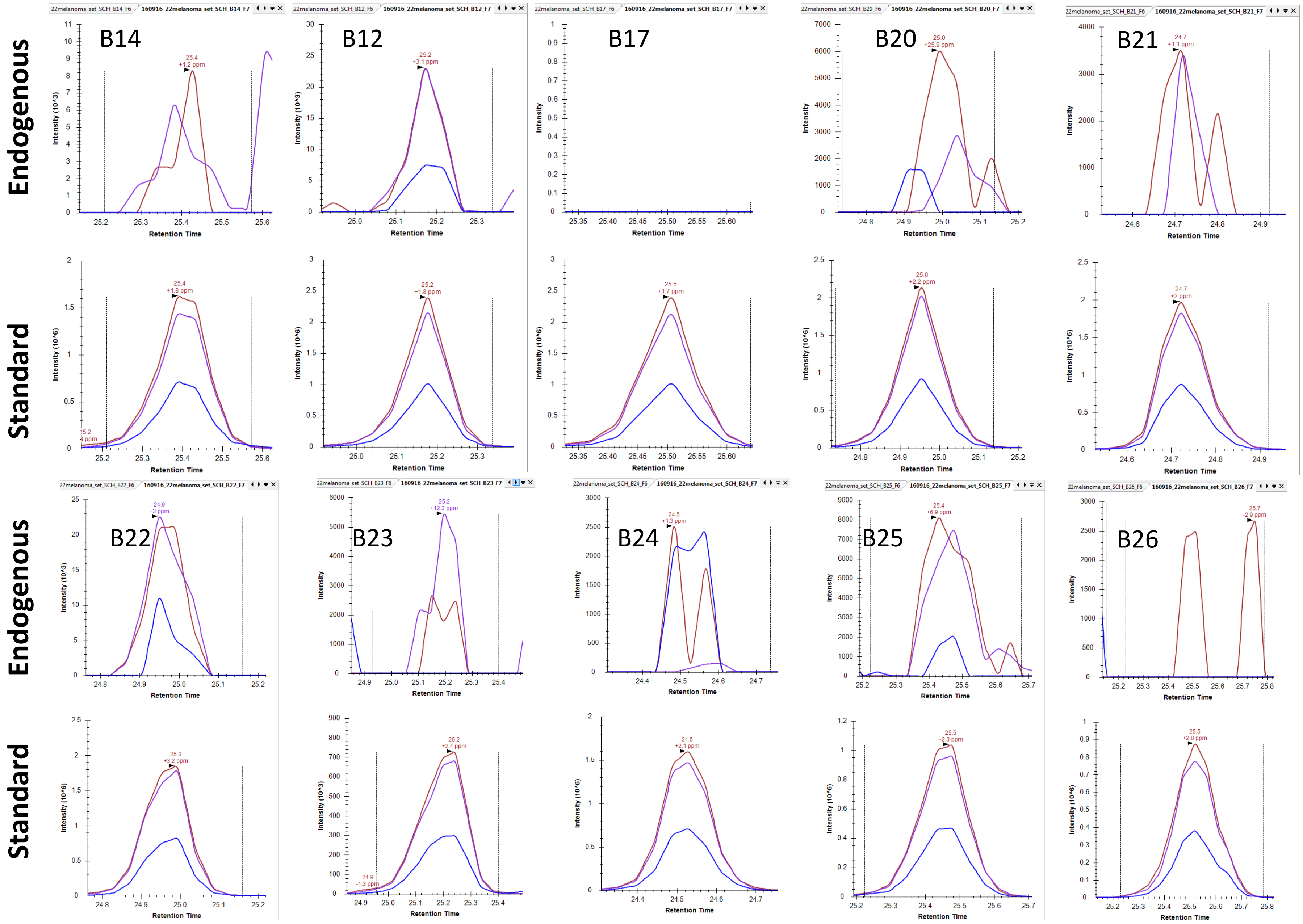

PD1 endogenous= LAAFPEDR **standard** = LAAFPED**R** (C^{13} N^{15})

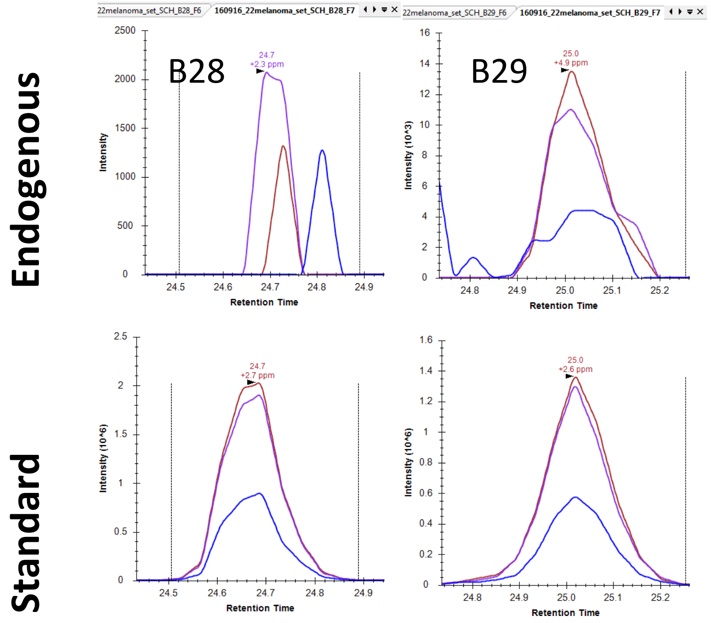

PD-L1

PD-L1 was quantified via the proteotypic peptides LQDAGVYR and VNAPYNK. PRM transitions traces for the endogenous LQDAGVYR and VNAPYNK peptides and the 13C15N standard for each sample are shown in the following 3 pages.

PDL1 endogenous= LQDAGVYR **standard** = LQDAGVY**R** (C^{13} N^{15})

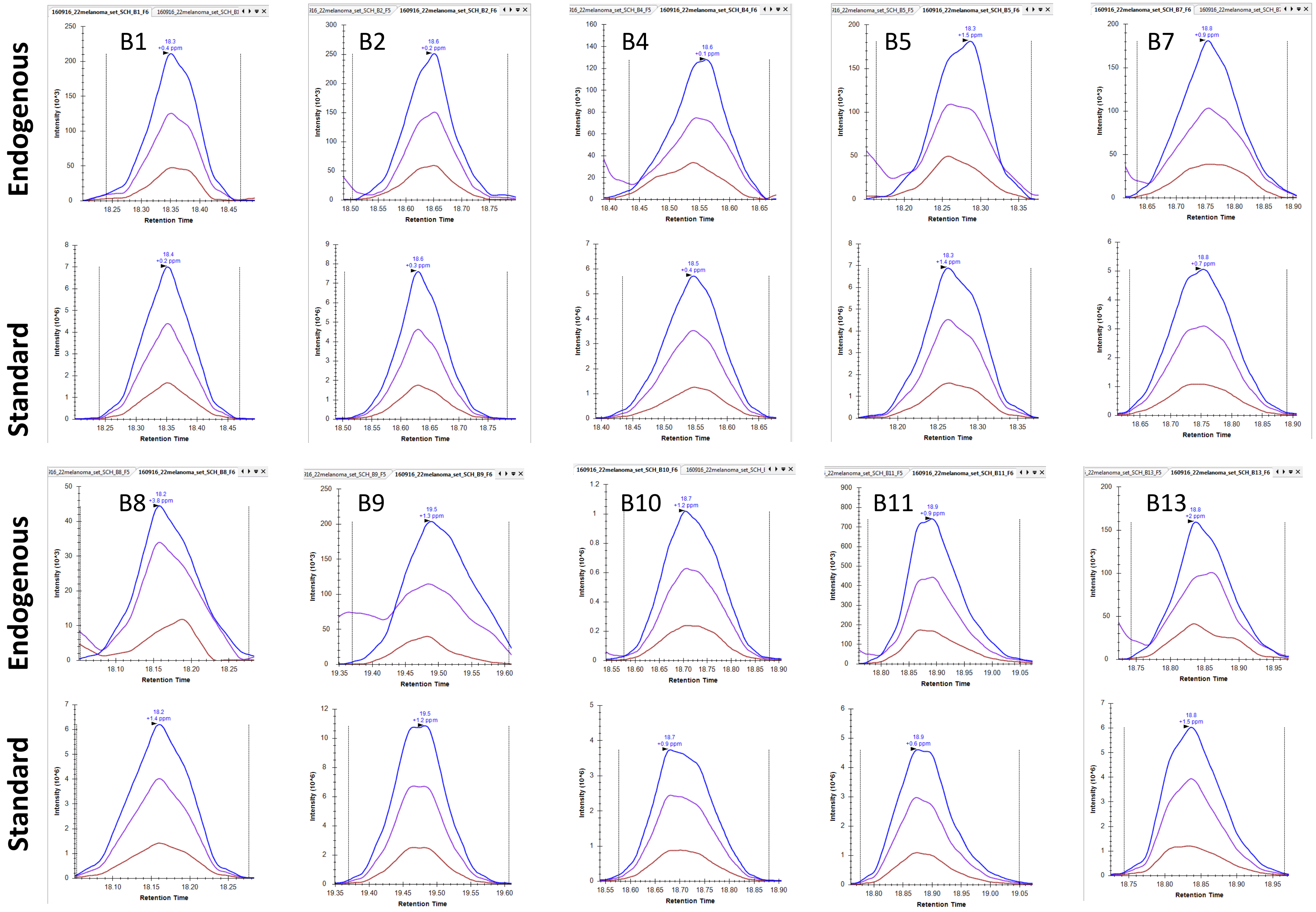

PDL1 endogenous= LQDAGVYR **standard** = LQDAGVY**R** (C^{13} N^{15})

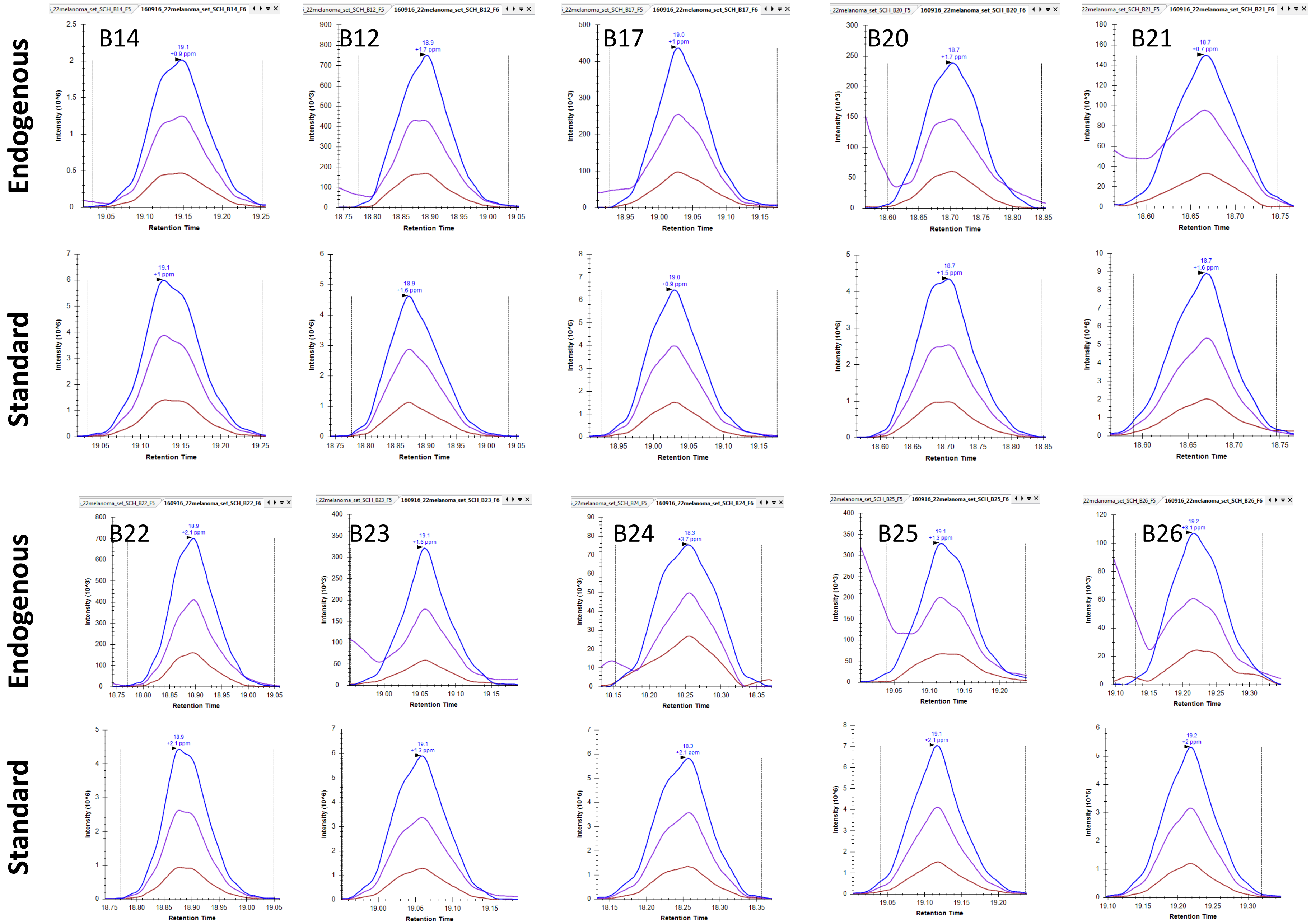

PDL1 endogenous= LQDAGVYR **standard** = LQDAGVY**R** (C^{13} N^{15})

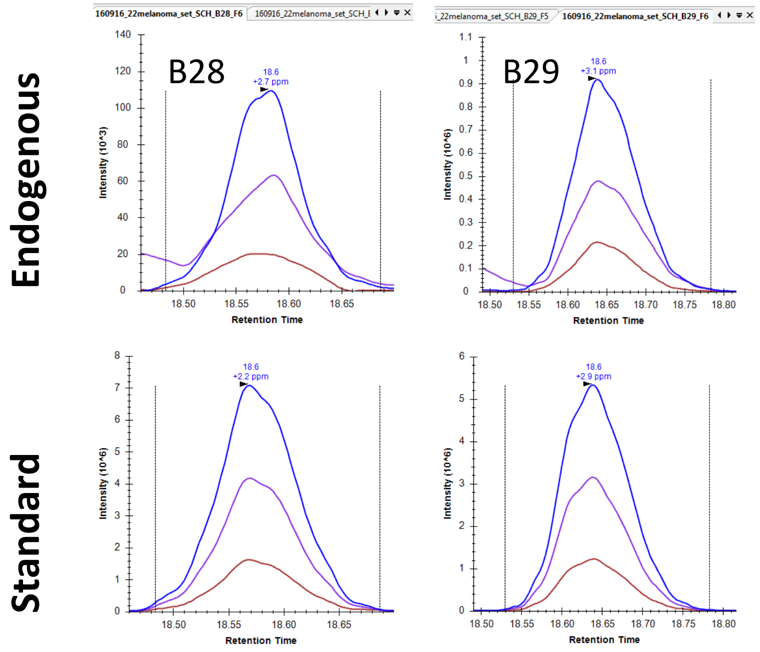

PDL1 endogenous= VNAPYNK **standard** = VNAPYN**K**(C^{13} N^{15})

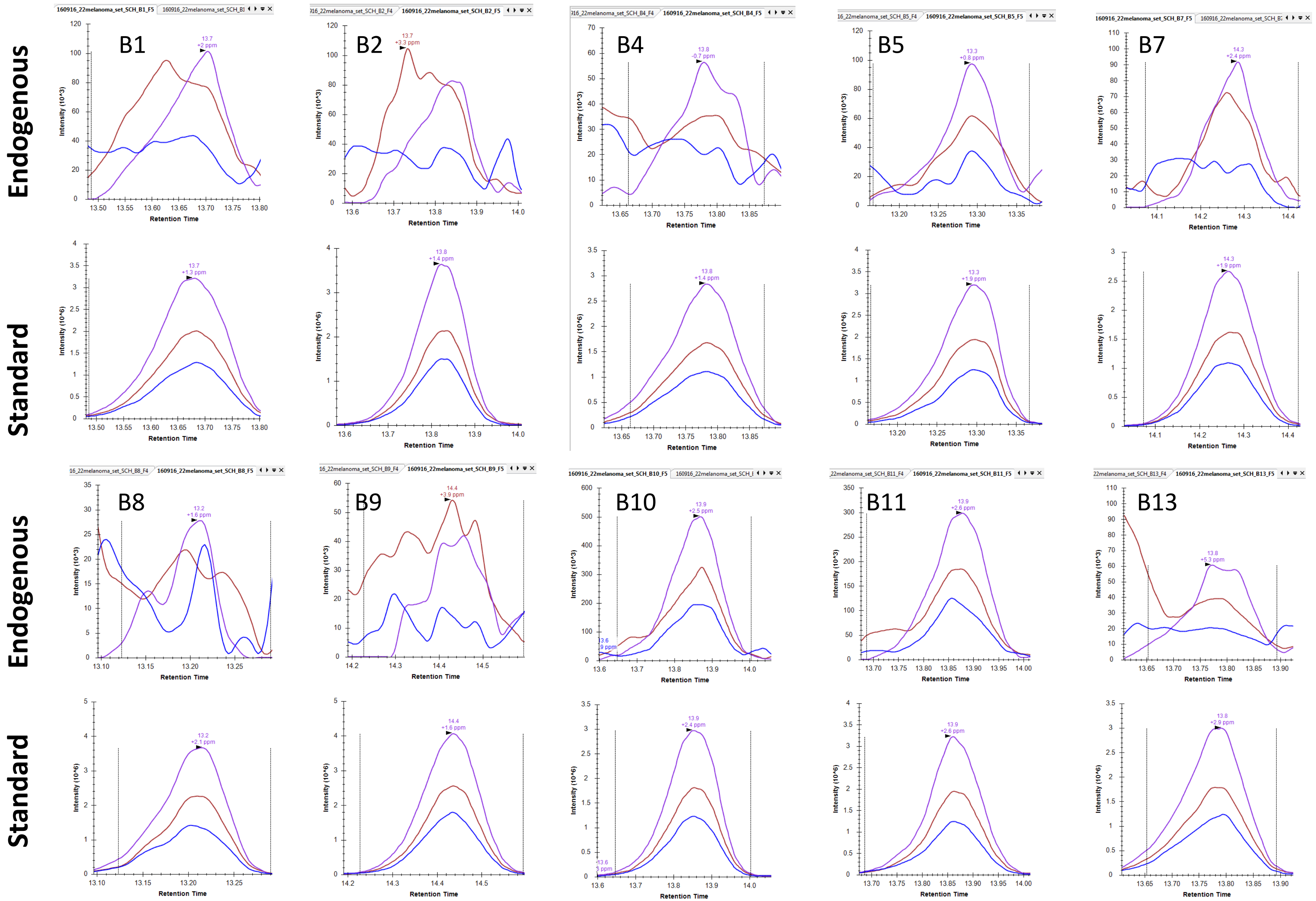

PDL1 endogenous= VNAPYNK **standard** = VNAPYN**K**(C^{13} N^{15})

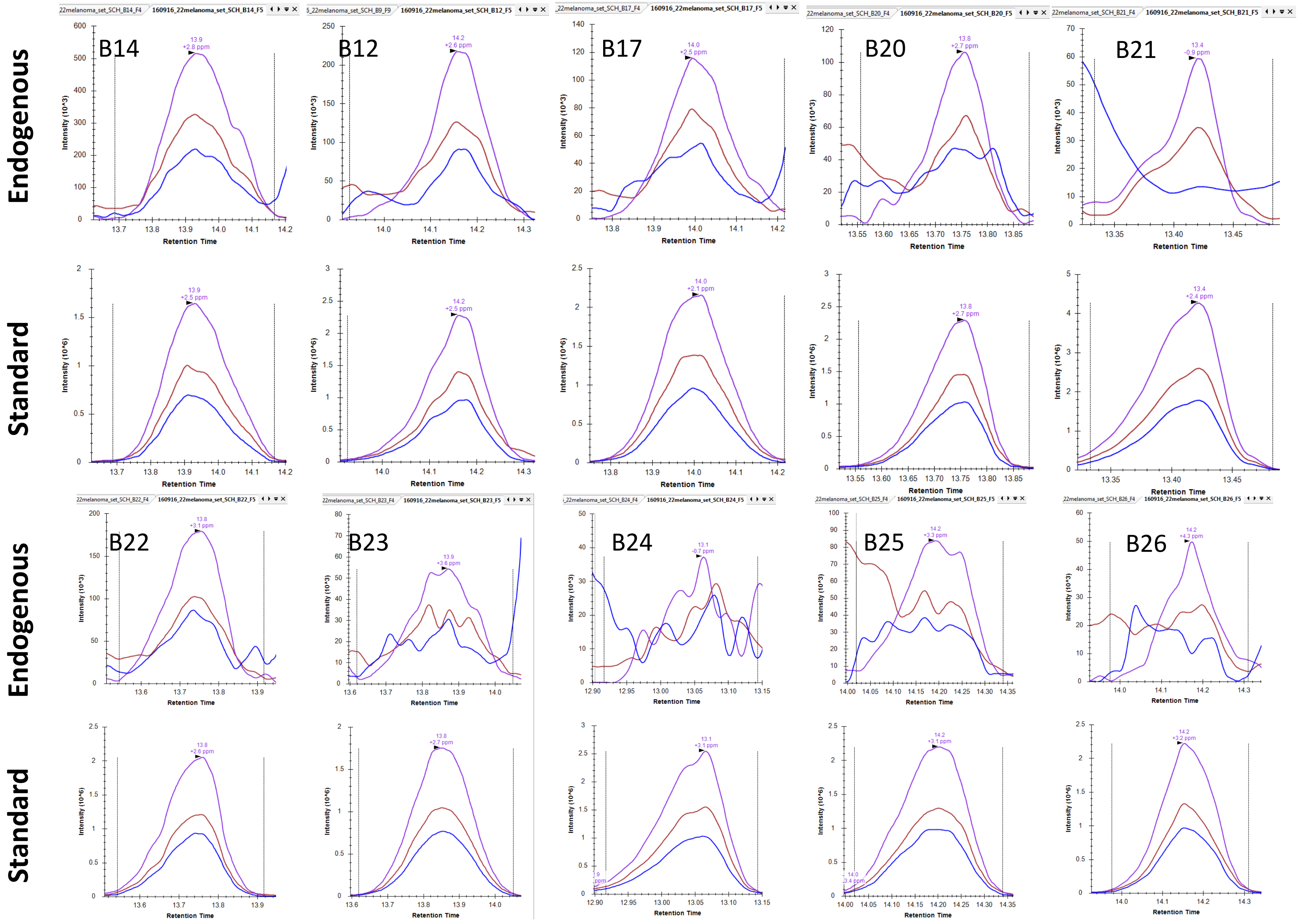

PDL1 endogenous= VNAPYNK **standard** = VNAPYN**K**(C^{13} N^{15})

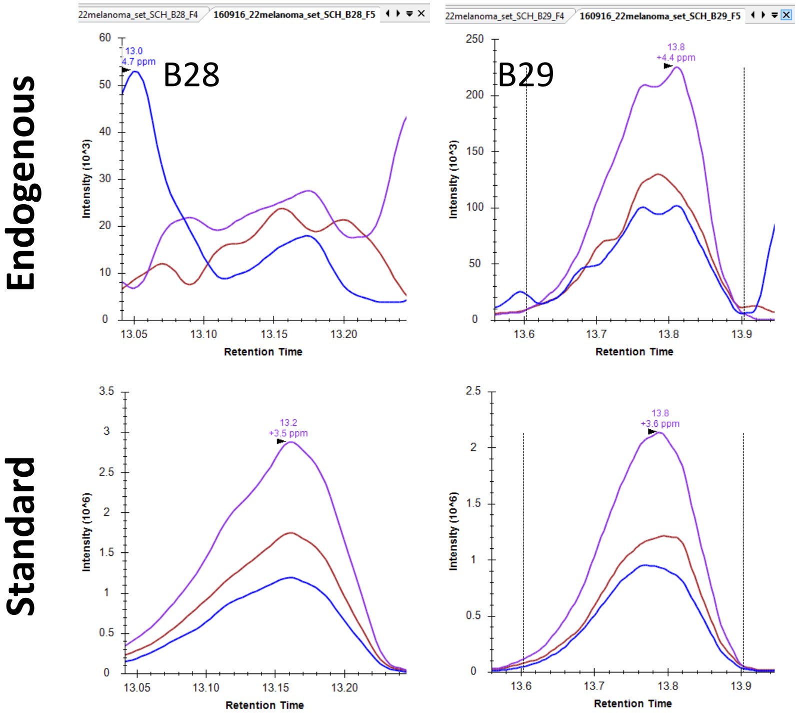

PD-L2

PD-L2 was quantified via the proteotypic peptide TPEGLYQVTSVLR. PRM transitions traces for the endogenous TPEGLYQVTSVLR peptide and the (13C15N standard for each sample are shown in the following 3 pages.

PDL2 endogenous= TPEGLYQVTSVLR **standard** = TPEGLYQVTSVL**R** (C^{13} N^{15})

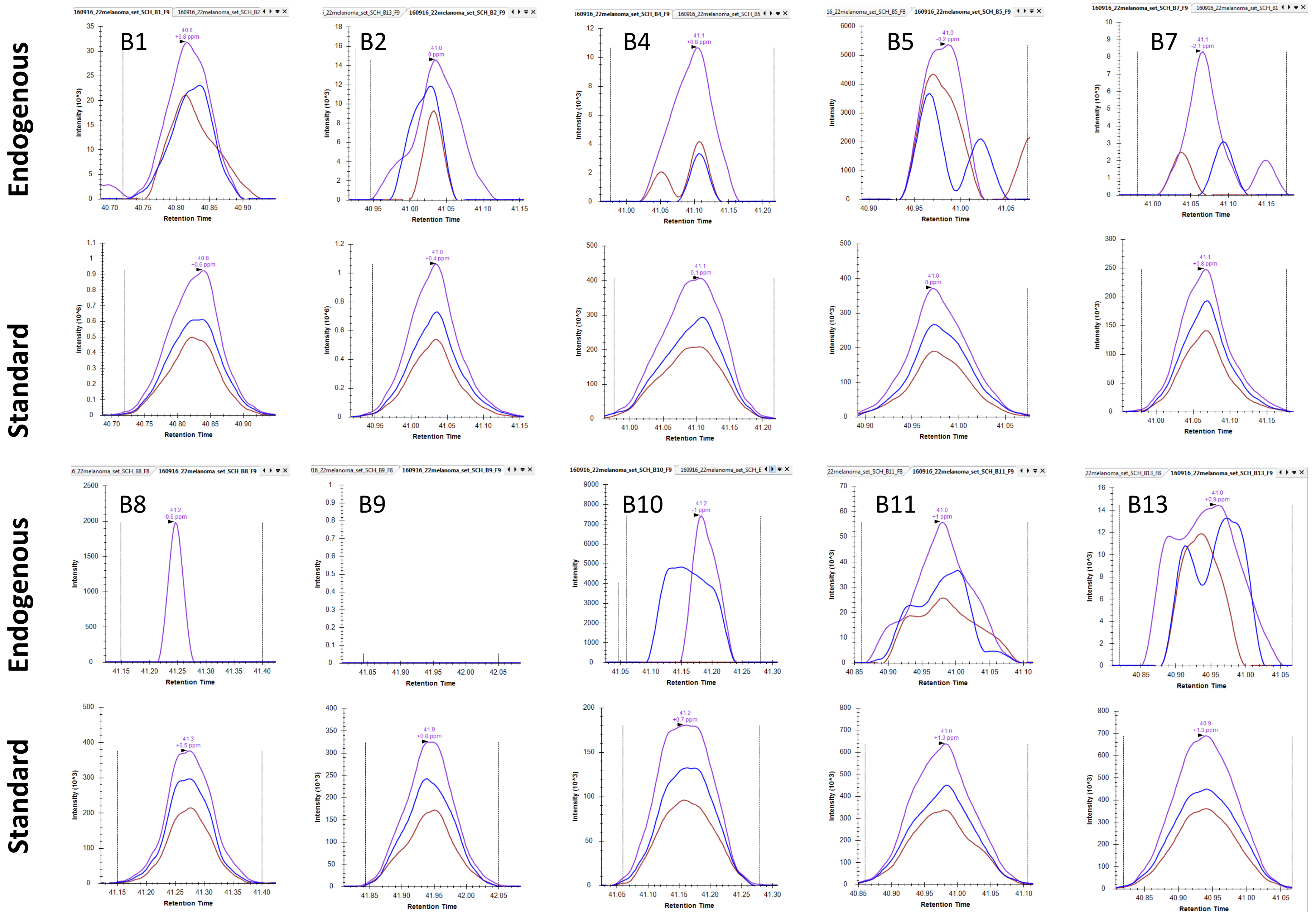

PDL2 endogenous= TPEGLYQVTSVLR **standard** = TPEGLYQVTSVL**R** (C^{13} N^{15})

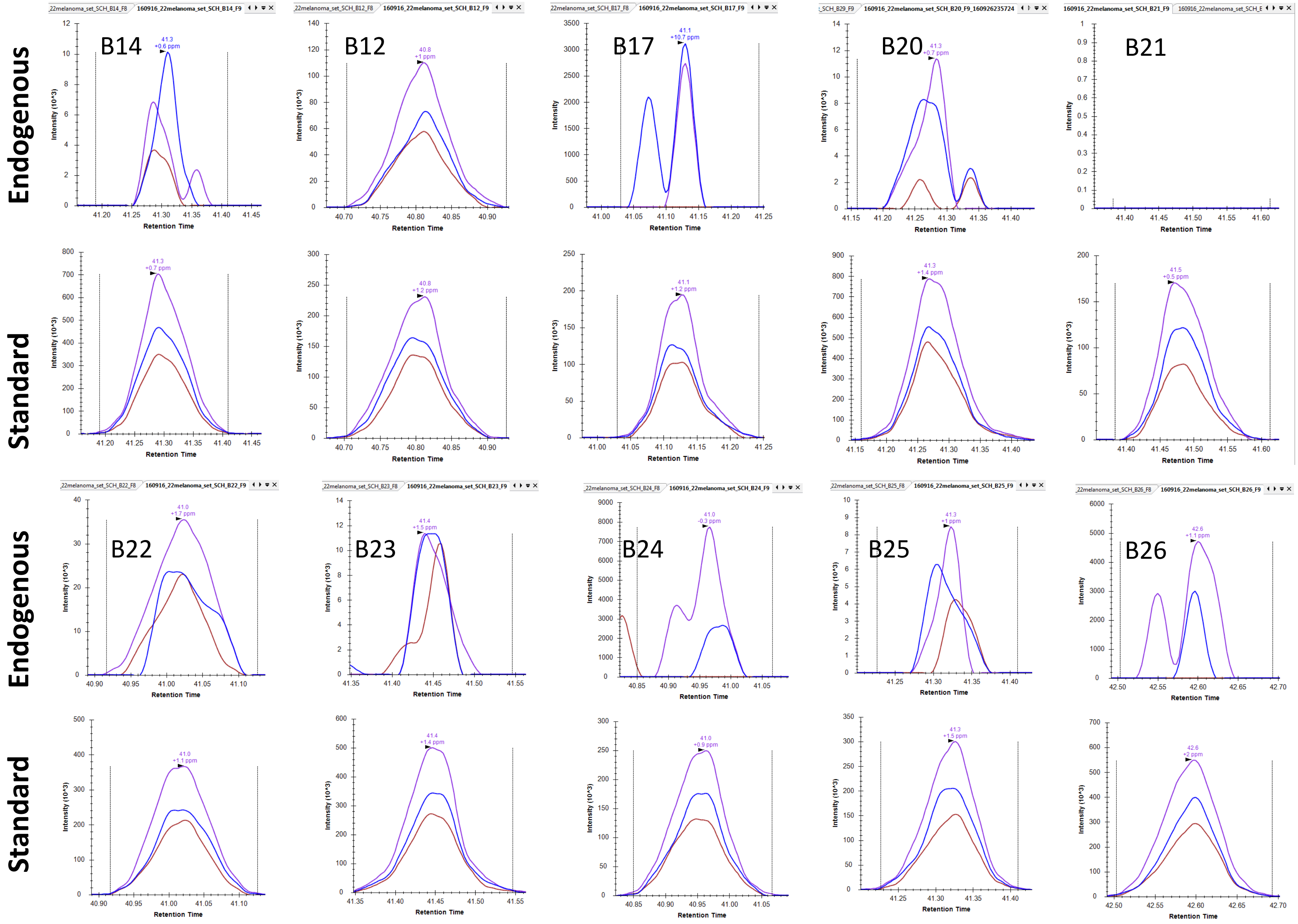

PDL2 endogenous= TPEGLYQVTSVLR **standard** = TPEGLYQVTSVL**R** (C^{13} N^{15})

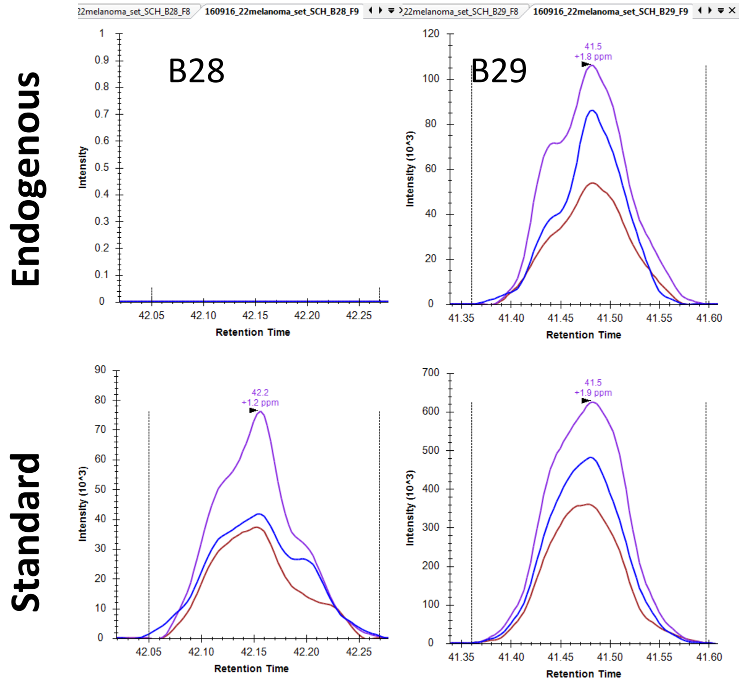

Part II. Relative quantitation of IDO1, LAG3, HAVCR2 (TIM-3), C10orf54 (VISTA) and CD40 by the labeled reference peptide (LRP) method

1. **IDO1** YILIPASQQPK; **reference standard** CD4-ILGNQGSFLT**K** (C^{13} N^{15})
2. **LAG3** LPAGVGTR; **reference standard** PDL1-LQDAGVY**R** (C^{13} N^{15})
3. **HAVCR2 (TIM-3)** LANDLR; **reference standard** PDL1-VNAPYNK (C^{13} N^{15})
4. **C10orf54 (VISTA)** HPLSYVAQR; **reference standard** CD3G- WNLGSNA**K** (C^{13} N^{15})
5. **CD40** YCDPNLGLR; **reference standard** PD1-LAAFPED**R** (C^{13} N^{15})

IDO1

YILIPASQQPK

A representative PRM transitions trace for the peptide YILIPASQQPK is shown at left, along with that for a synthetic standard (unlabeled) YILIPASQQPK peptide.

For quantitation by the LRP method the summed transitions for YILIPASQQPK were normalized to the summed transitions for the CD4 peptide ILGNQGSFLTK (13C15N), which eluted in the same bRPLC fraction. See traces for each sample on the following 3 pages.

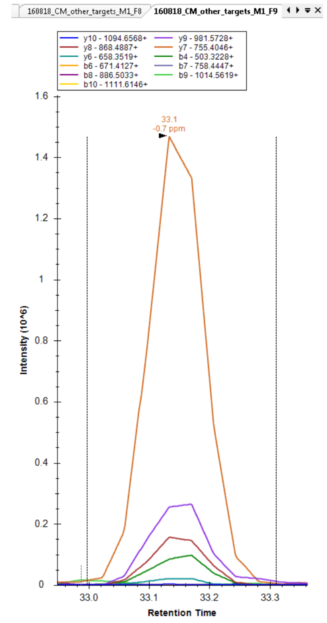

melanoma endogenous

YILIPASQQPK

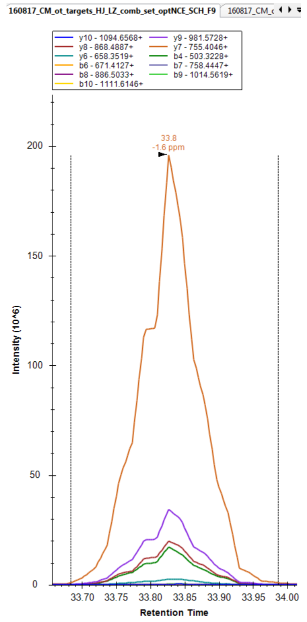

synthetic standard

YILIPASQQPK

IDO1 endogenous= YILIPASQQPK **reference standard** = CD4-ILGNQGSFLT**K** (C^{13} N^{15})

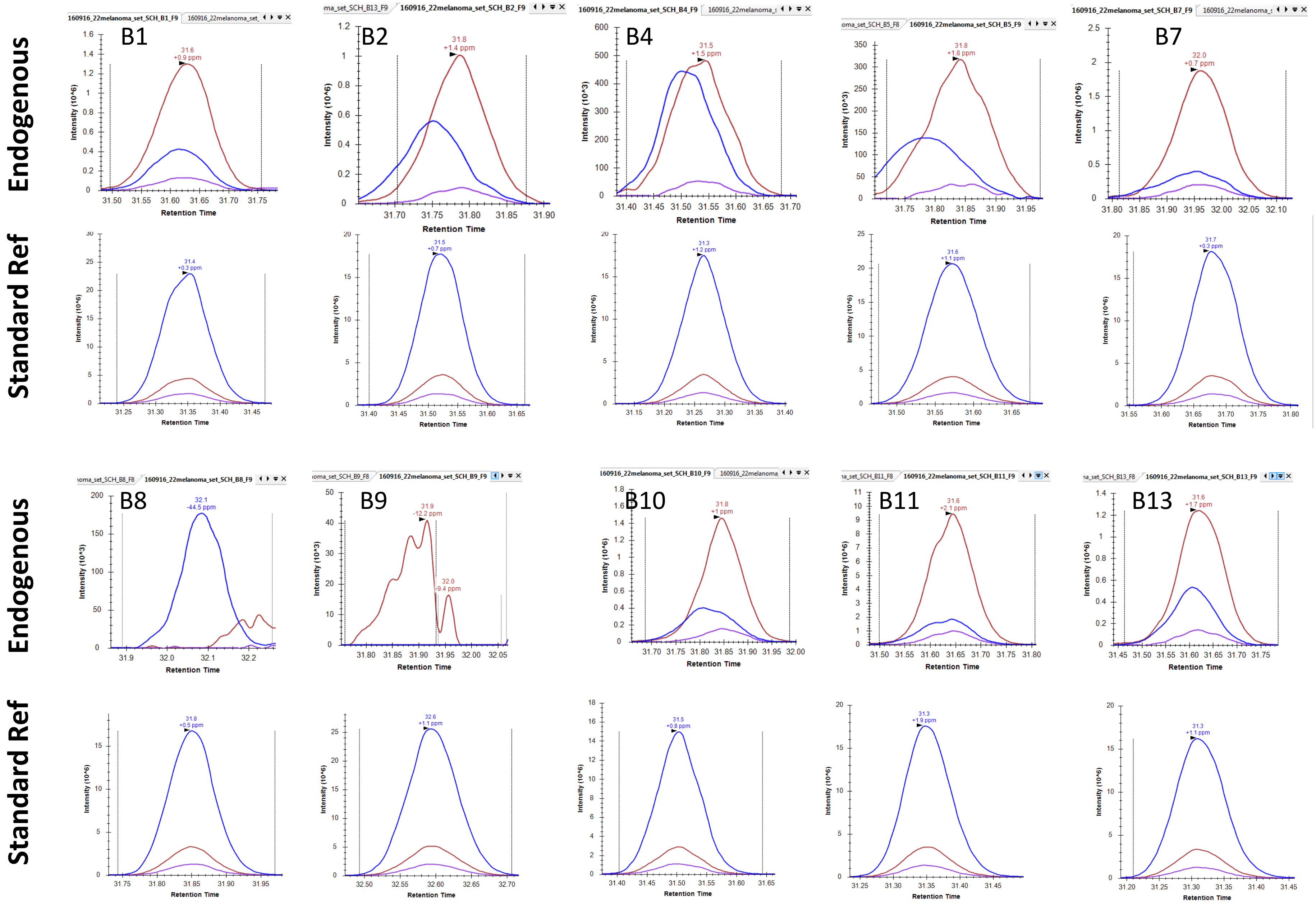

IDO1 endogenous= YILIPASQQPK **reference standard** = CD4-ILGNQGSFLT**K** (C^{13} N^{15})

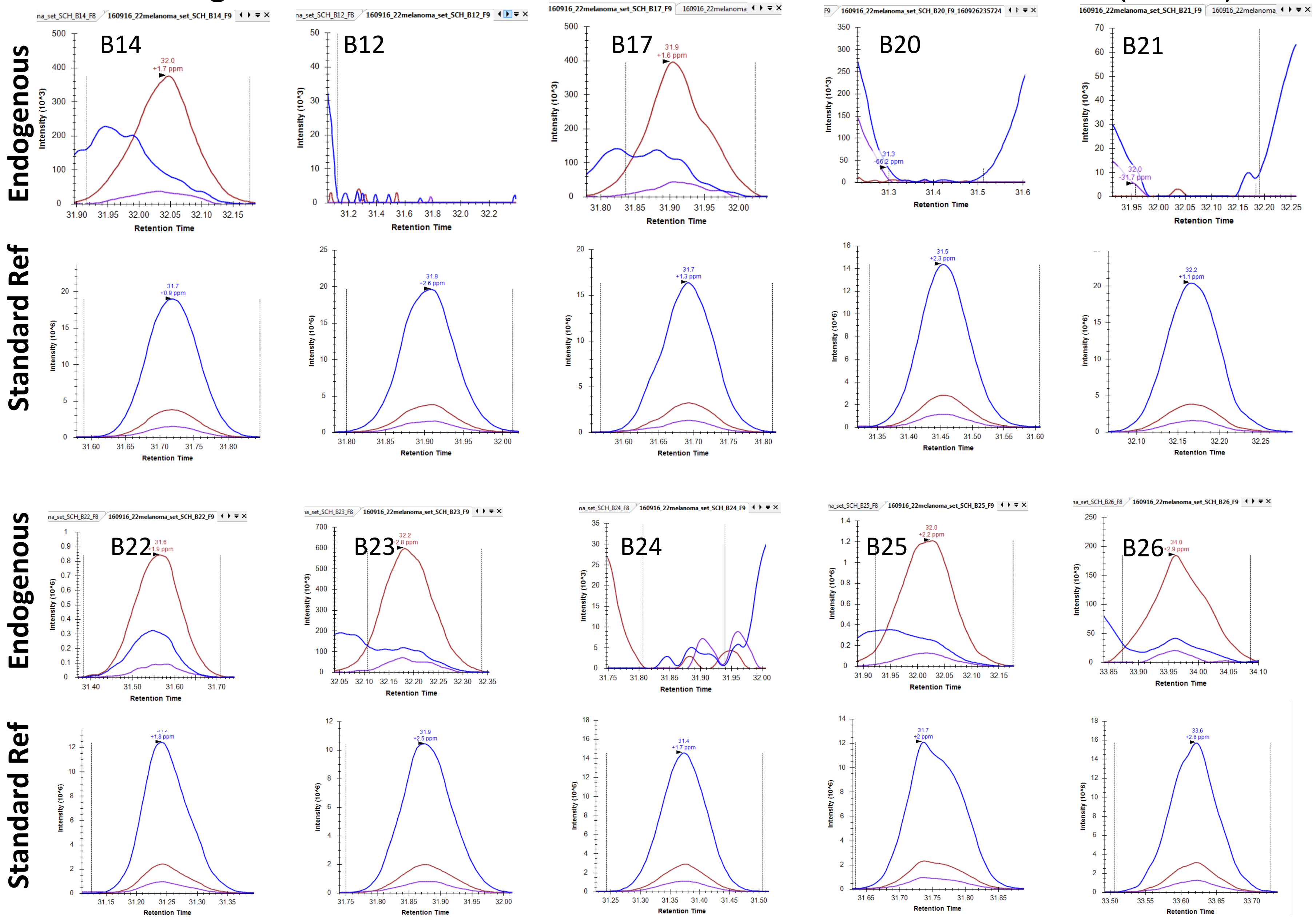

IDO1 endogenous= YILIPASQQPK **reference standard** = CD4-ILGNQGSFLT**K** (C^{13} N^{15})

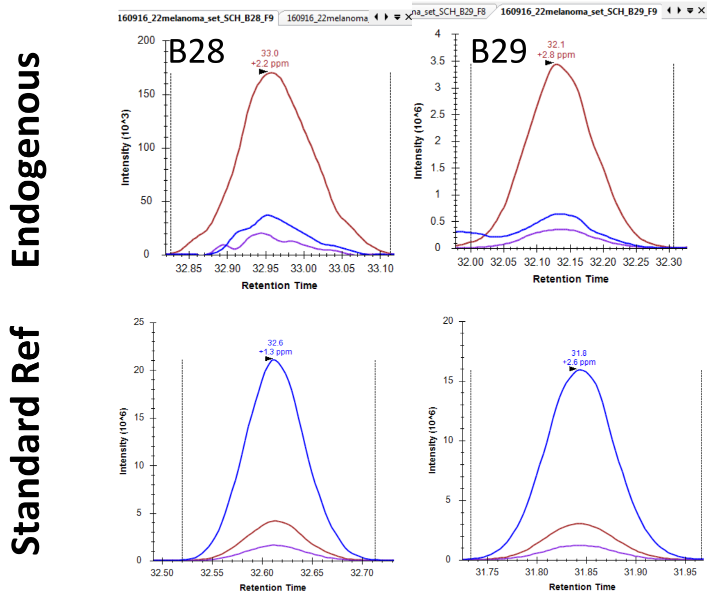

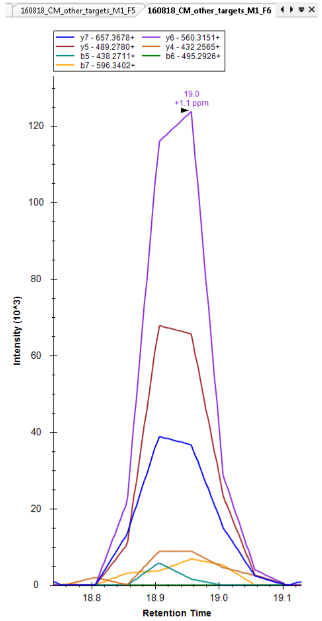

melanoma endogenous

LPAGVGTR

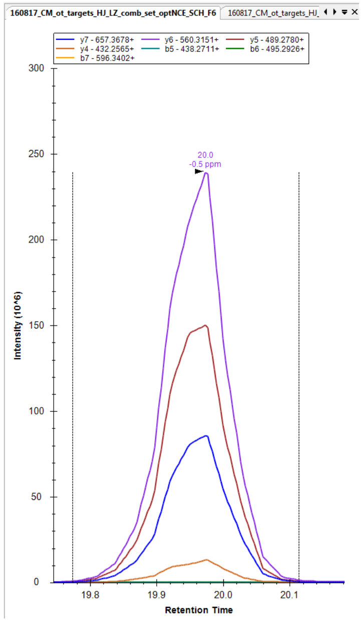

synthetic standard

LPAGVGTR

LAG3

LPAGVGTR

A representative PRM transitions trace for the peptide LPAGVGTR is shown at left, along with that for a synthetic standard (unlabeled) LPAGVGTR peptide.

For quantitation by the LRP method the summed transitions for LPAGVGTR were normalized to the summed transitions for the PD-L1 peptide LQDAGVYR (13C15N), which eluted in the same bRPLC fraction. See traces for each sample on the following 3 pages.

LAG3 endogenous= LPAGVGTR **reference standard** = PDL1-LQDAGVY**R** (C^{13} N^{15})

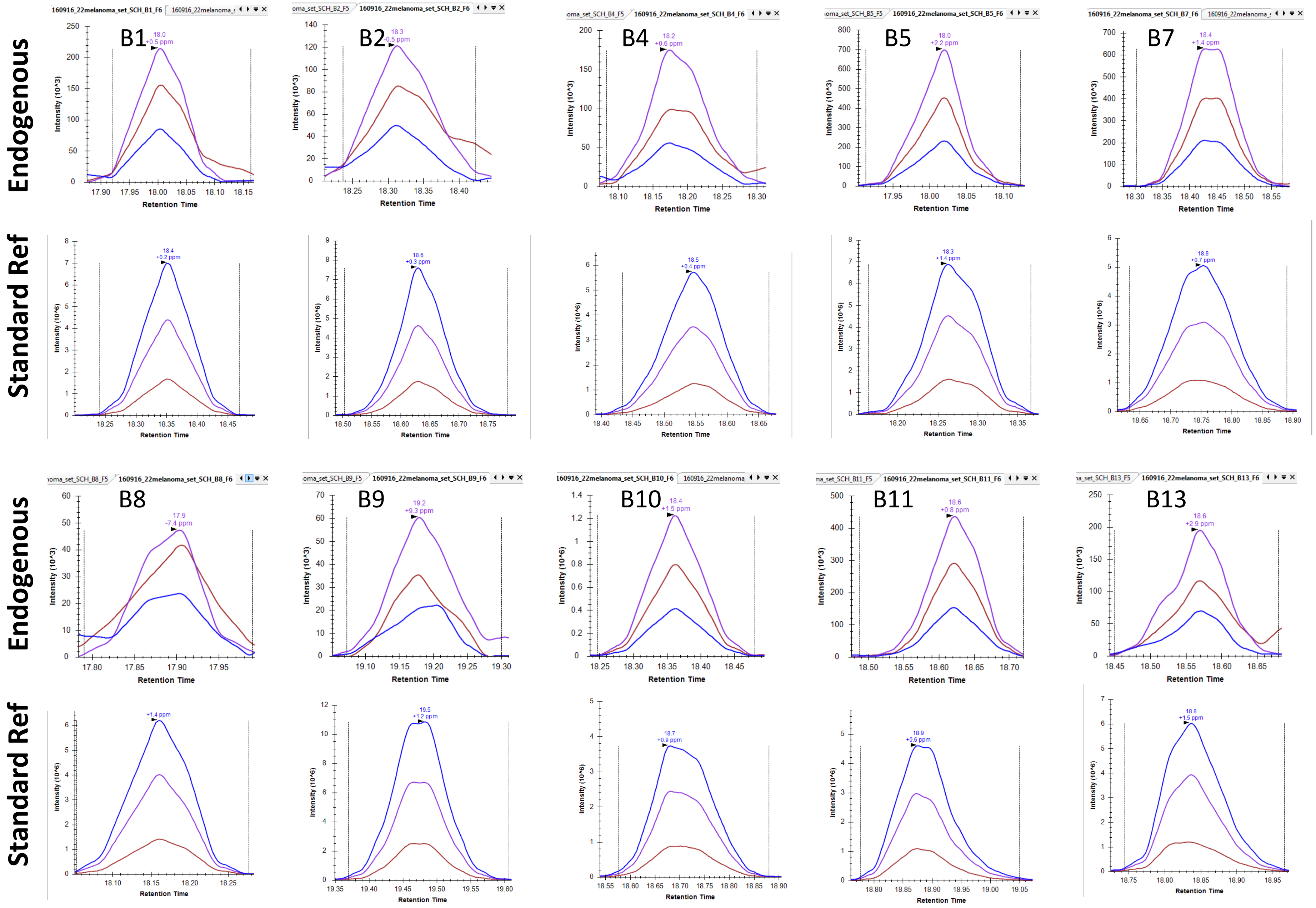

LAG3 endogenous= LPAGVGTR **reference standard** = PDL1-LQDAGVY**R** (C^{13} N^{15})

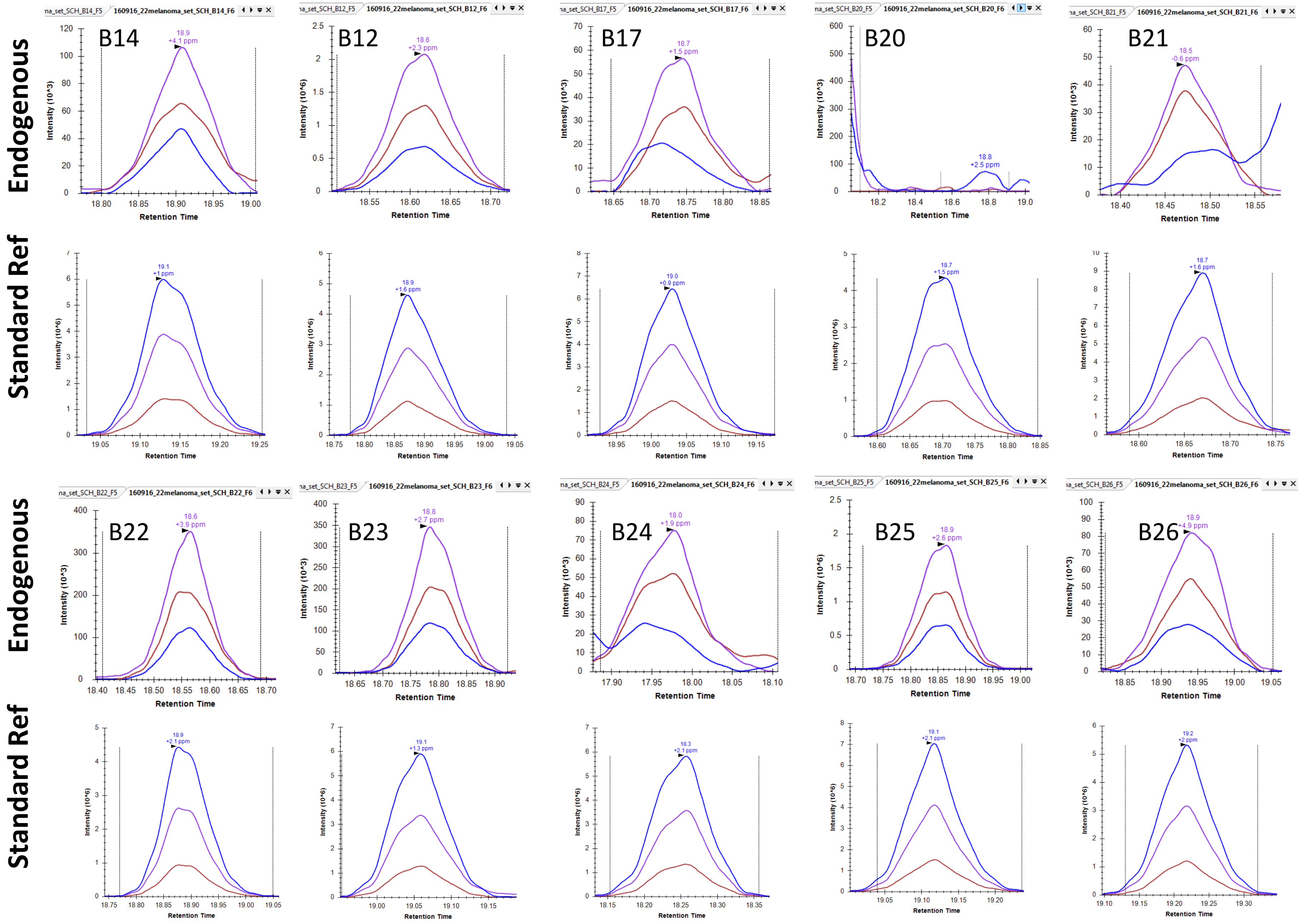

LAG3 endogenous= LPAGVGTR **reference standard** = PDL1-LQDAGVY**R** (C^{13} N^{15})

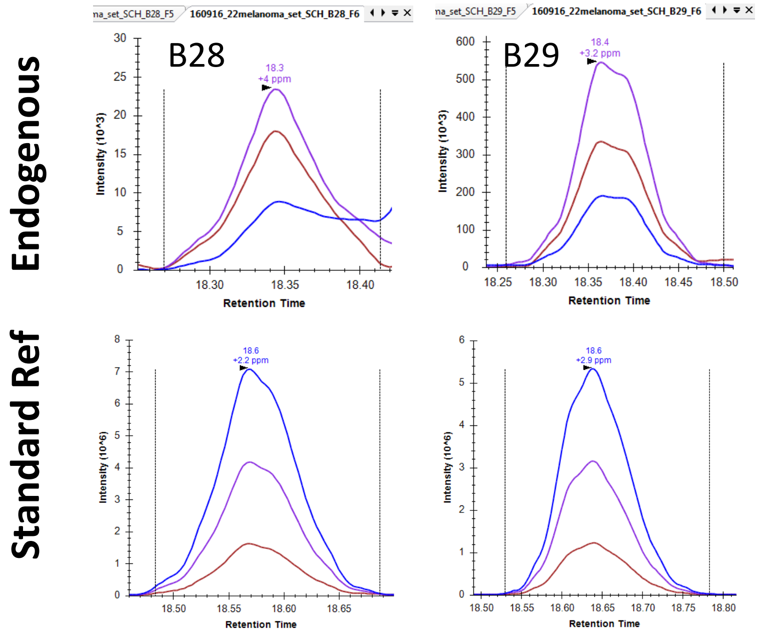

HAVCR2 (TIM-3)

LANDLR

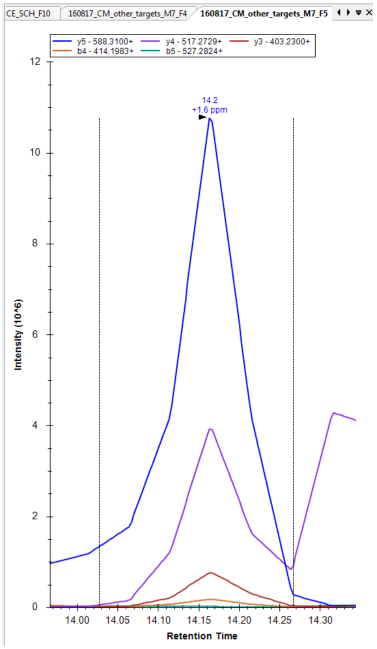

melanoma endogenous

LANDLR

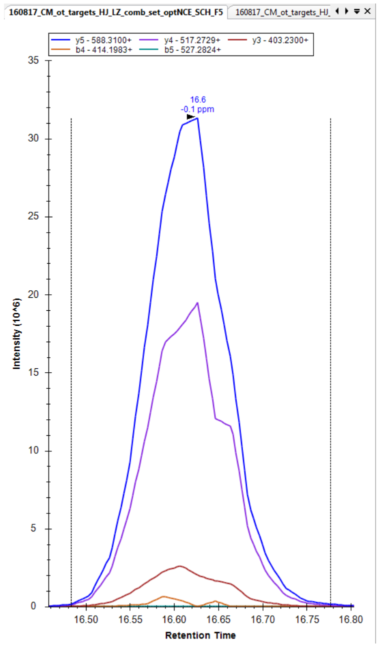

synthetic standard

LANDLR

A representative PRM transitions trace for the peptide LANDLR is shown at left, along with that for a synthetic standard (unlabeled) LANDLR peptide.

For quantitation by the LRP method the summed transitions for LANDLR were normalized to the summed transitions for the PD-L1 peptide VNAPYNK (13C15N), which eluted in the same bRPLC fraction. See traces for each sample on the following 3 pages.

HAVCR2 (TIM-3) **endogenous**= LANDLR **reference standard** = PDL1-VNAPYN**K** (C^{13} N^{15})

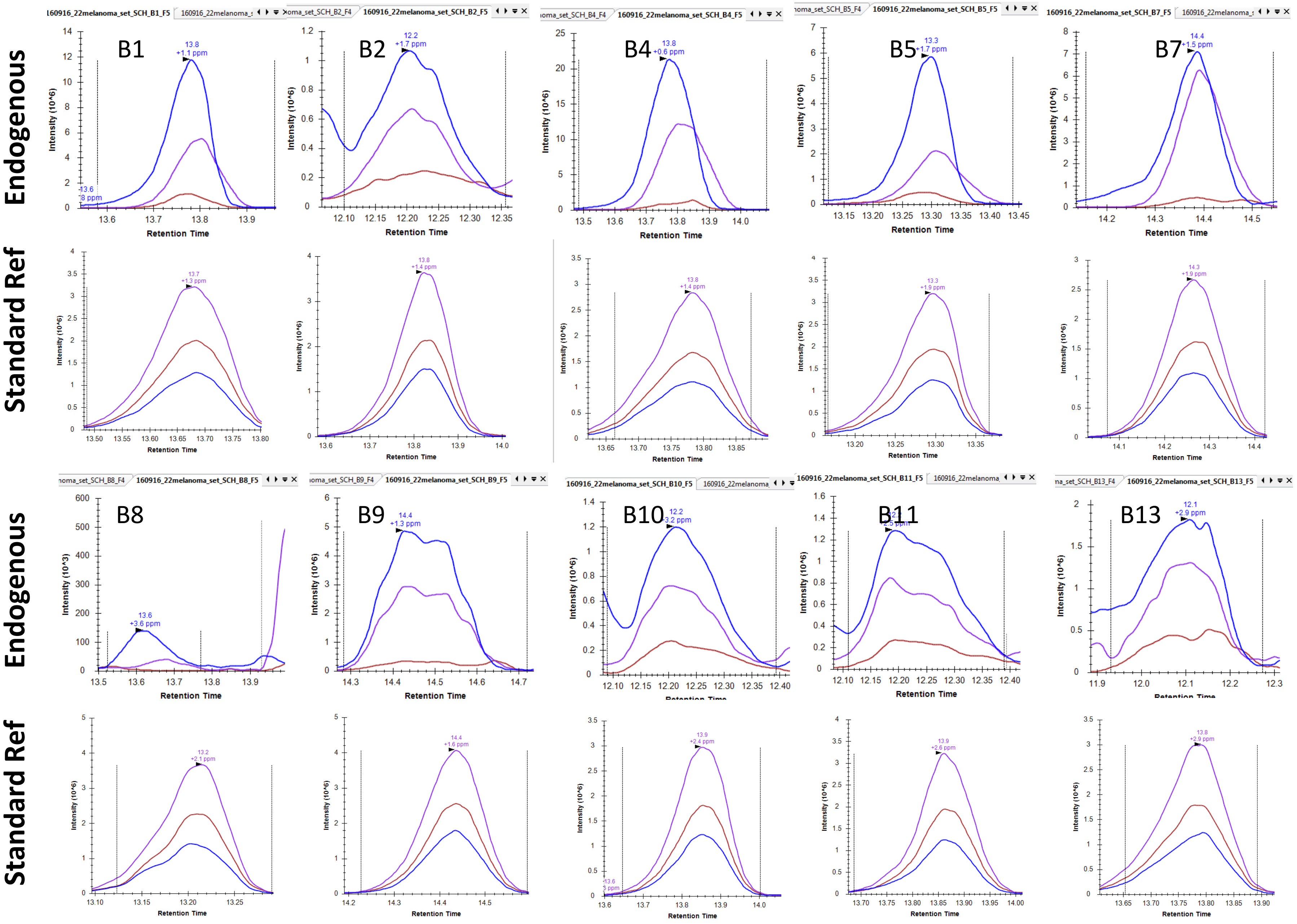

HAVCR2 (TIM-3) endogenous= LANDLR reference standard = PDL1-VNAPYNK (C^{13} N^{15})

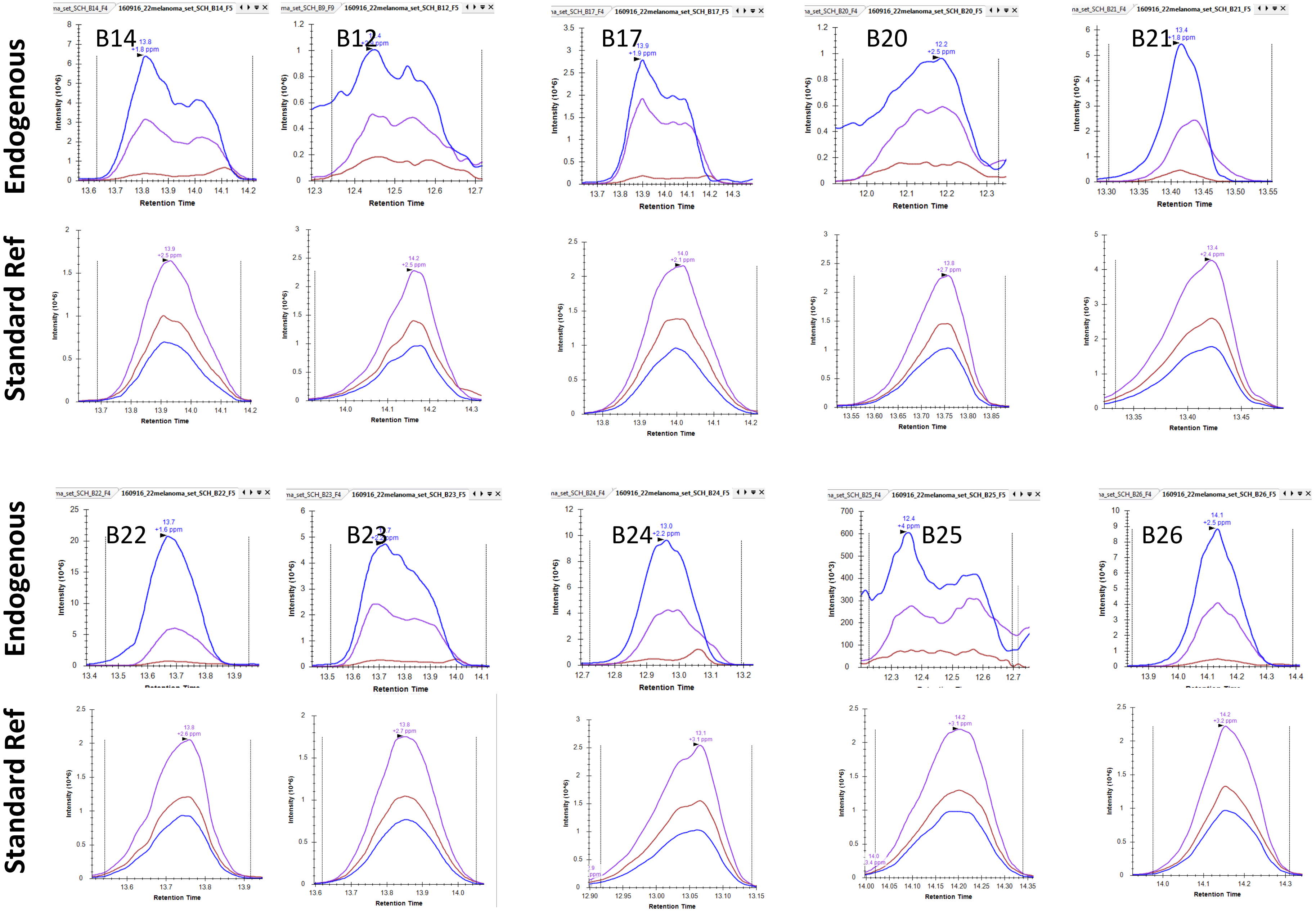

HAVCR2 (TIM-3) endogenous= LANDLR **reference standard** = PDL1-VNAPYN**K** (C^{13} N^{15})

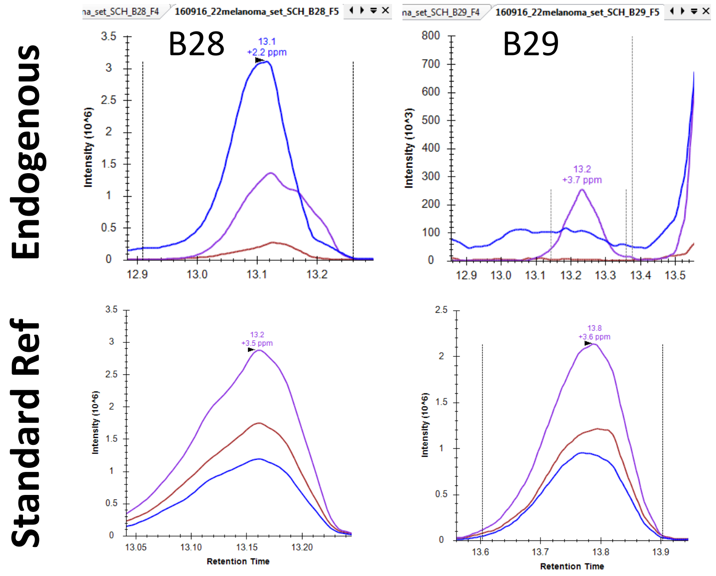

C10orf54 (VISTA)

HPLSYVAQR

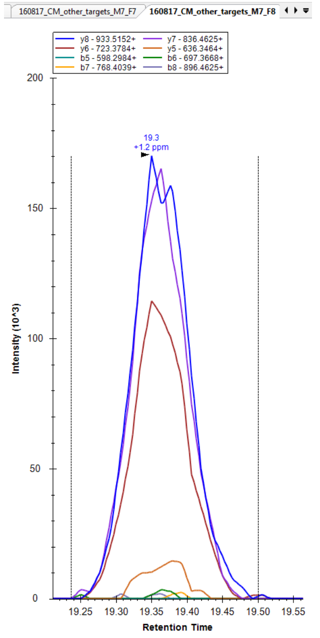

melanoma endogenous

HPLSYVAQR

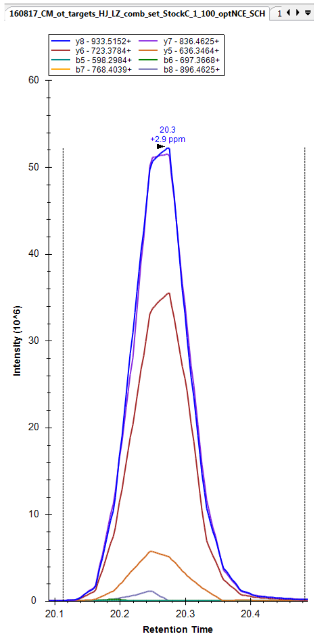

synthetic standard

HPLSYVAQR

A representative PRM transitions trace for the peptide HPLSYVAQR is shown at left, along with that for a synthetic standard (unlabeled) HPLSYVAQR peptide.

For quantitation by the LRP method the summed transitions for HPLSYVAQR were normalized to the summed transitions for the CD3G peptide WNLGSNAK (13C15N), which eluted in the same bRPLC fraction. See traces for each sample on the following 3 pages.

C10orf54 (VISTA) = HPLSYVAQR **reference standard** = CD3G- WNLGSNA**K** (C^{13} N^{15})

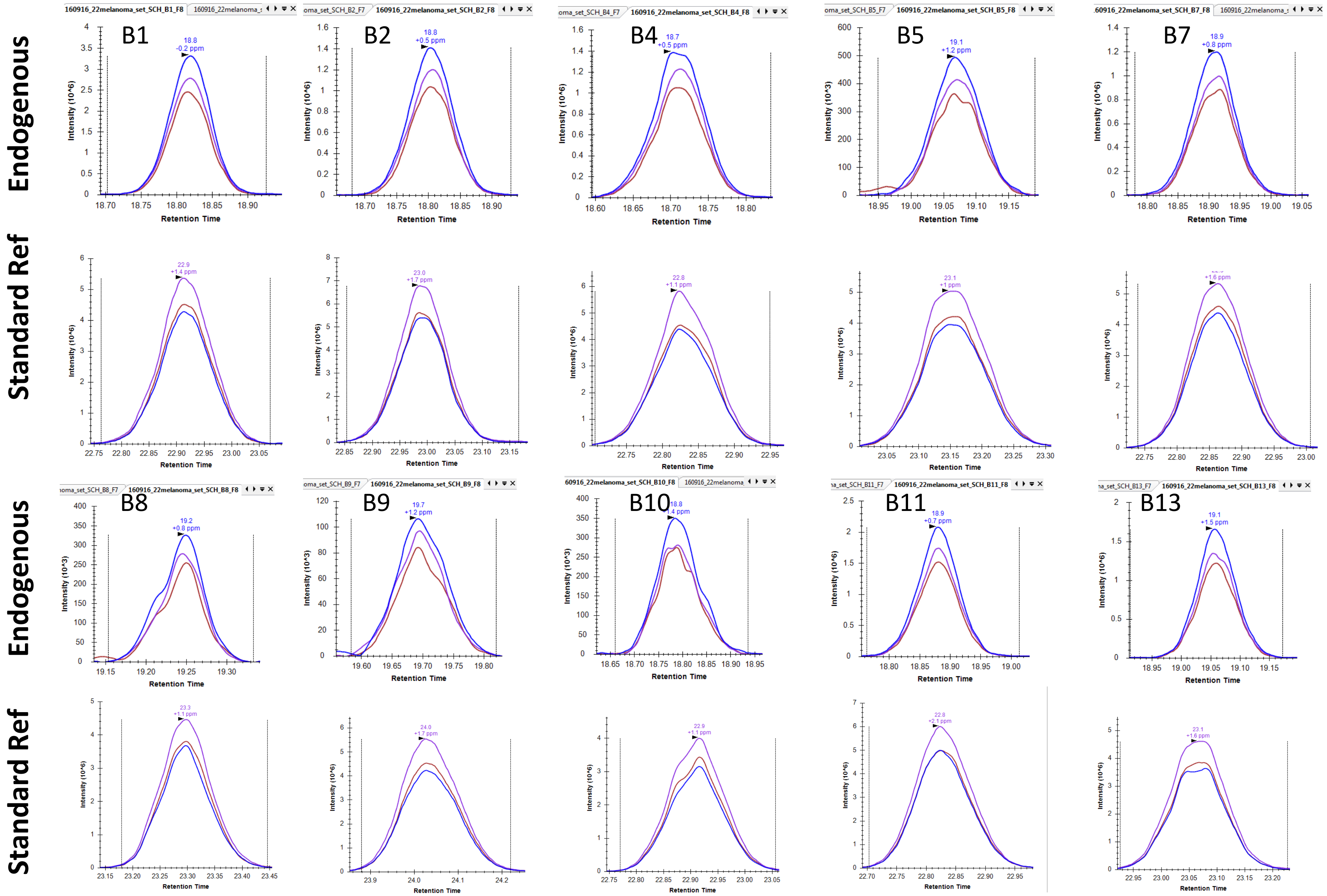

C10orf54 (VISTA) = HPLSYVAQR **reference standard** = CD3G- WNLGSNA**K** (C^{13} N^{15})

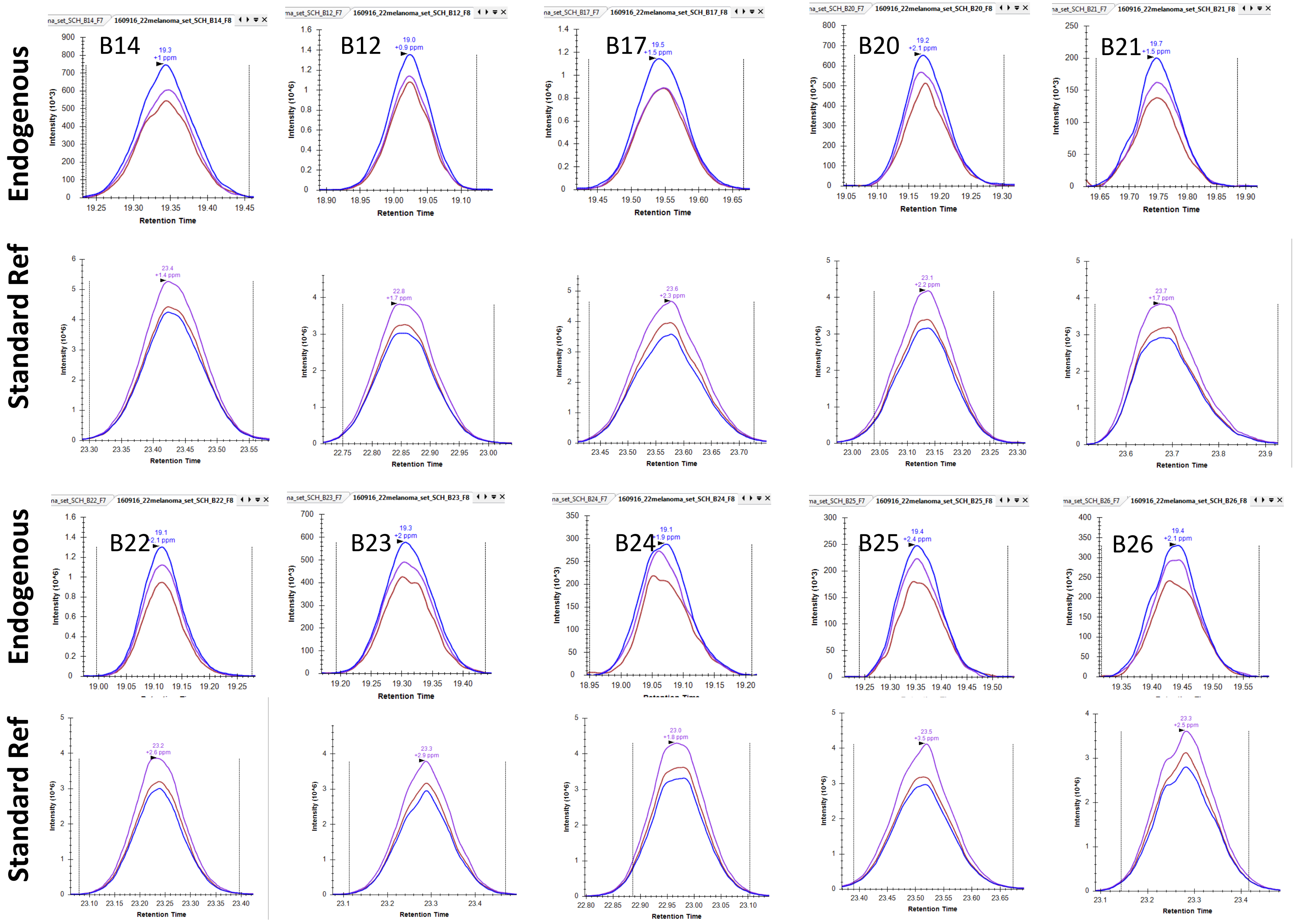

C10orf54 (VISTA) = HPLSYVAQR **reference standard** = CD3G- WNLGSNA**K** (C^{13} N^{15})

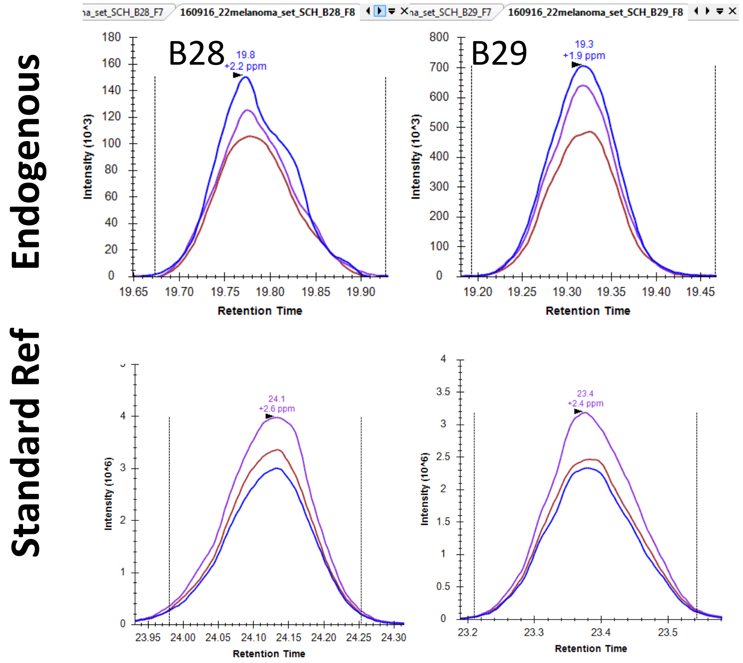

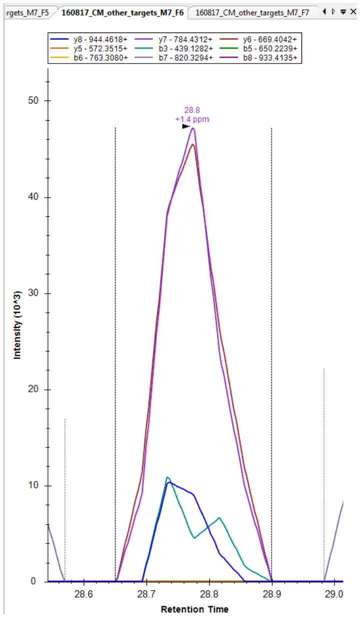

melanoma endogenous

YC[+57]DPNLGLR

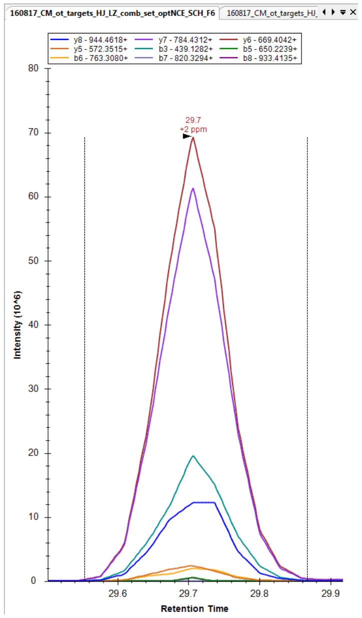

synthetic standard

YC[+57]DPNLGLR

CD40

YC[+57]DPNLGLR

A representative PRM transitions trace for the peptide YC[+57]DPNLGLR is shown at left, along with that for a synthetic standard (unlabeled) YC[+57]DPNLGLR peptide.

For quantitation by the LRP method the summed transitions for YC[+57]DPNLGLR were normalized to the summed transitions for the PD-1 peptide LAAFPEDR (13C15N), which eluted in the same bRPLC fraction. See traces for each sample on the following 3 pages.

CD40 endogenous= YC[+57]DPNLGLR **reference standard** = PD1-LAAFPED**R** (C^{13} N^{15})

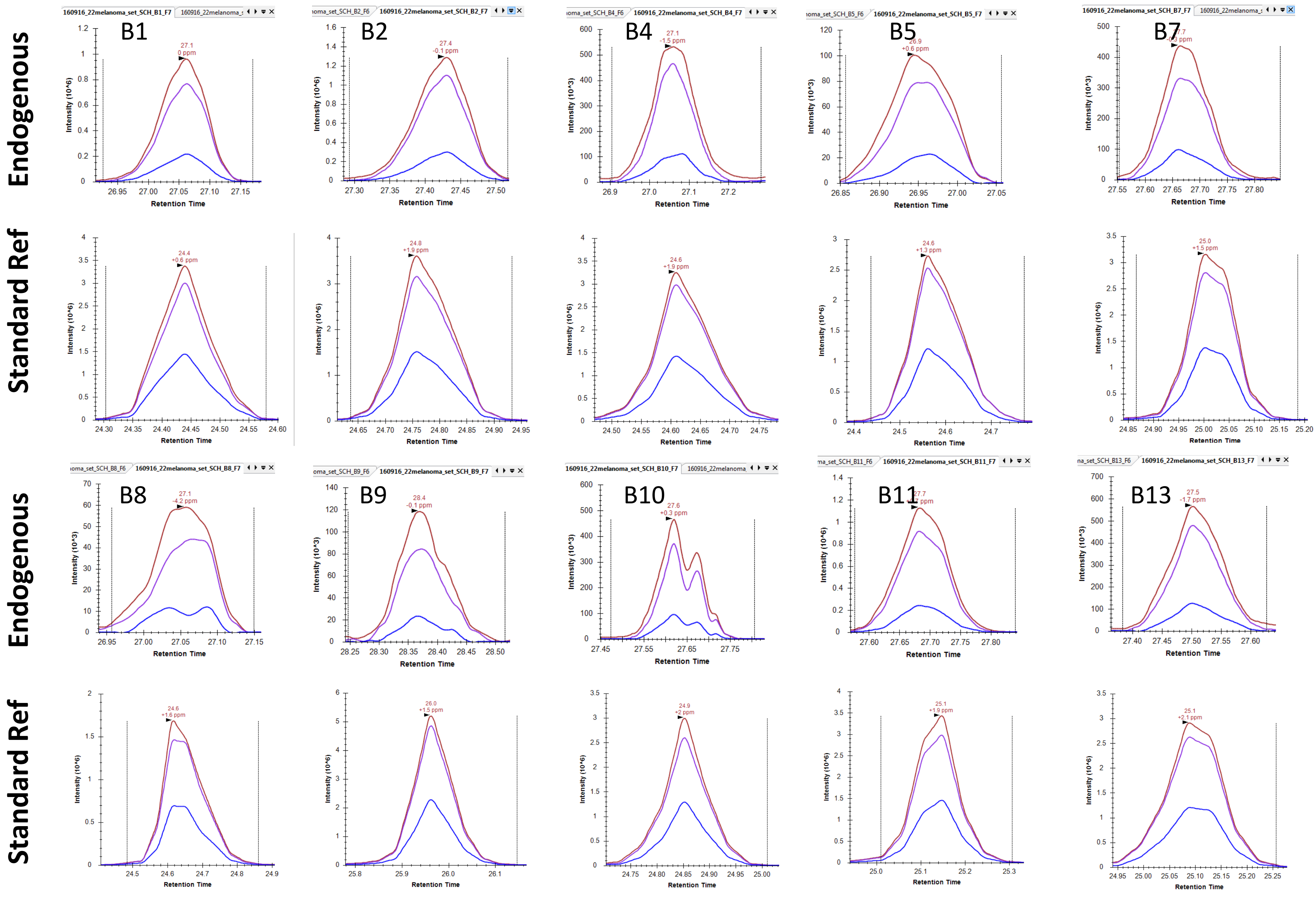

CD40 endogenous= YC[+57]DPNLGLR **reference standard** = PD1-LAAFPED**R** (C^{13} N^{15})

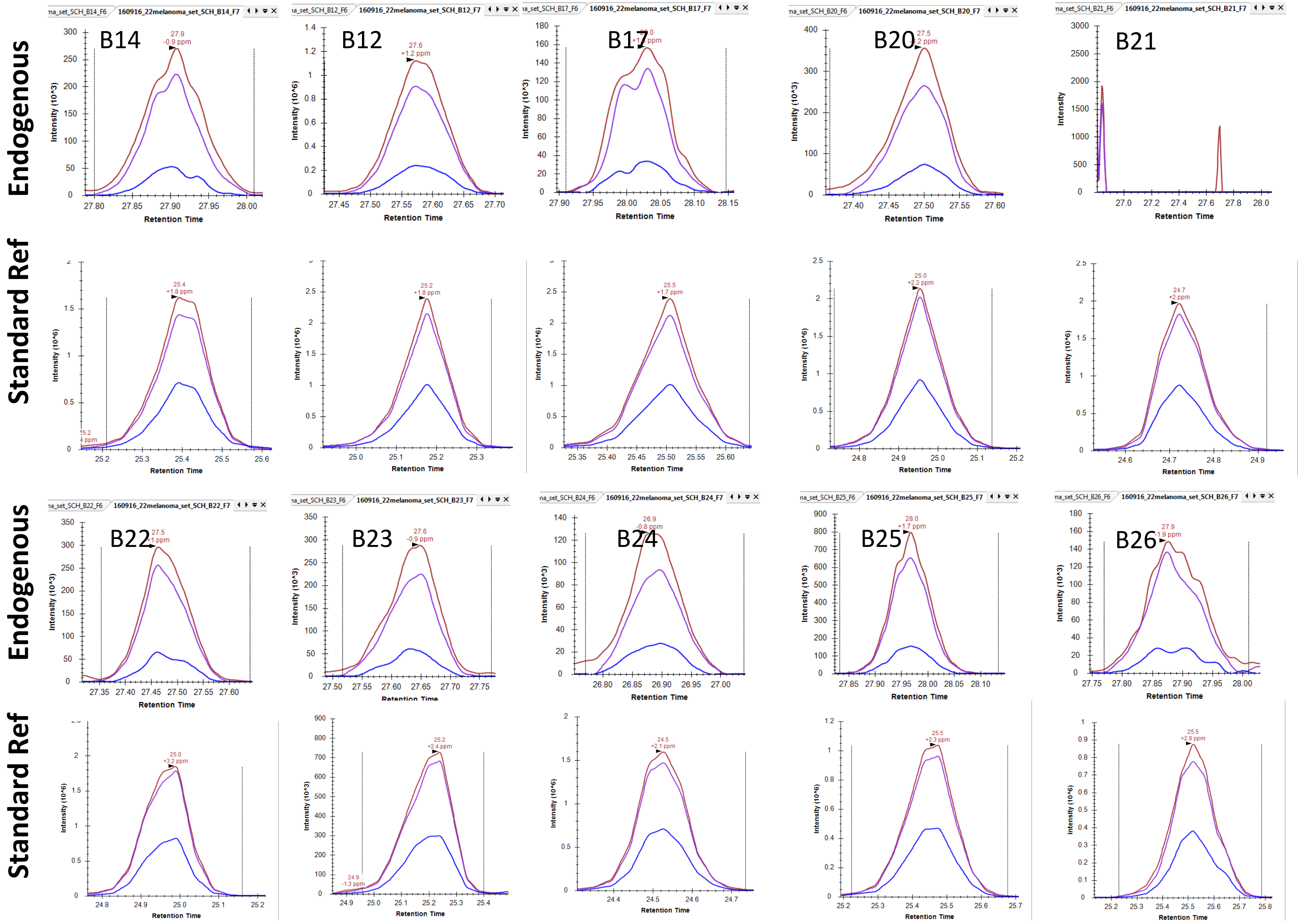

CD40 endogenous= YC[+57]DPNLGLR **reference standard** = PD1-LAAFPED**R** (C^{13} N^{15})

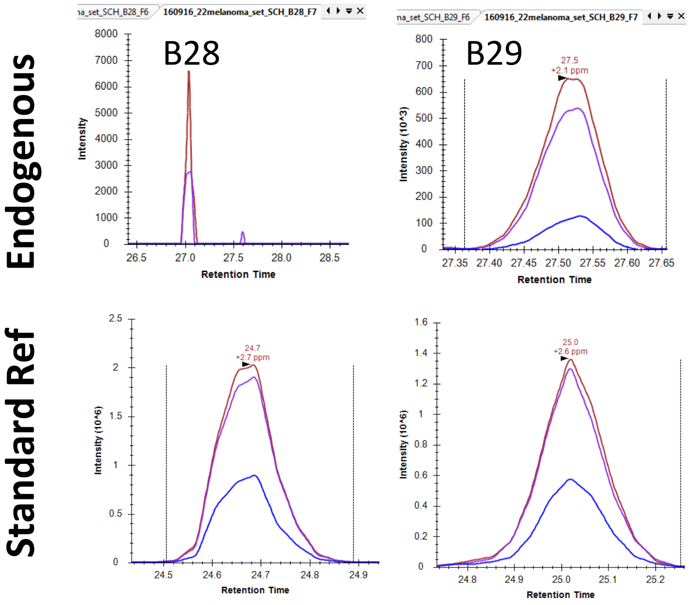

Supplement: Supplemental Data [file supp_RA117.000037_4581_1_supp_1265_cq7ccf.pdf]
