# Supplementary material for: Quantitative Mass Spectrometry Analysis of PD-L1 Protein Expression, N-glycosylation and Expression Stoichiometry with PD-1 and PD-L2 in Human Melanoma
Source: Mol Cell Proteomics. 2017 May 25;16(10):1705–17. doi: 10.1074/mcp.RA117.000037 (PMC5629259; doi:10.1074/mcp.RA117.000037)
Supplement: Supplemental Data [file supp_RA117.000037_4581_1_supp_1266_nq7nng.pdf]

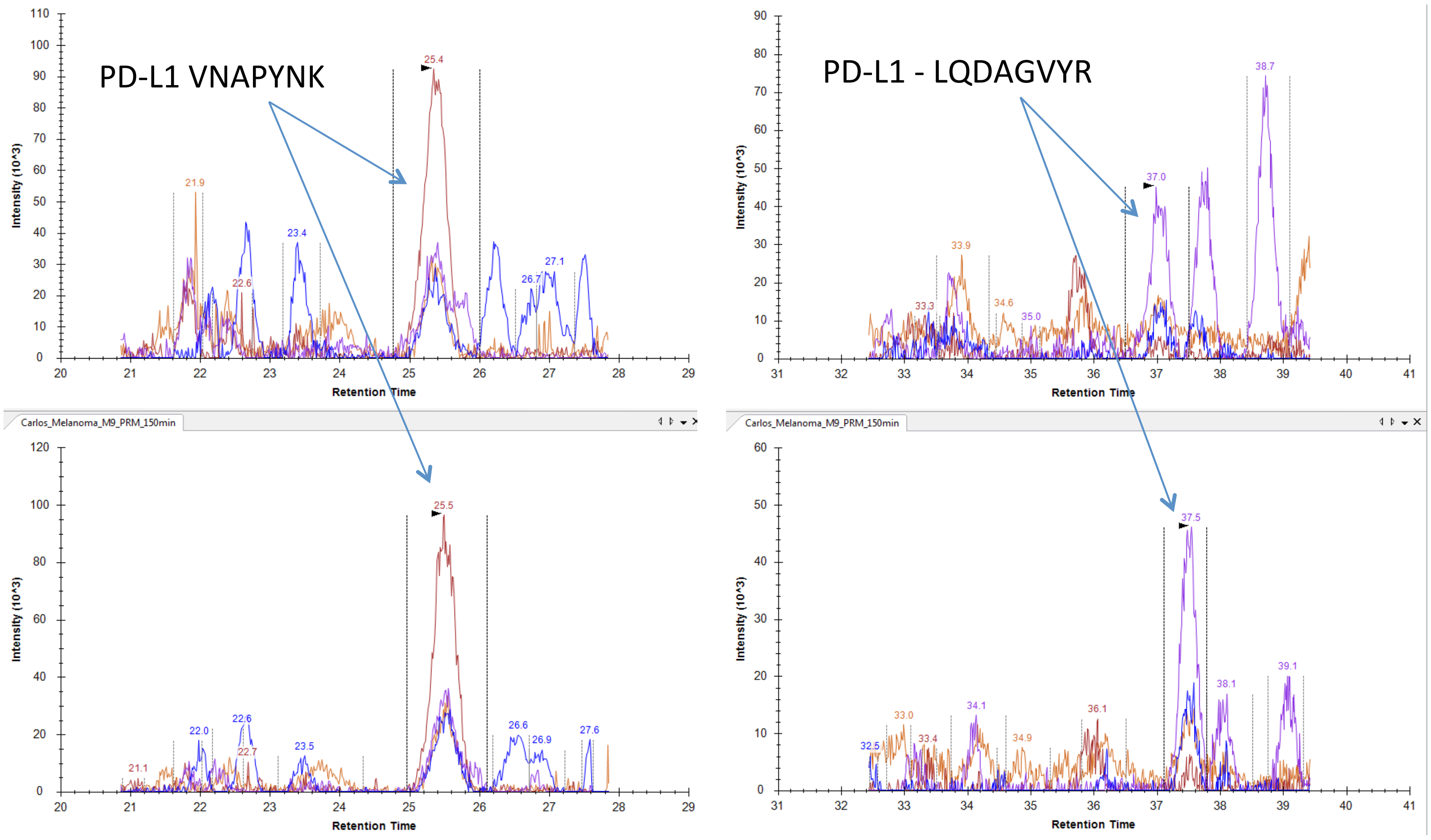

Figure S1. Detection of PD-L1 peptides in long gradient LC-PRM analyses of unfractionated digests from FFPE melanomas.

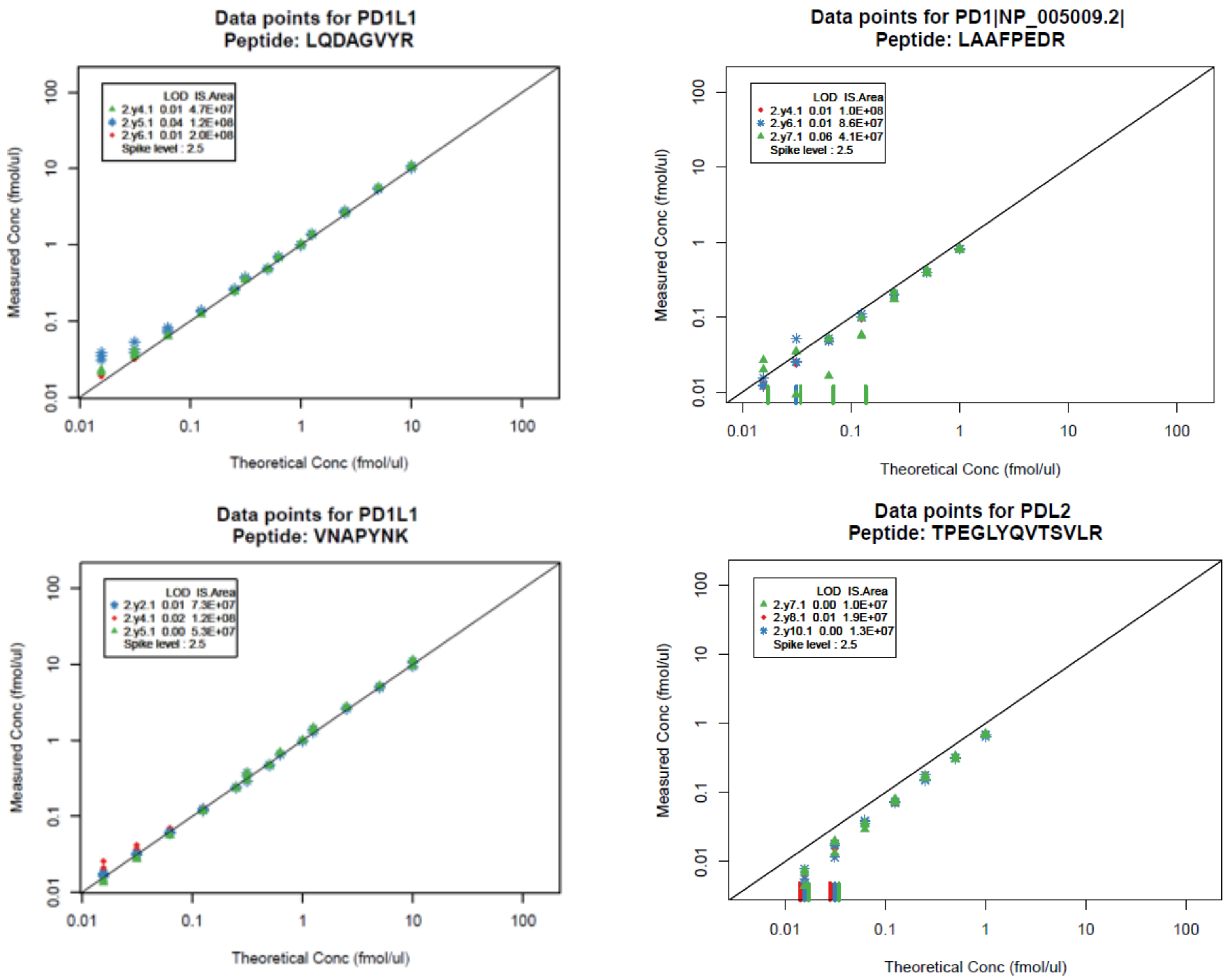

Figure S2. SID calibration curves

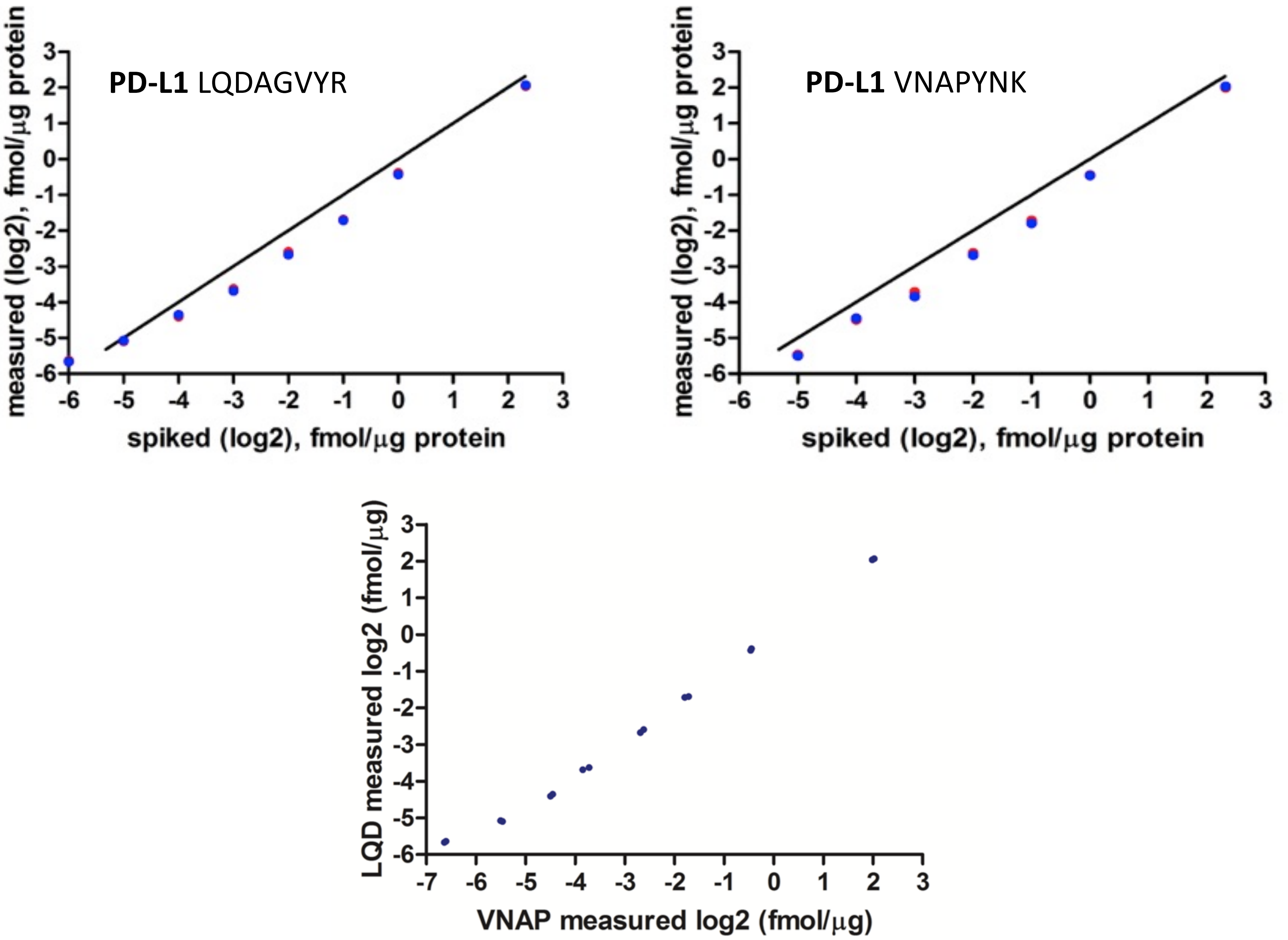

Figure S3. Spike recovery study for recombinant human PD-L1 100 μg HEK293 cell lysate 0.031, 0.063, 0.125, 0.25, 0.5, 1 or 5 fmol/microgram protein.

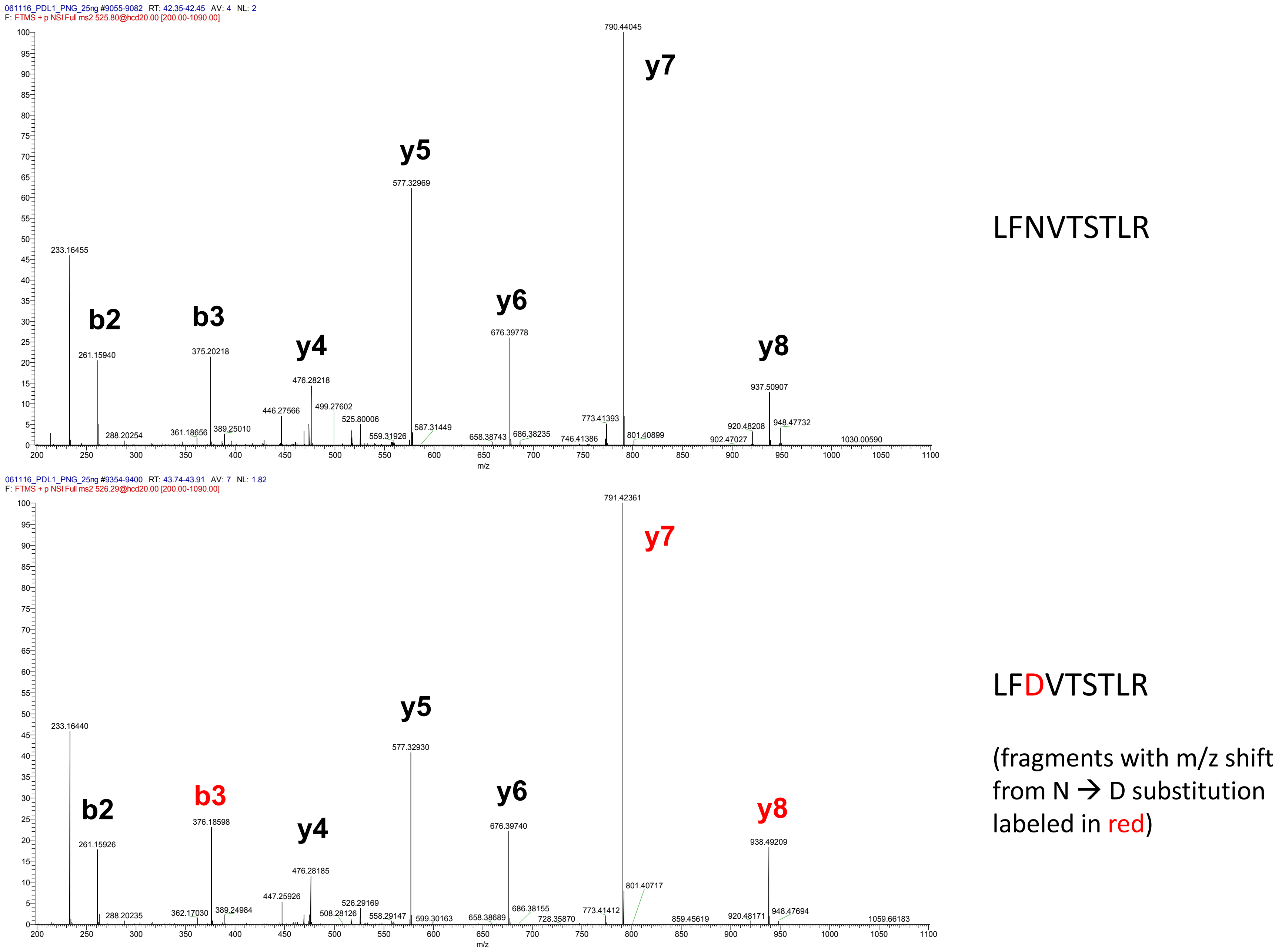

Figure S4. MS/MS spectra of PD-L1 LFNVTSTLR peptide and LFDVTSTLR peptide released by PNGase F treatment of recombinant human PD-L1 tryptic digest.

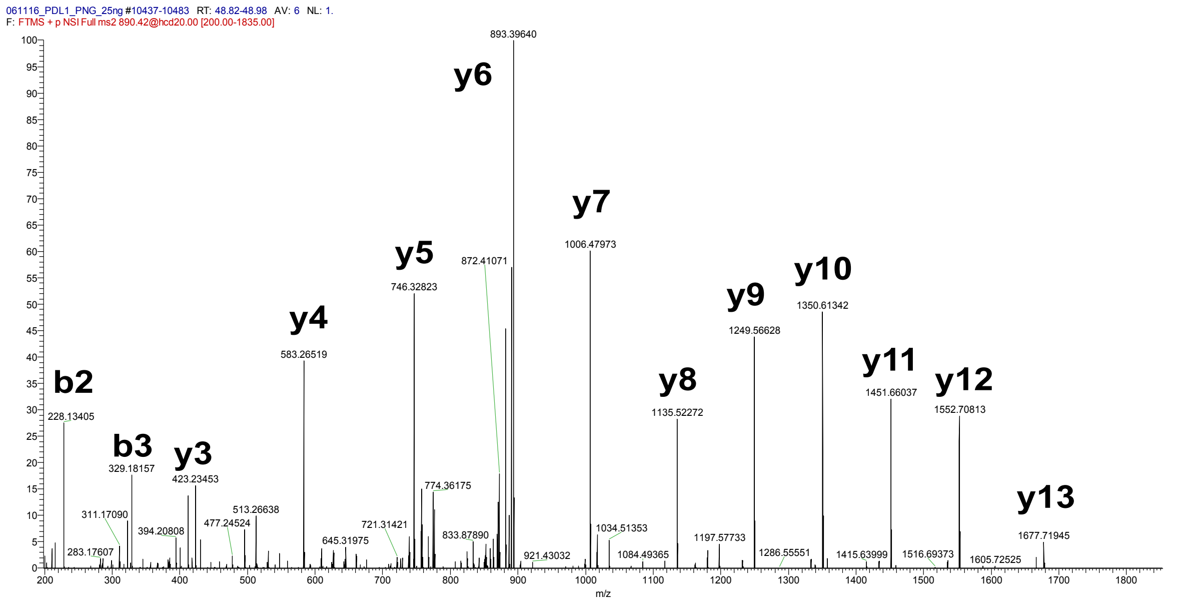

INTTTNEIFYCTFR

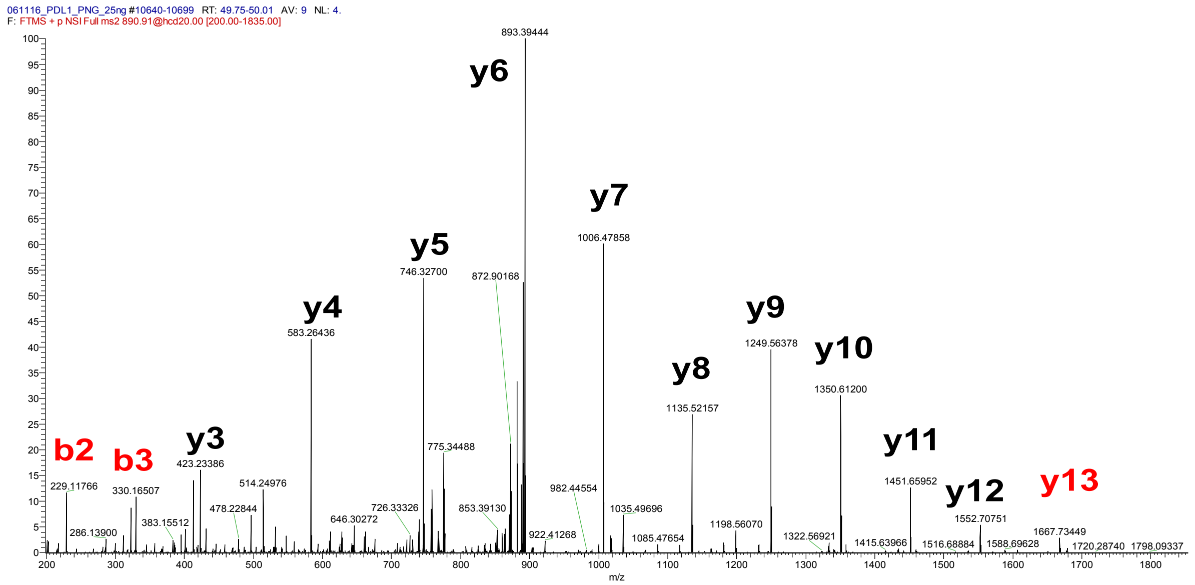

IDTTTNEIFYCTFR

(fragments with m/z shift from N → D substitution labeled in red)

Figure S5. MS/MS spectra of PD-L1 INTTTNEIFYCTFR peptide and L1 IDTTTNEIFYCTFR peptide released by PNGase F treatment of recombinant human PD-L1 tryptic digest.

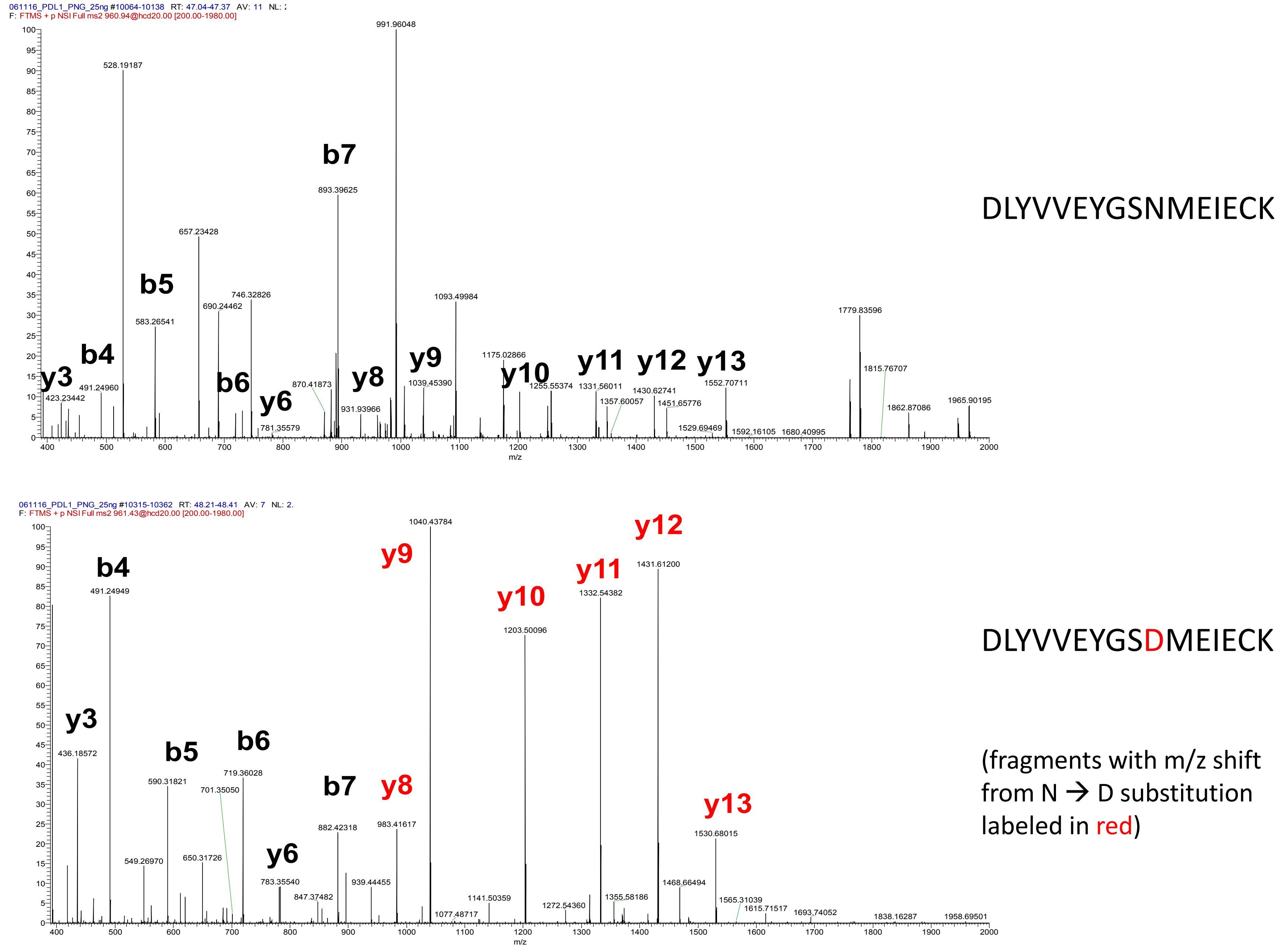

Figure S6. MS/MS spectra of PD-L1 DLYVVEYGSNMEIECK peptide and L1 DLYVVEYGSDMEIECK peptide released by PNGase F treatment of recombinant human PD-L1 tryptic digest.

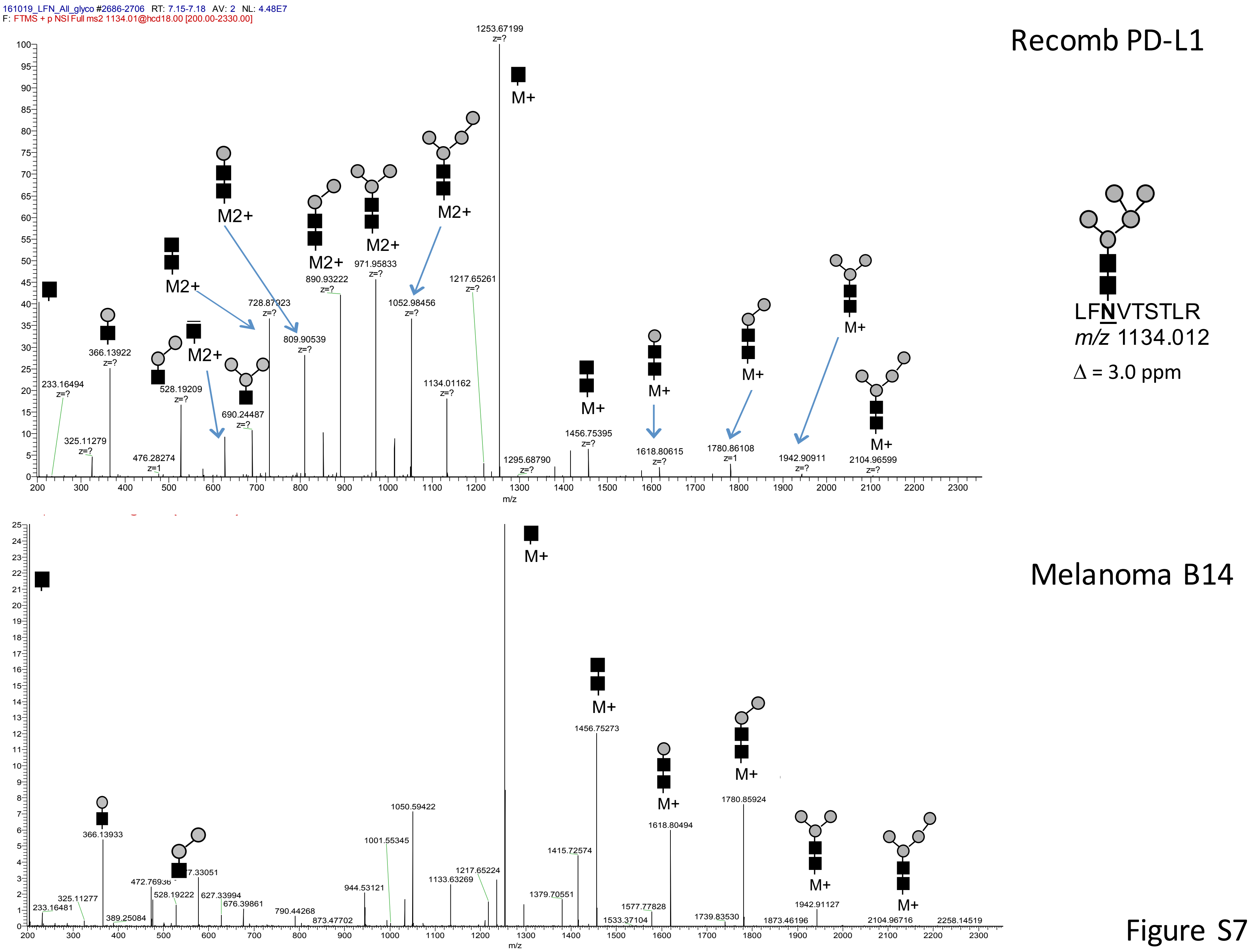
161019_LFN_All_glyco #2686-2706 RT: 7.15-7.18 AV: 2 NL: 4.48E7
F: FTMS + p NSI Full ms2 1134.01@hcd18.00 [200.00-2330.00]
Recomb PD-L1
LFNVTSTLR
m/z 1134.012
Δ = 3.0 ppm
1253.67199
M+
M2+
233.16494
325.11279
366.13922
476.28274
528.19209
690.24487
728.87923
809.90539
890.93222
971.95833
1052.98456
1134.01162
1217.65261
1295.68790
1456.75395
1618.80615
1780.86108
1942.90911
2104.96599
m/z
Melanoma B14
233.16481
325.11277
366.13933
389.25084
472.76936
528.19222
627.33994
676.39861
790.44268
873.47702
944.53121
1001.55345
1050.59422
1133.63269
1217.65224
1379.70551
1415.72574
1456.75273
1533.37104
1577.77828
1618.80494
1739.83530
1780.85924
1873.46196
1942.91127
2104.96716
2258.14519
m/z

Figure S7

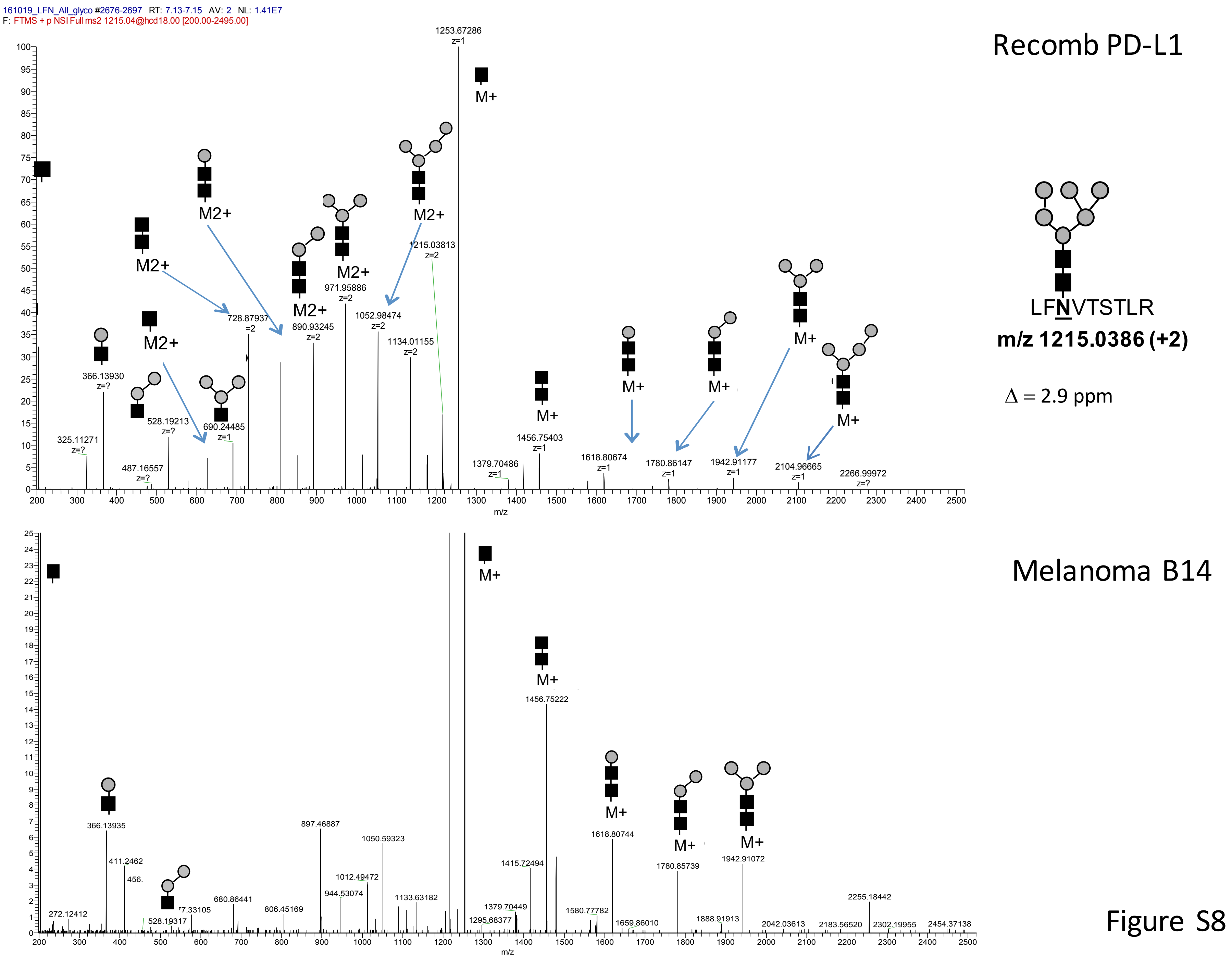

161019_LFN_All_glyco #2676-2697 RT: 7.13-7.15 AV: 2 NL: 1.41E7
F: FTMS + p NSI Full ms2 1215.04@hcd18.00 [200.00-2495.00]
Recomb PD-L1
LFNVTSTLR
m/z 1215.0386 (+2)
Δ = 2.9 ppm
1253.67286 z=1
M+
M2+
1215.03813 z=2
971.95886 z=2
890.93245 z=2
1052.98474 z=2
1134.01155 z=2
728.87937 =2
366.13930 z=?
325.11271 z=?
487.16557 z=?
528.19213 z=?
690.24485 z=1
1379.70486 z=1
1456.75403 z=1
1618.80674 z=1
1780.86147 z=1
1942.91177 z=1
2104.96665 z=1
2266.99972 z=?
m/z
Melanoma B14
1456.75222
1618.80744
1780.85739
1942.91072
366.13935
411.2462
272.12412
528.19317
680.86441
806.45169
897.46887
944.53074
1012.49472
1050.59323
1133.63182
1295.68377
1379.70449
1415.72494
1580.77782
1659.86010
1888.91913
2042.03613
2183.56520
2255.18442
2302.19955
2454.37138

Figure S8

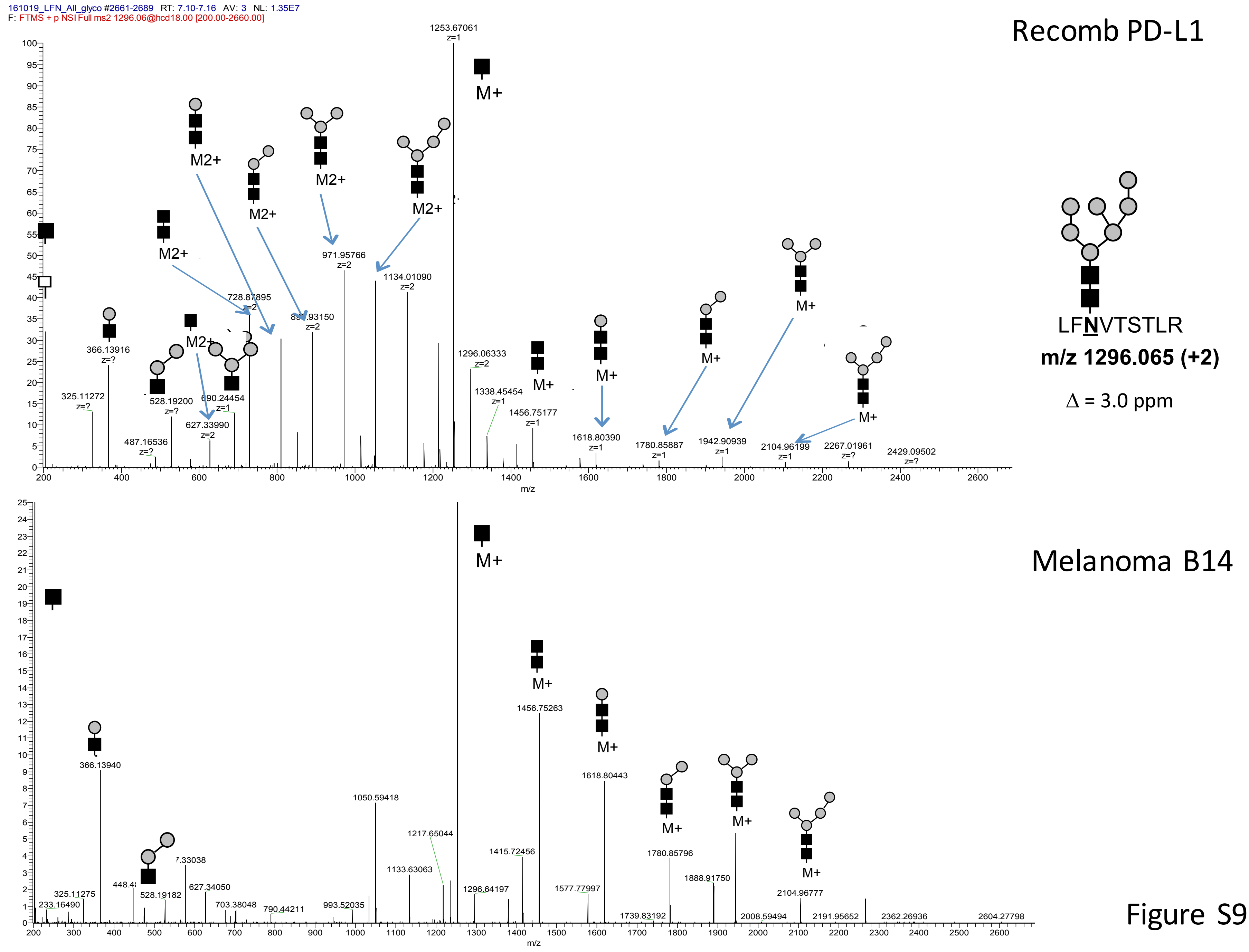
161019_LFN_All_glyco #2661-2689 RT: 7.10-7.16 AV: 3 NL: 1.35E7
F: FTMS + p NSI Full ms2 1296.06@hcd18.00 [200.00-2660.00]
Recomb PD-L1
1253.67061 z=1
M+
M2+
971.95766 z=2
1134.01090 z=2
728.87895 z=2
366.13916 z=?
325.11272 z=?
487.16536 z=?
528.19200 z=?
627.33990 z=2
690.24454 z=1
1296.06333 z=2
1338.45454 z=1
1456.75177 z=1
1618.80390 z=1
1780.85887 z=1
1942.90939 z=1
2104.96199 z=1
2267.01961 z=?
2429.09502 z=?
m/z
LFNVTSTLR
m/z 1296.065 (+2)
Δ = 3.0 ppm
Melanoma B14
366.13940
1050.59418
1217.65044
1133.63063
1415.72456
1296.64197
1456.75263
1618.80443
1577.77997
1780.85796
1739.83192
1888.91750
2104.96777
2008.59494
2191.95652
2362.26936
2604.27798
325.11275
233.16490
528.19182
627.34050
703.38048
790.44211
993.52035
m/z

Figure S9

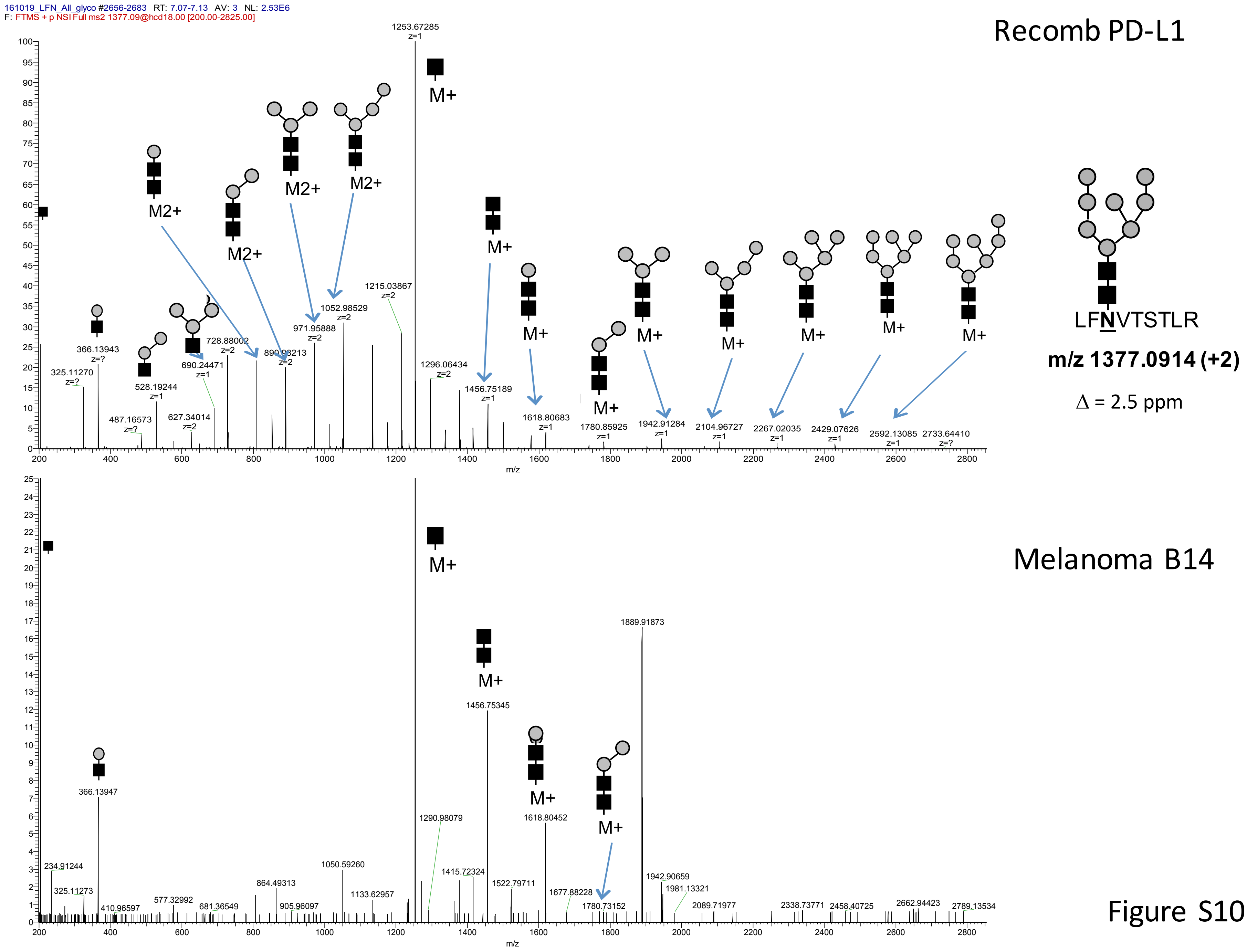

161019_LFN_All_glyco #2656-2683 RT: 7.07-7.13 AV: 3 NL: 2.53E6
F: FTMS + p NSI Full ms2 1377.09@hcd18.00 [200.00-2825.00]
Recomb PD-L1
1253.67285 z=1
M+
M2+
366.13943 z=?
325.11270 z=?
528.19244 z=1
487.16573 z=?
690.24471 z=1
627.34014 z=2
728.88002 z=2
890.93213 z=2
971.95888 z=2
1052.98529 z=2
1215.03867 z=2
1296.06434 z=2
1456.75189 z=1
1618.80683 z=1
1780.85925 z=1
1942.91284 z=1
2104.96727 z=1
2267.02035 z=1
2429.07626 z=1
2592.13085 z=1
2733.64410 z=?
m/z
LFNVTSTLR
m/z 1377.0914 (+2)
Δ = 2.5 ppm
Melanoma B14
366.13947
234.91244
325.11273
410.96597
577.32992
681.36549
864.49313
905.96097
1050.59260
1133.62957
1290.98079
1415.72324
1456.75345
1522.79711
1618.80452
1677.88228
1780.73152
1889.91873
1942.90659
1981.13321
2089.71977
2338.73771
2458.40725
2662.94423
2789.13534

Figure S10

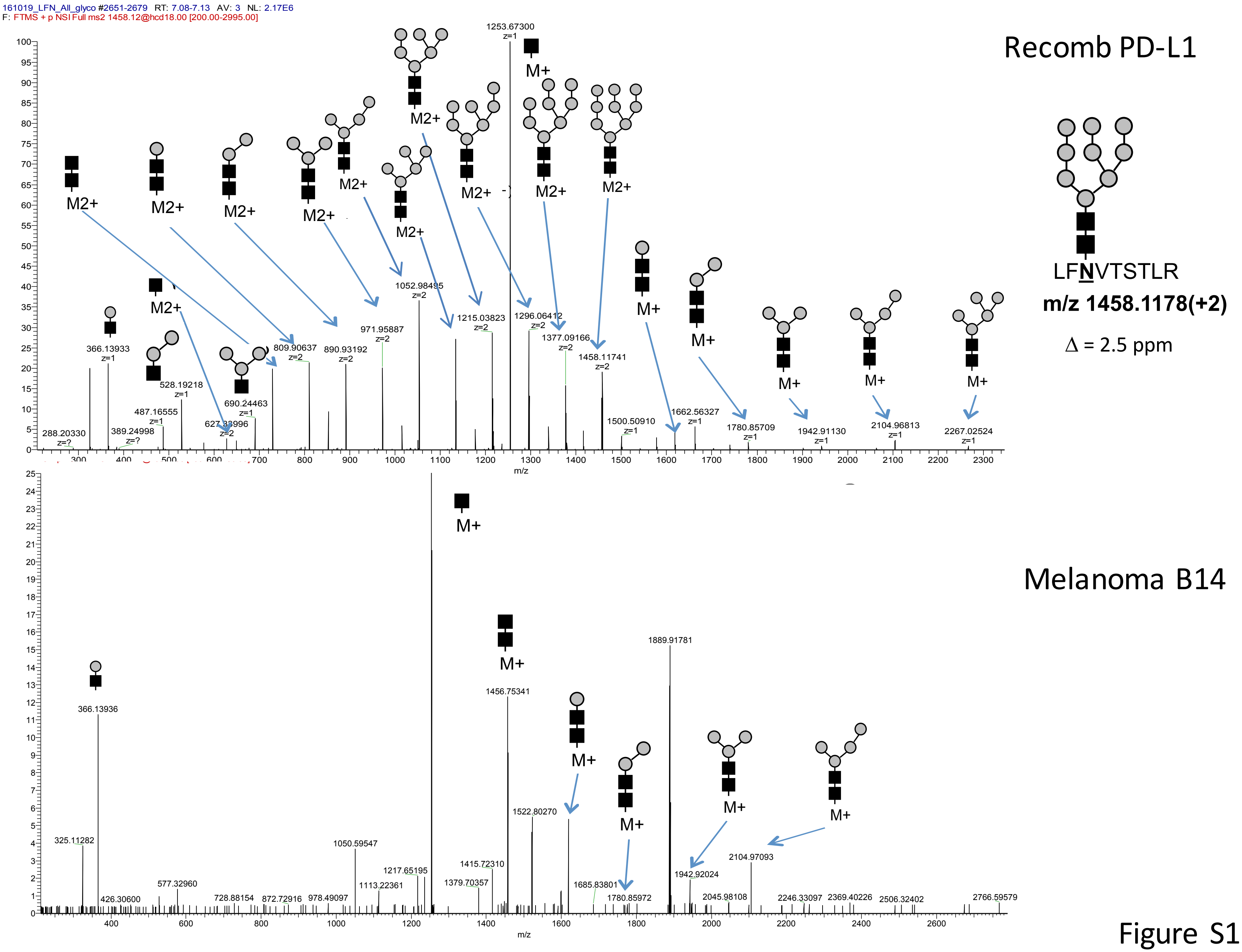
161019_LFN_All_glyco #2651-2679 RT: 7.08-7.13 AV: 3 NL: 2.17E6
F: FTMS + p NSI Full ms2 1458.12@hcd18.00 [200.00-2995.00]
Recomb PD-L1
LFNVTSTLR
m/z 1458.1178(+2)
Δ = 2.5 ppm
M2+
M+
1253.67300
z=1
1052.98495
z=2
1215.03823
z=2
1296.06412
z=2
971.95887
z=2
1377.09166
z=2
1458.11741
z=2
809.90637
z=2
890.93192
z=2
366.13933
z=1
528.19218
z=1
487.16555
z=1
690.24463
z=1
288.20330
z=?
389.24998
z=?
1500.50910
z=1
1662.56327
z=1
1780.85709
z=1
1942.91130
z=1
2104.96813
z=1
2267.02524
z=1
m/z
Melanoma B14
366.13936
325.11282
426.30600
577.32960
728.88154
872.72916
978.49097
1050.59547
1113.22361
1217.65195
1379.70357
1415.72310
1456.75341
1522.80270
1685.83801
1780.85972
1889.91781
1942.92024
2045.98108
2104.97093
2246.33097
2369.40226
2506.32402
2766.59579
m/z

Figure S11
