# Supplementary material for: Quantitative Mass Spectrometry Analysis of PD-L1 Protein Expression, N-glycosylation and Expression Stoichiometry with PD-1 and PD-L2 in Human Melanoma
Source: Mol Cell Proteomics. 2017 May 25;16(10):1705–17. doi: 10.1074/mcp.RA117.000037 (PMC5629259; doi:10.1074/mcp.RA117.000037)
Supplement: Supplemental Data [file supp_RA117.000037_4581_1_supp_1260_pq7ppf.pdf]

Morales-Betanzos et al. Supplemental Dataset 1

H&E and IHC micrographs for melanoma sections

Each page shows H&E (top) an IHC (bottom) stained for PD-L1 (red) and SOX10 (brown) at 20x magnification.

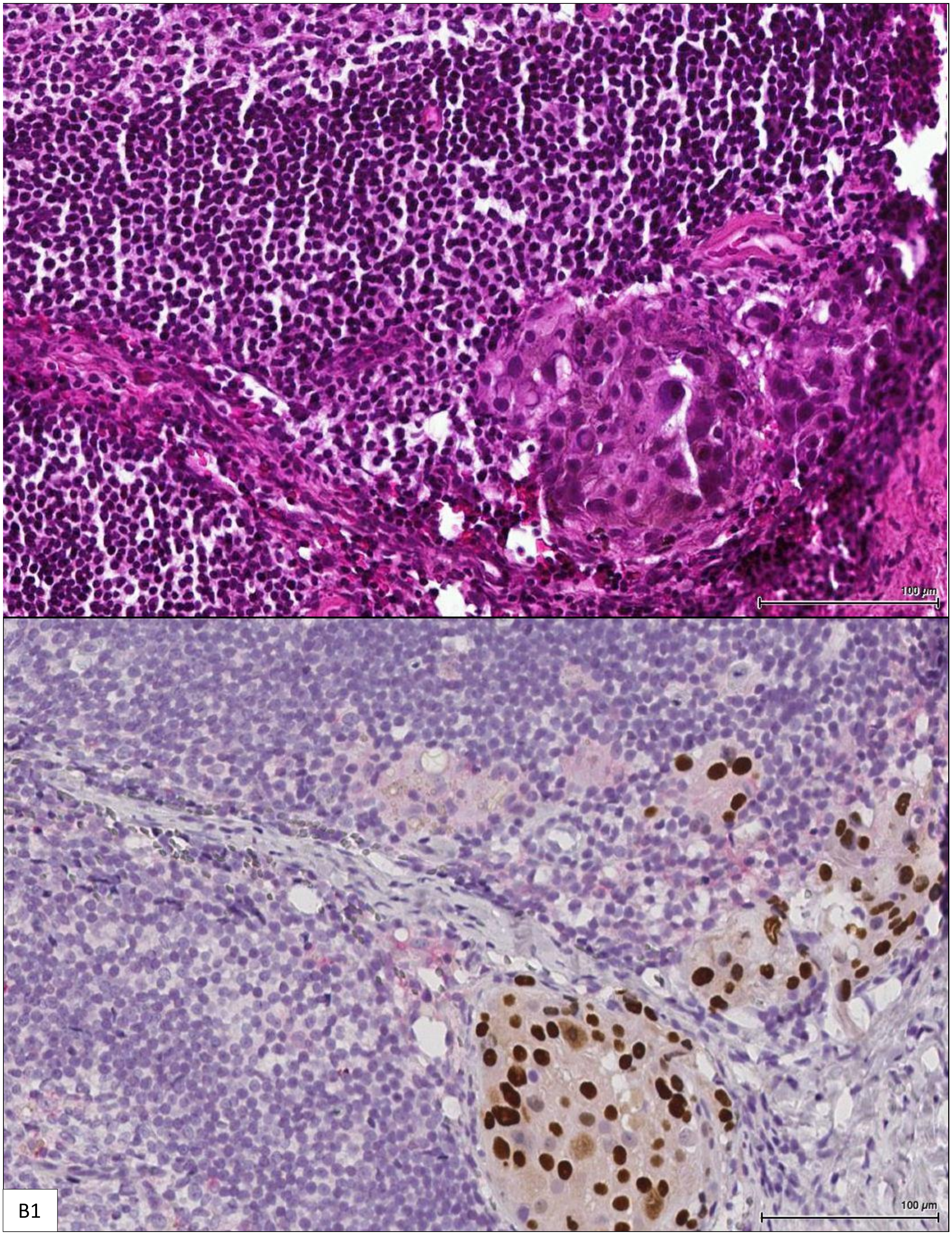
100 µm
100 µm
B1

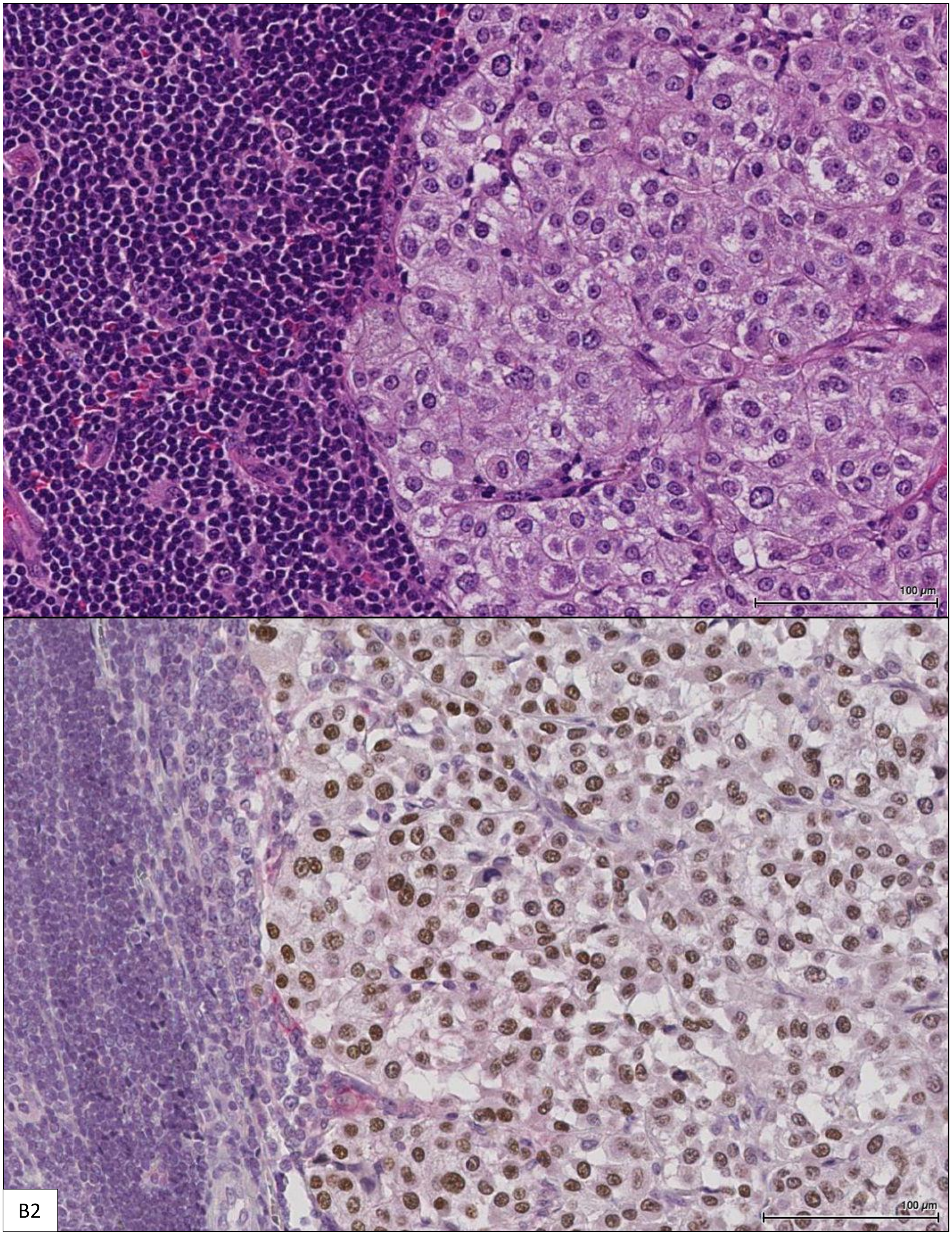

B2

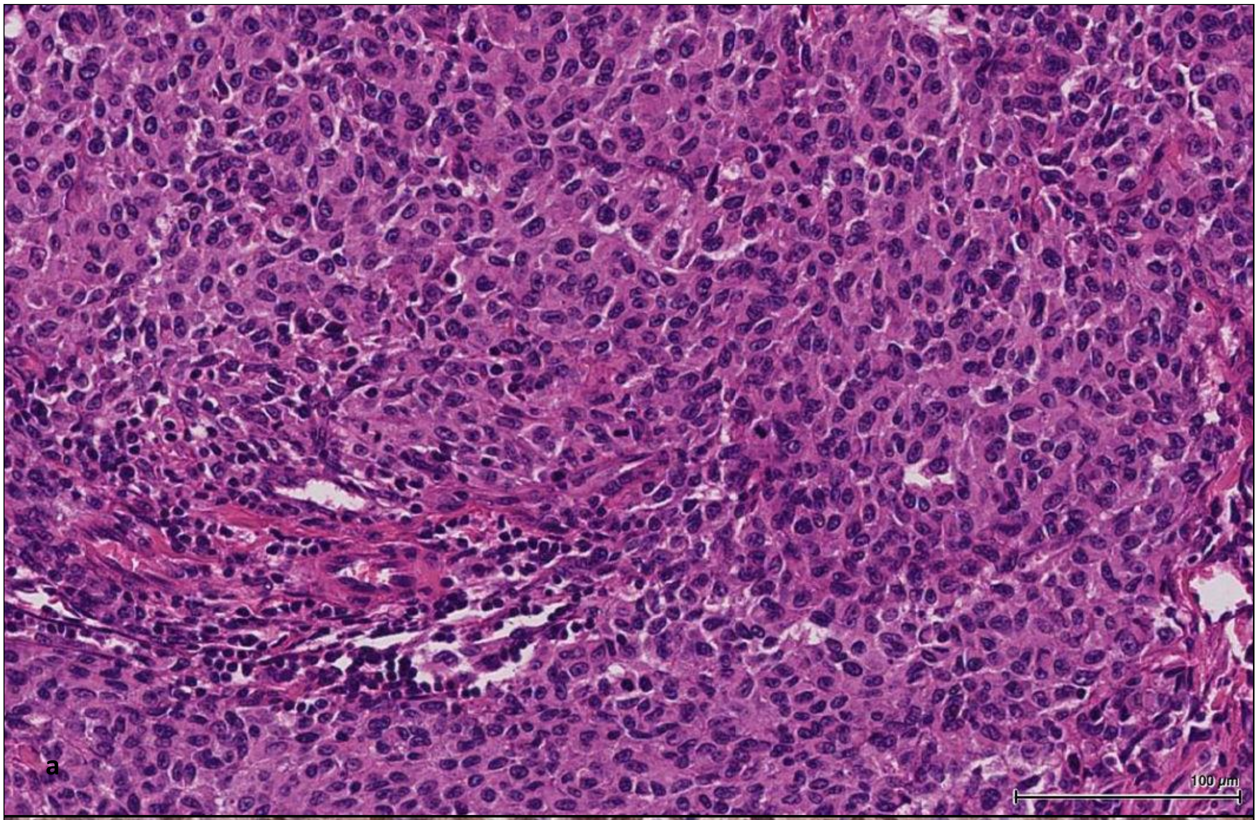
a
100 μm

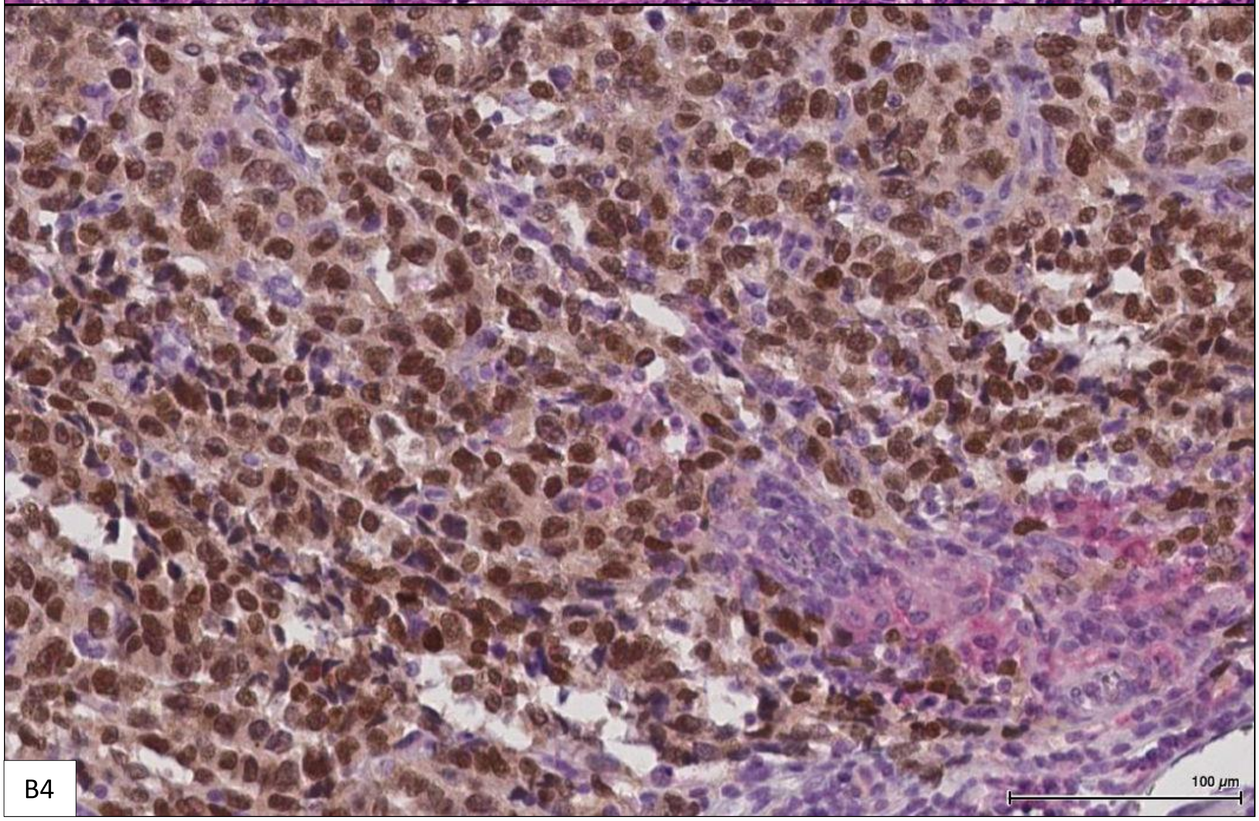
B4
100 μm

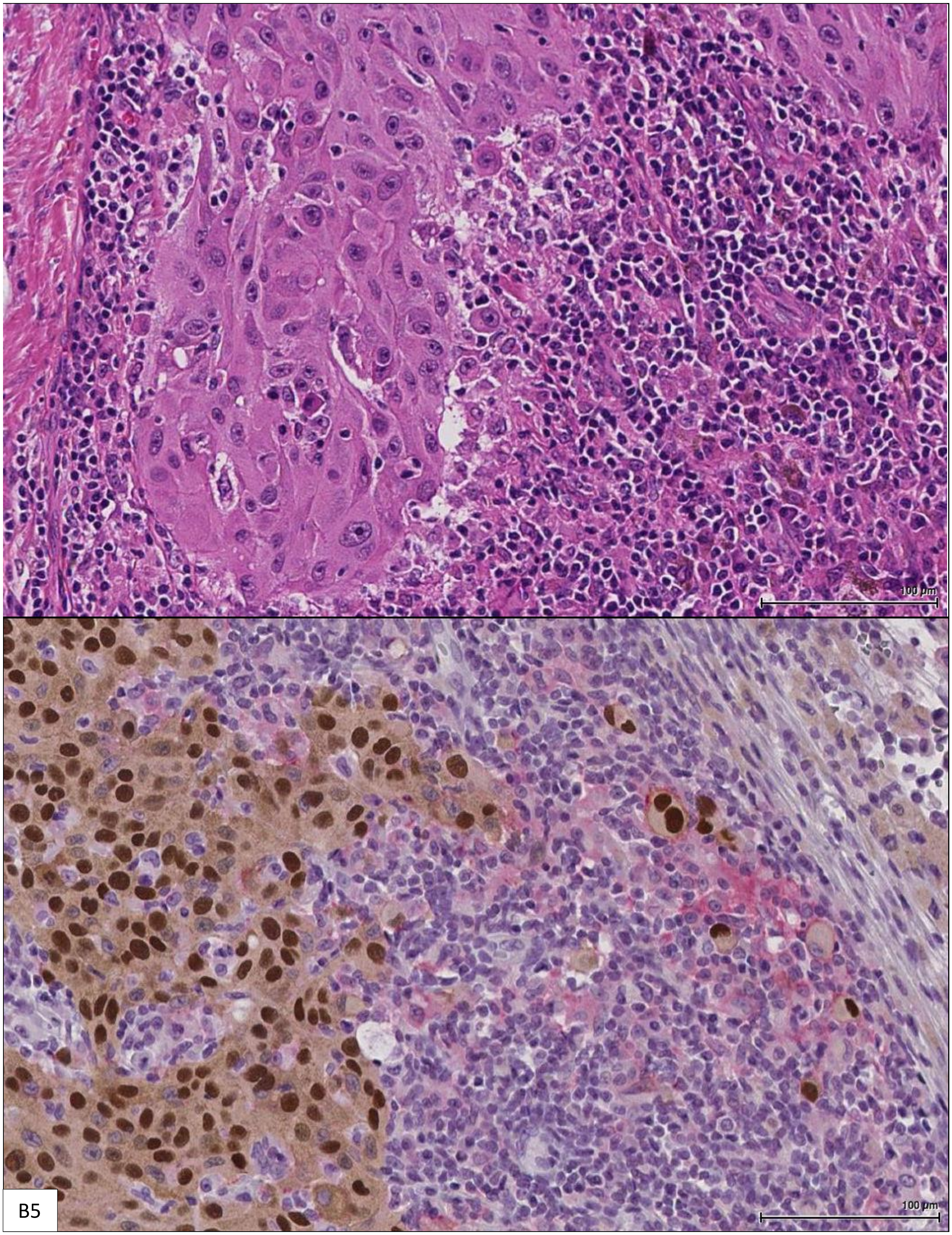
100 µm
100 µm
B5

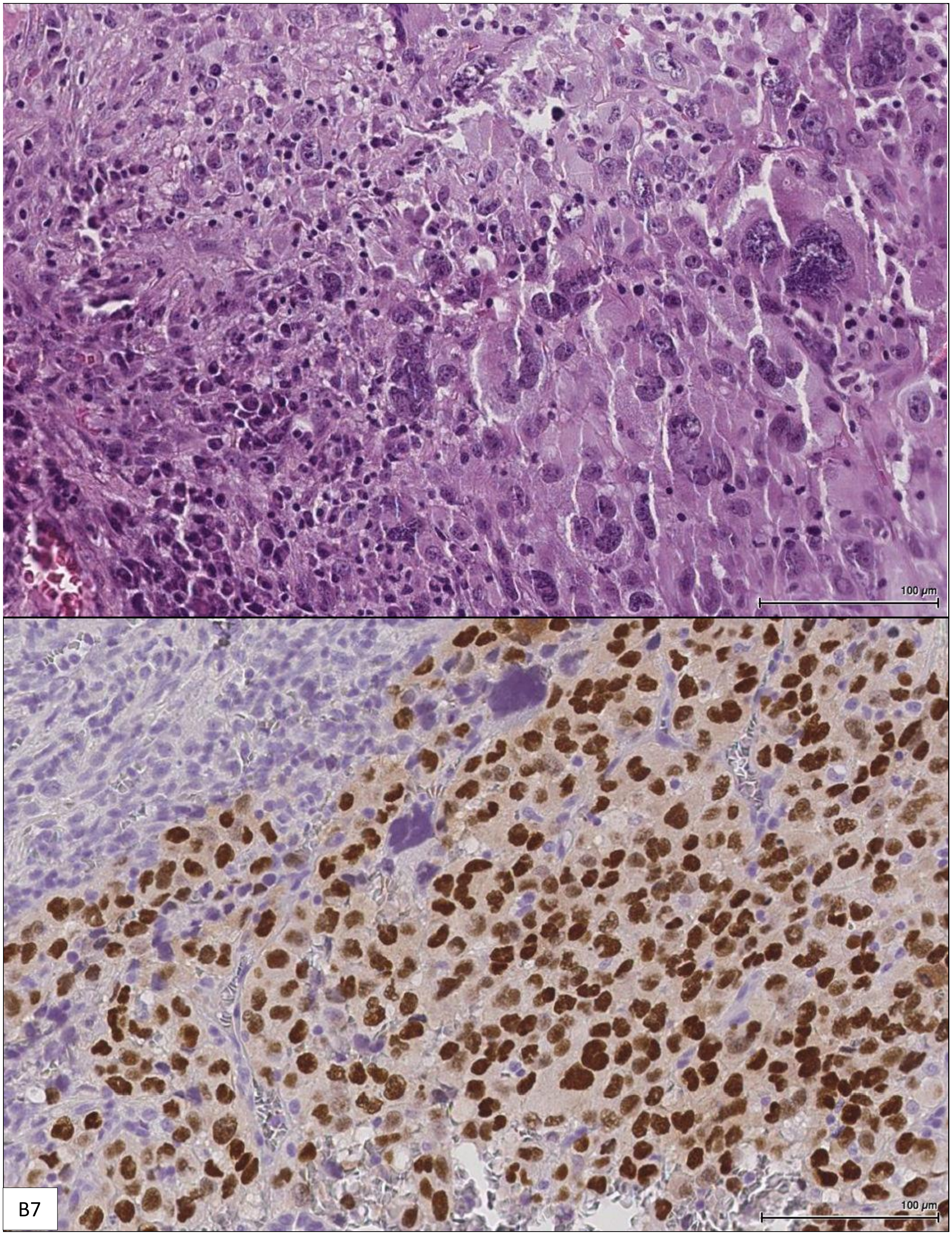
100 µm
B7
100 µm

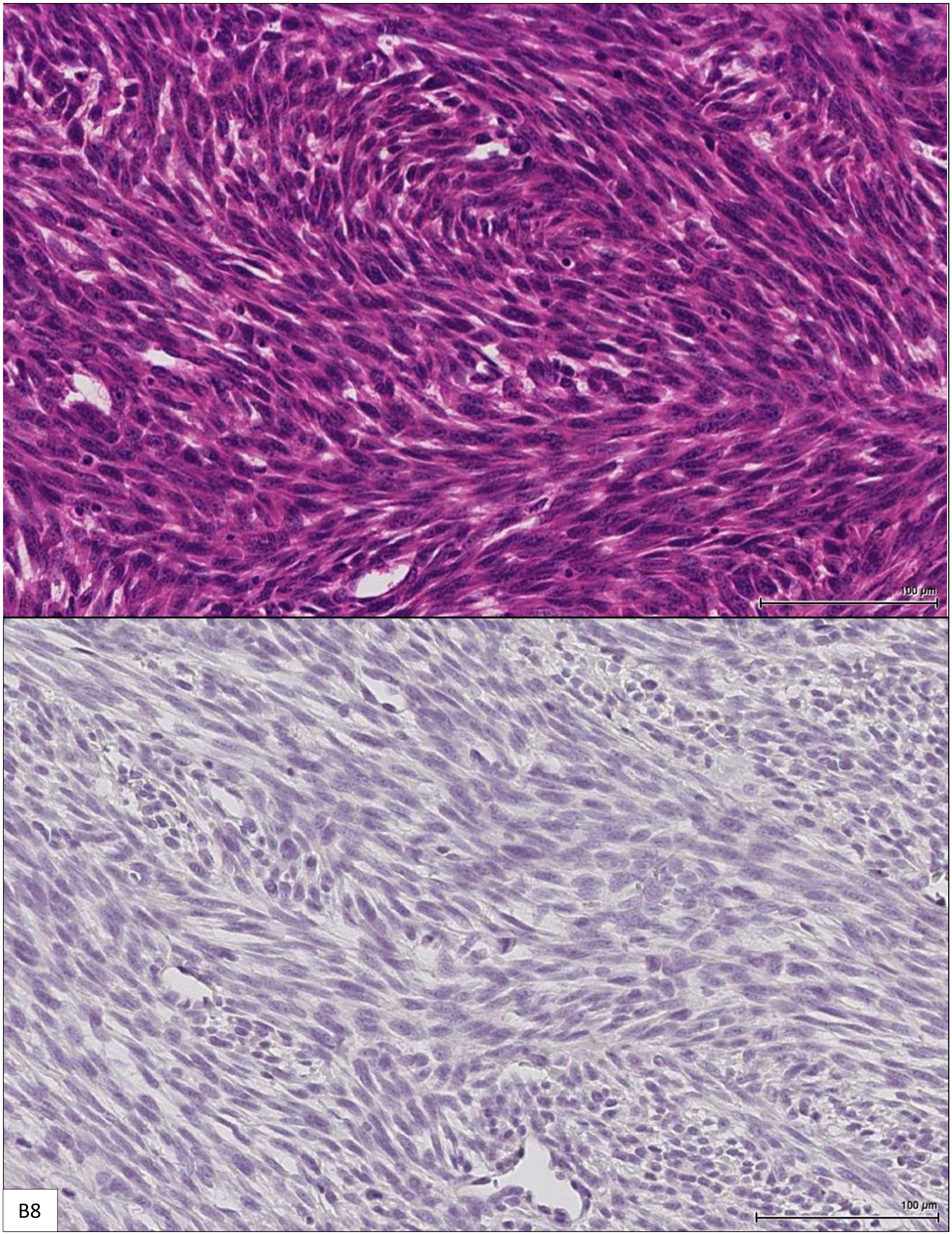
100 µm
100 µm
B8

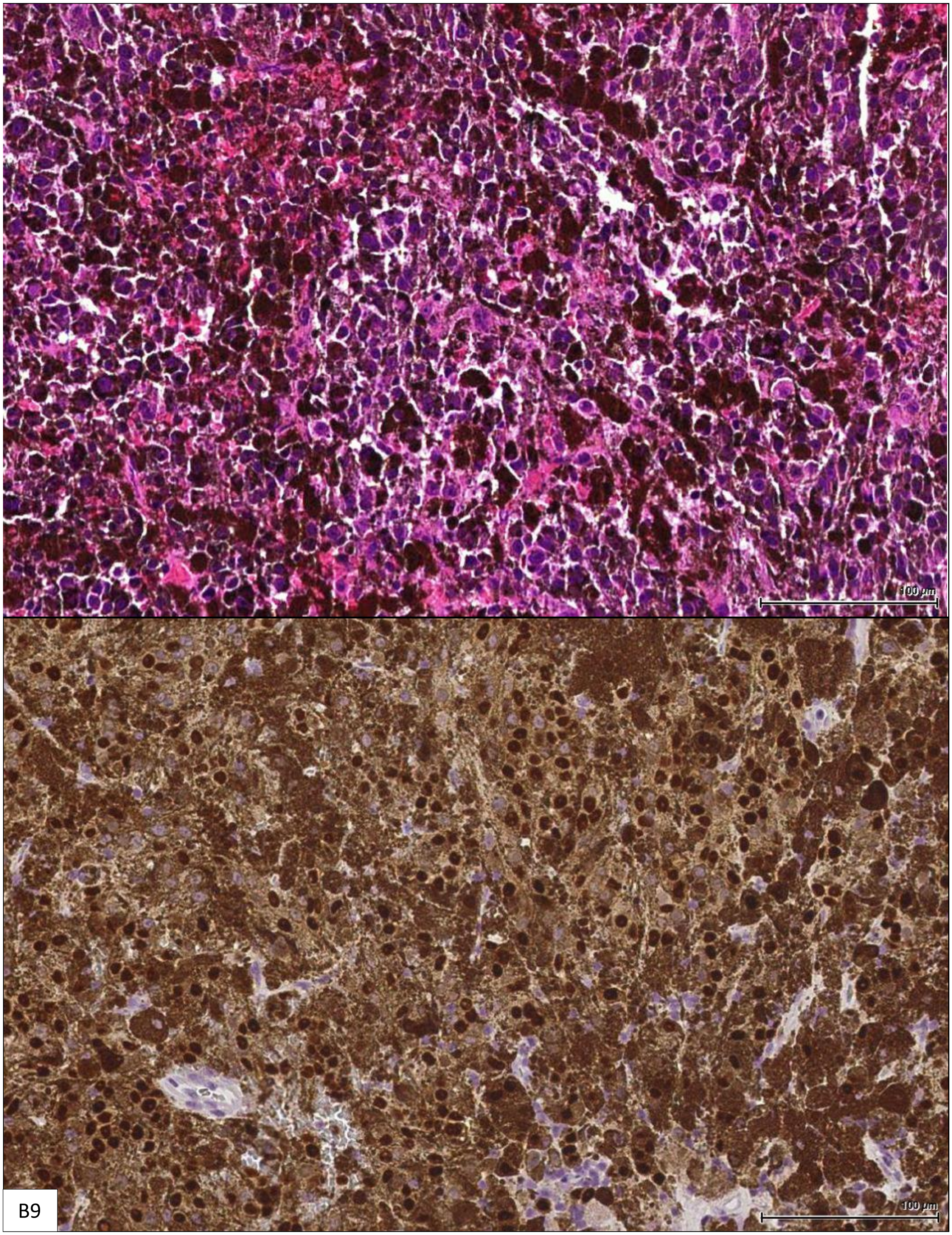

B9

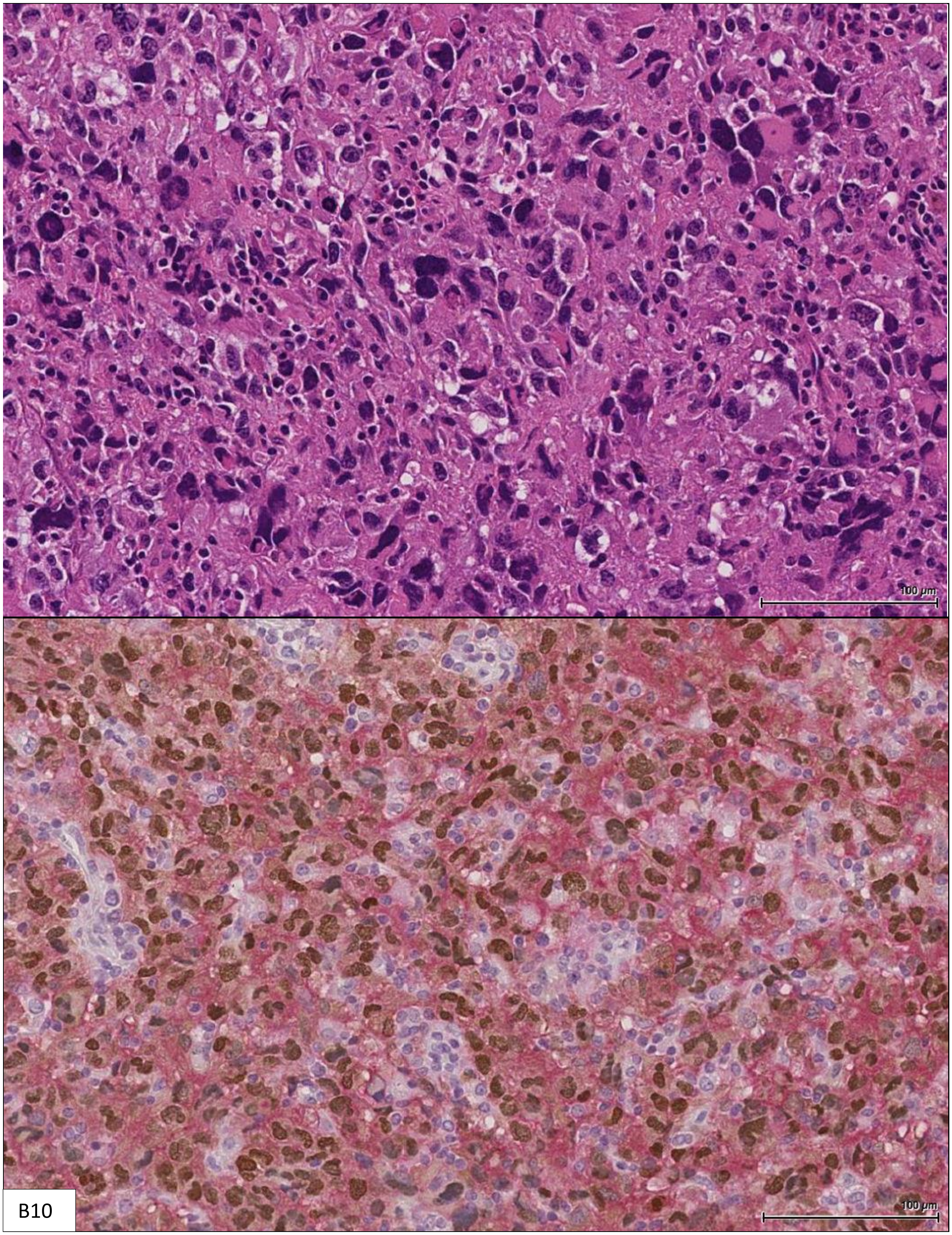
100 µm
100 µm
B10

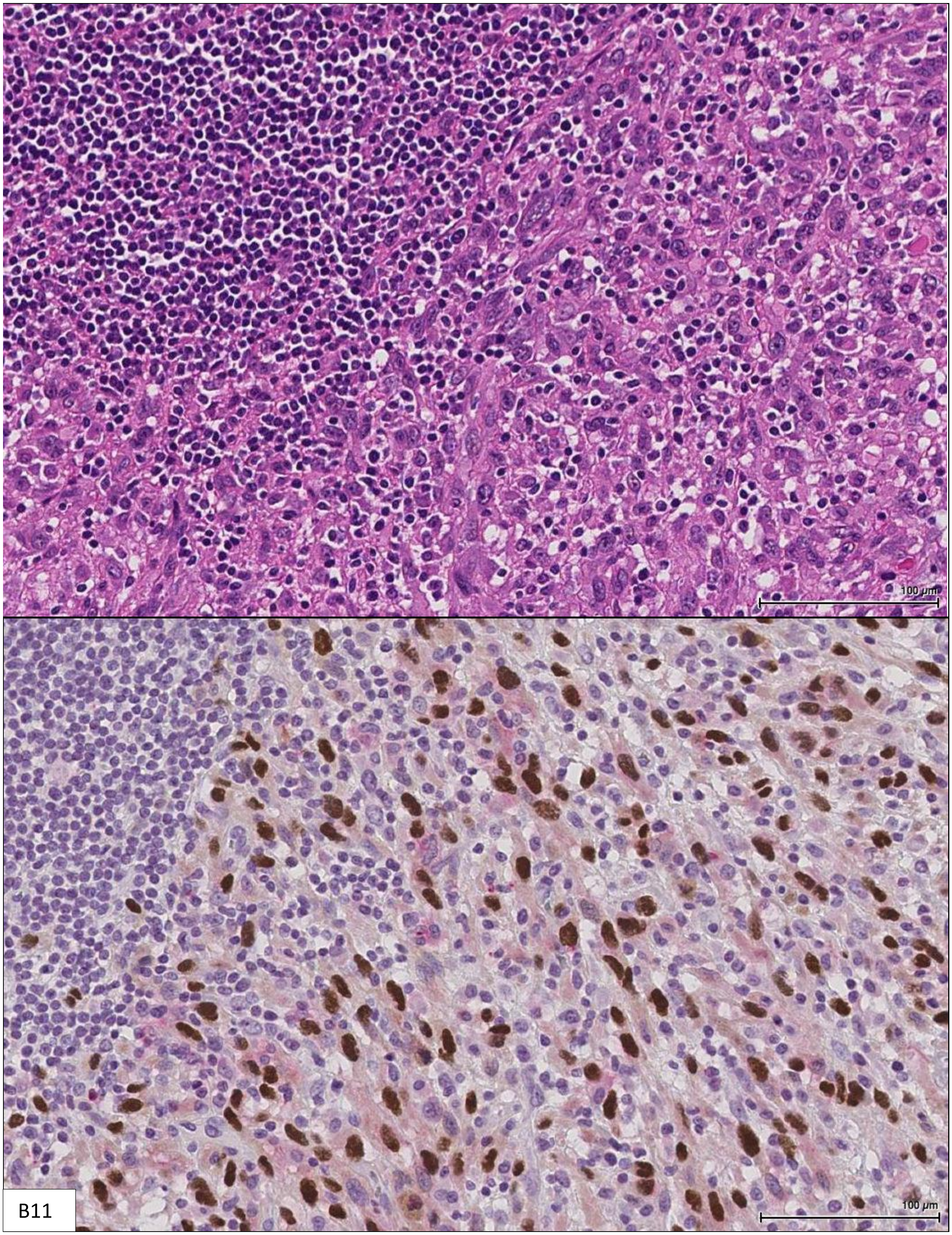

B11

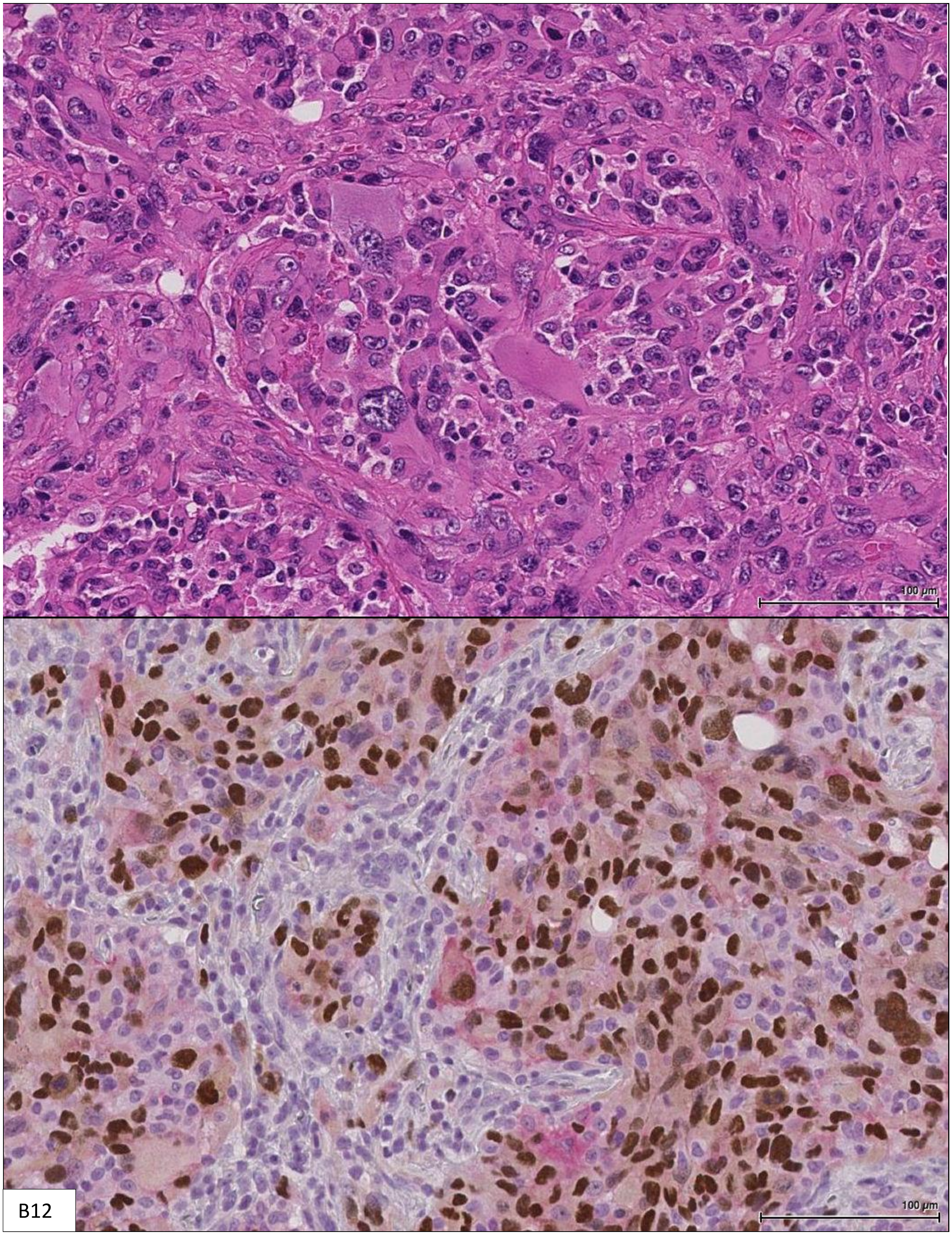

B12

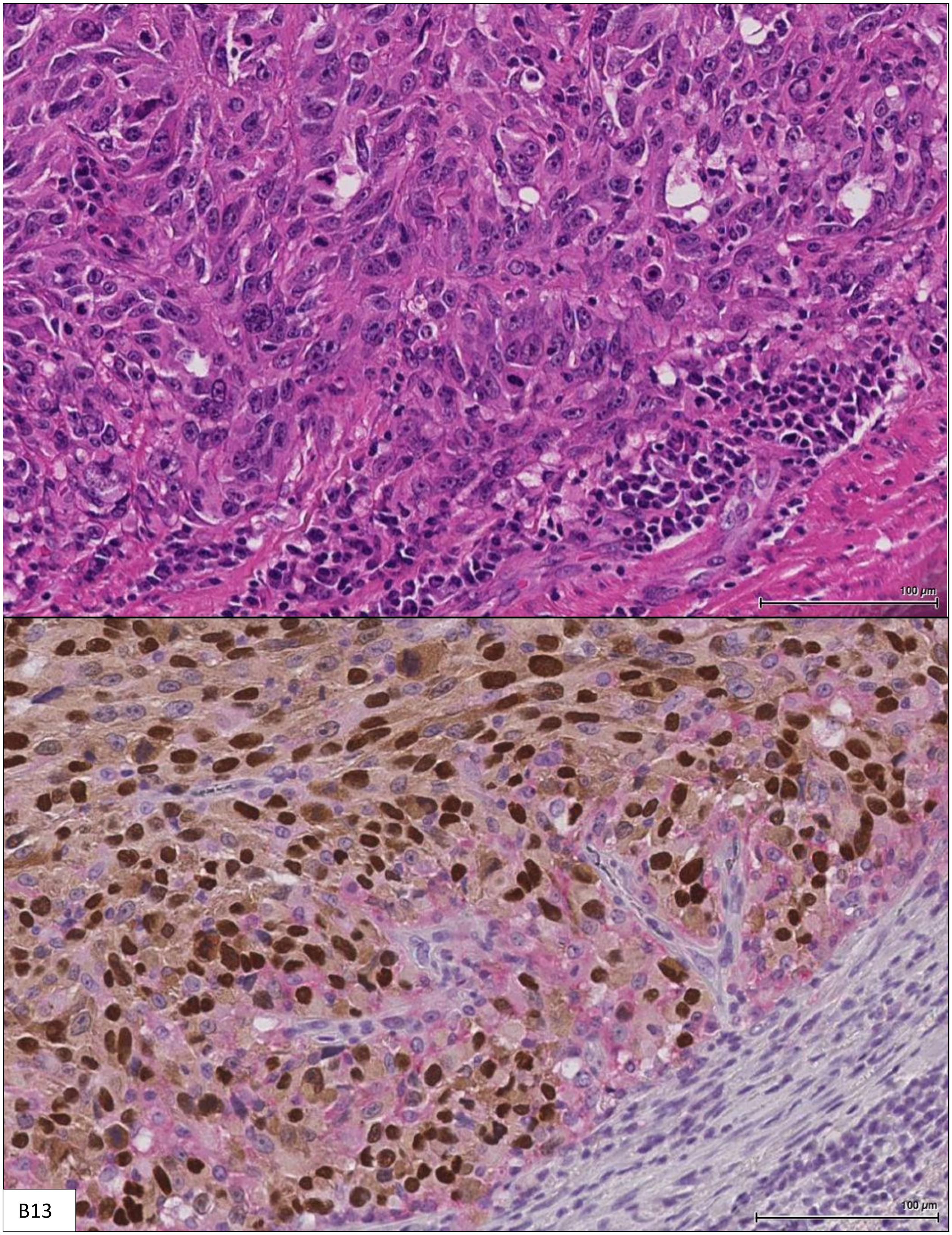

B13

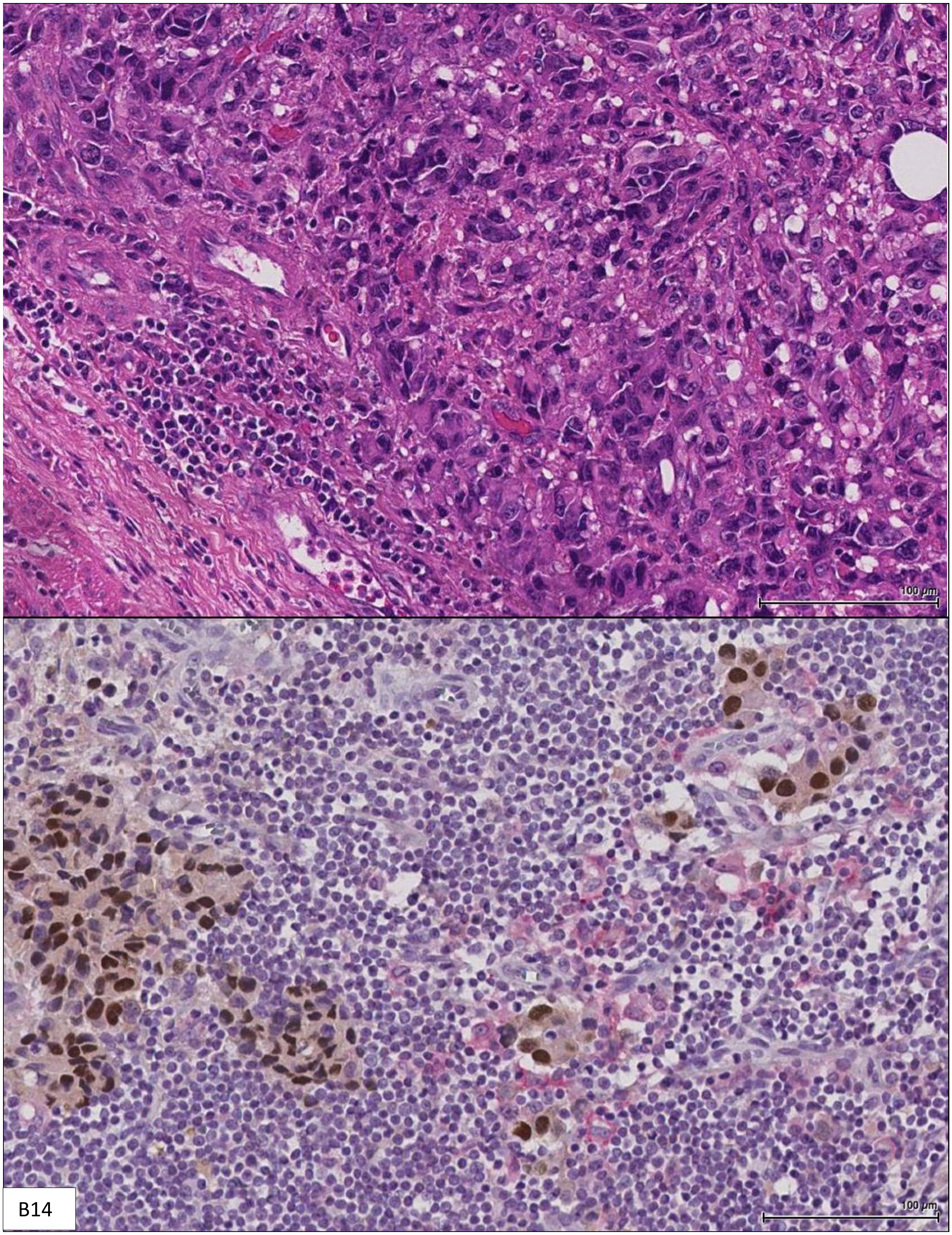

B14

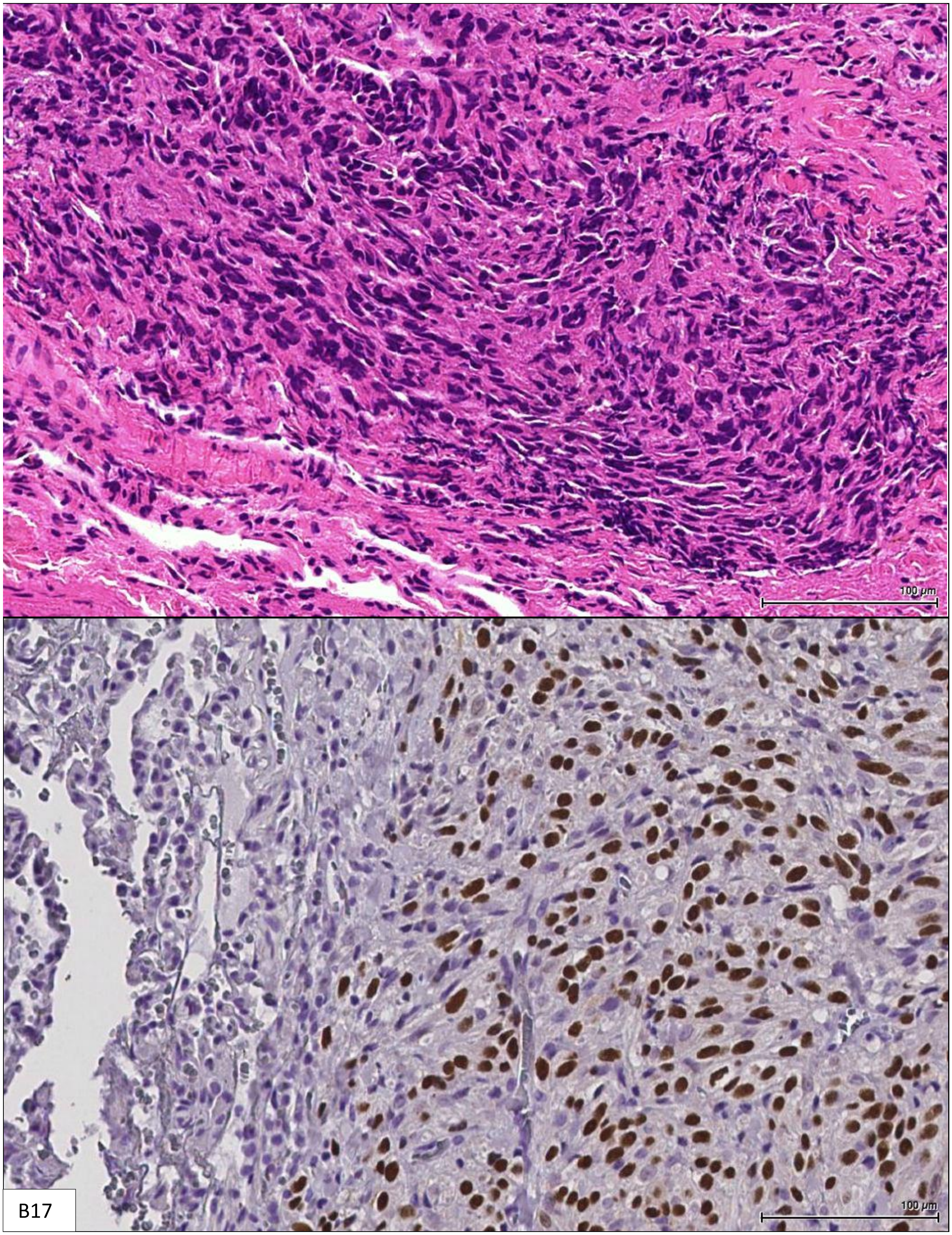

B17

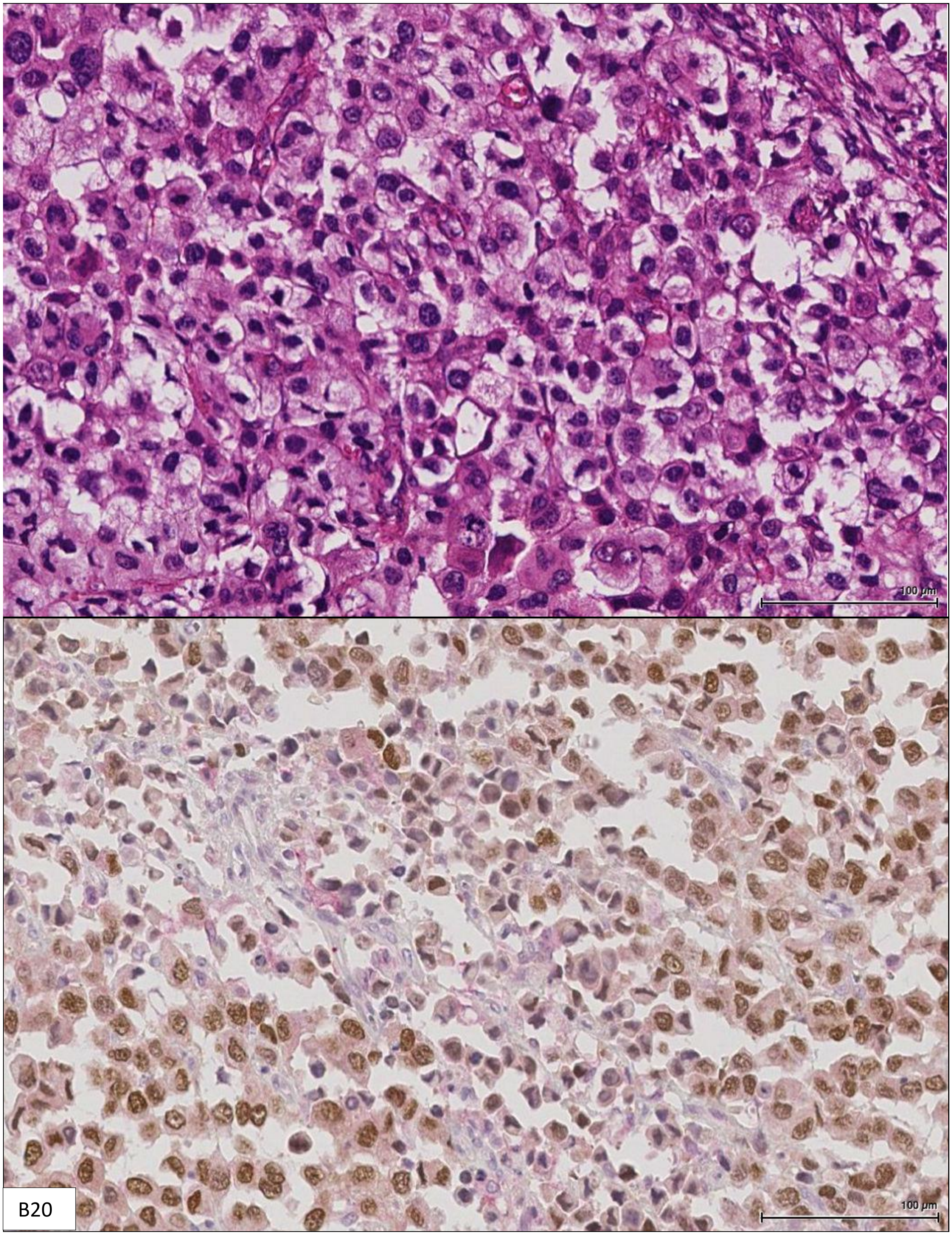
100 µm
100 µm
B20

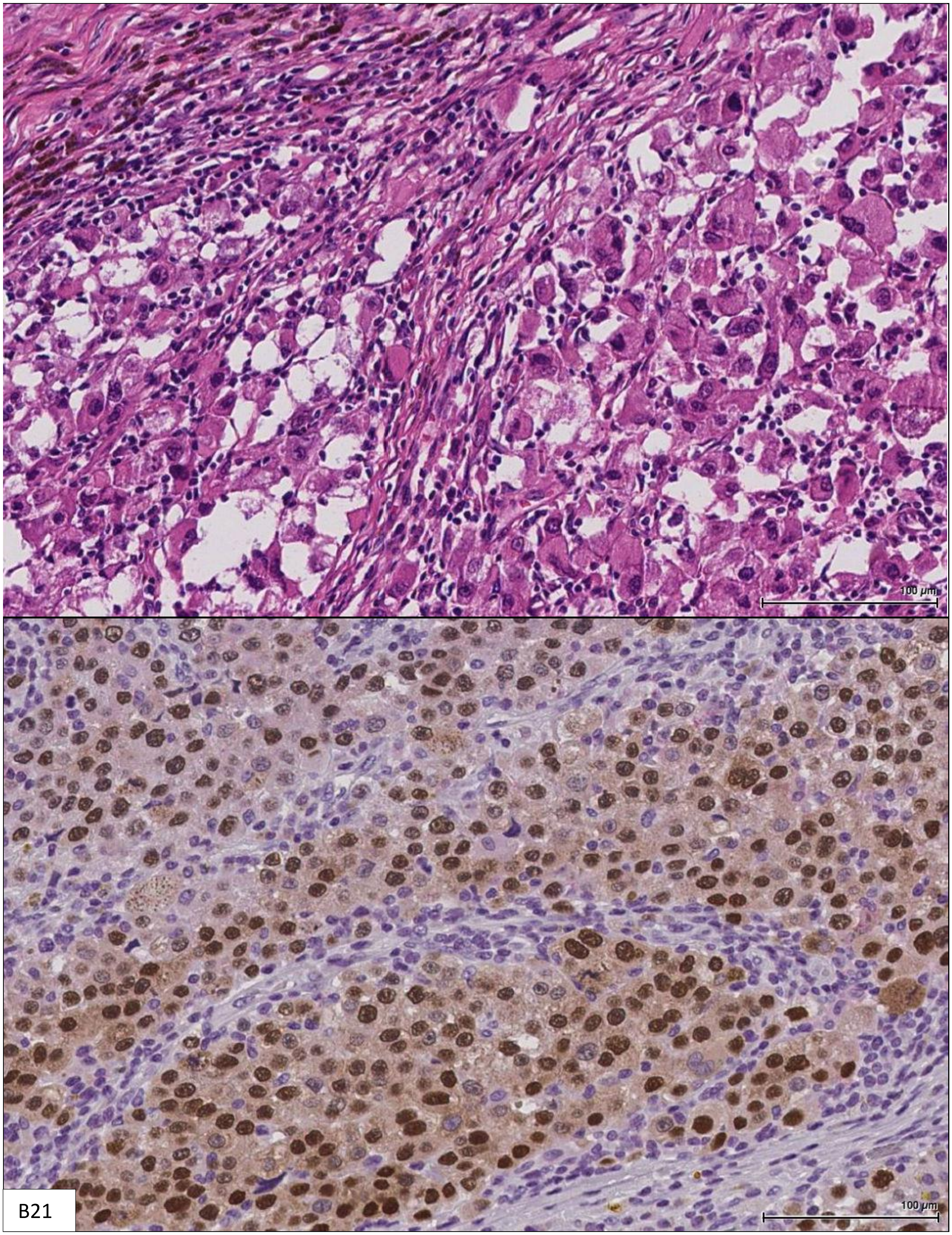
100 µm
100 µm
B21

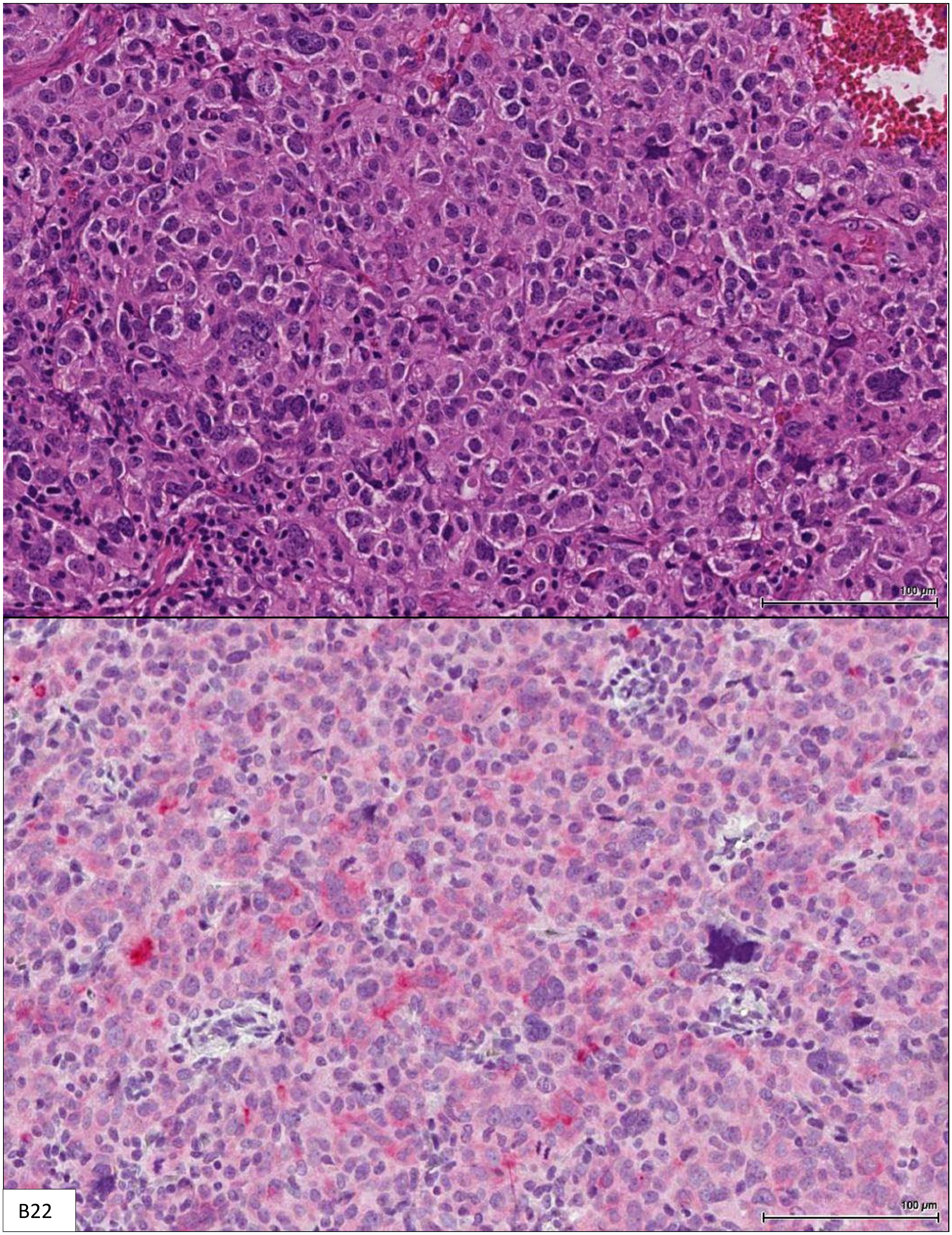

B22

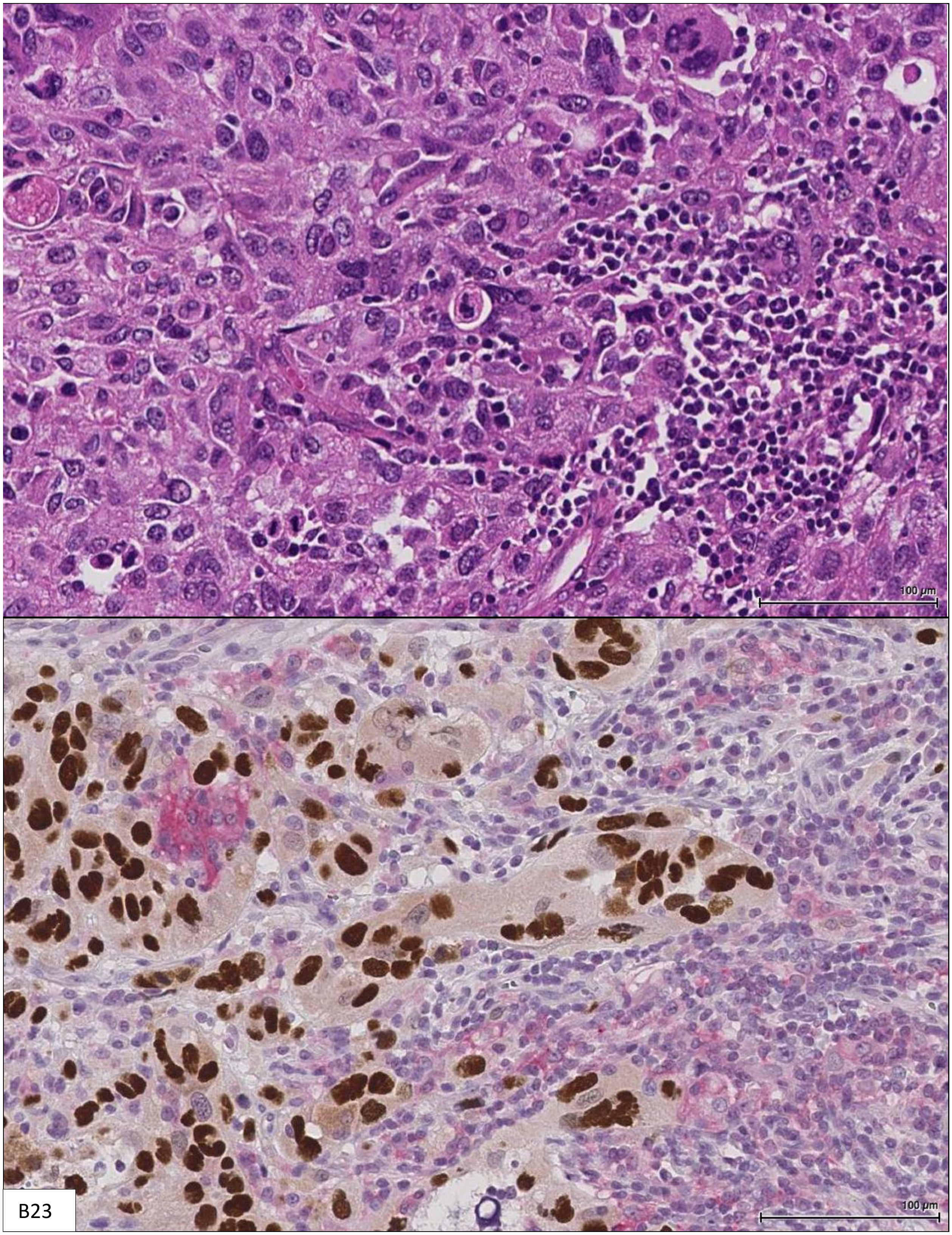
100 µm
100 µm
B23

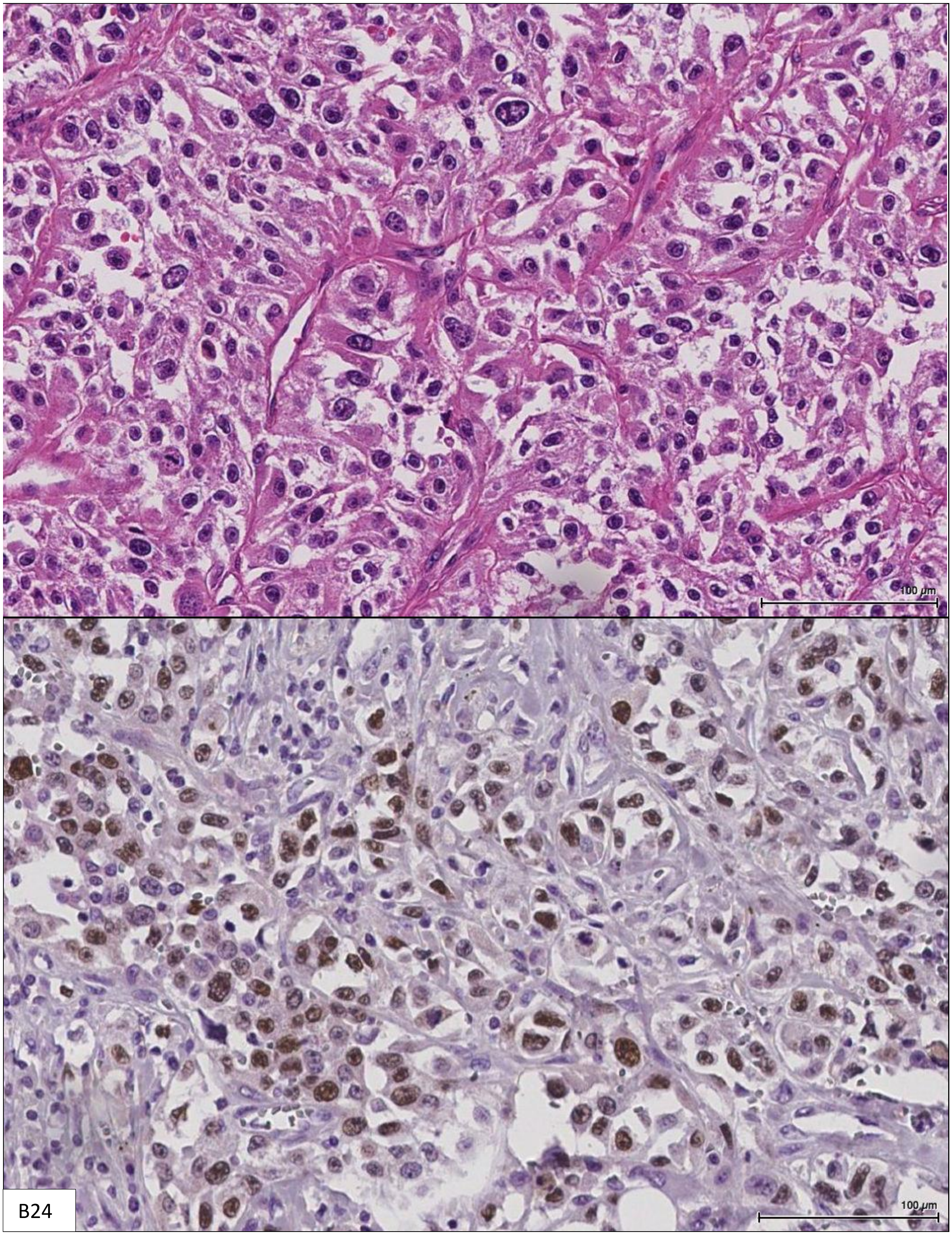

B24

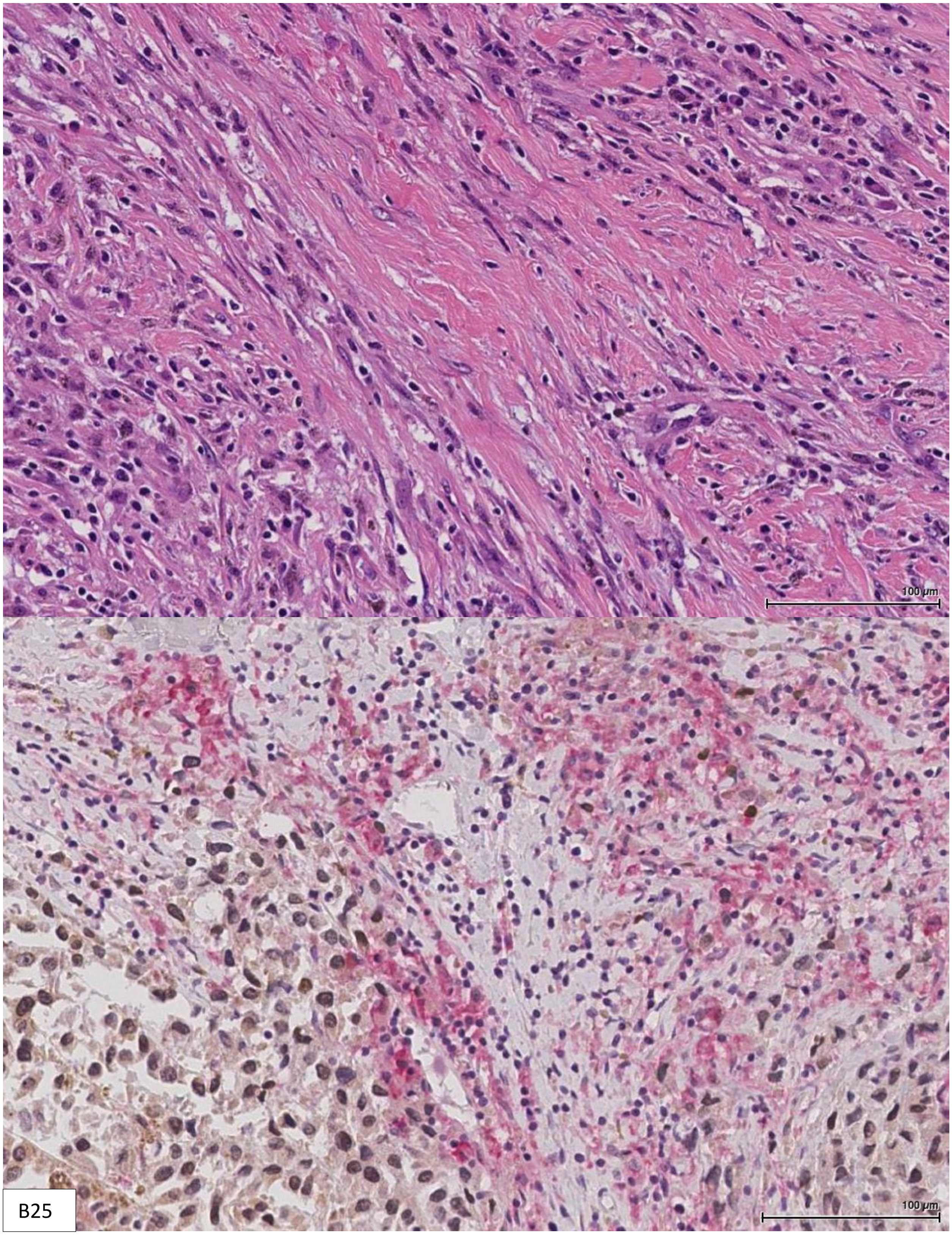

B25

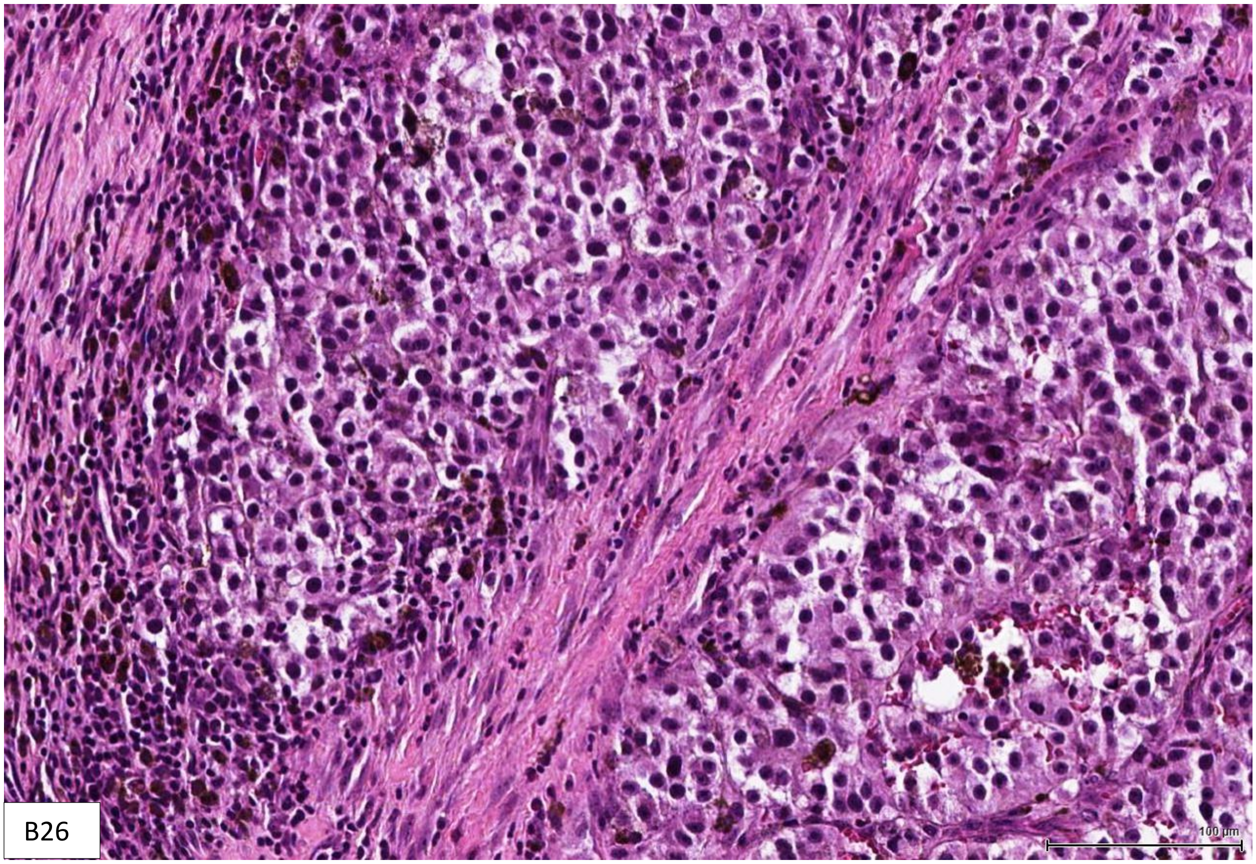
B26
100 µm

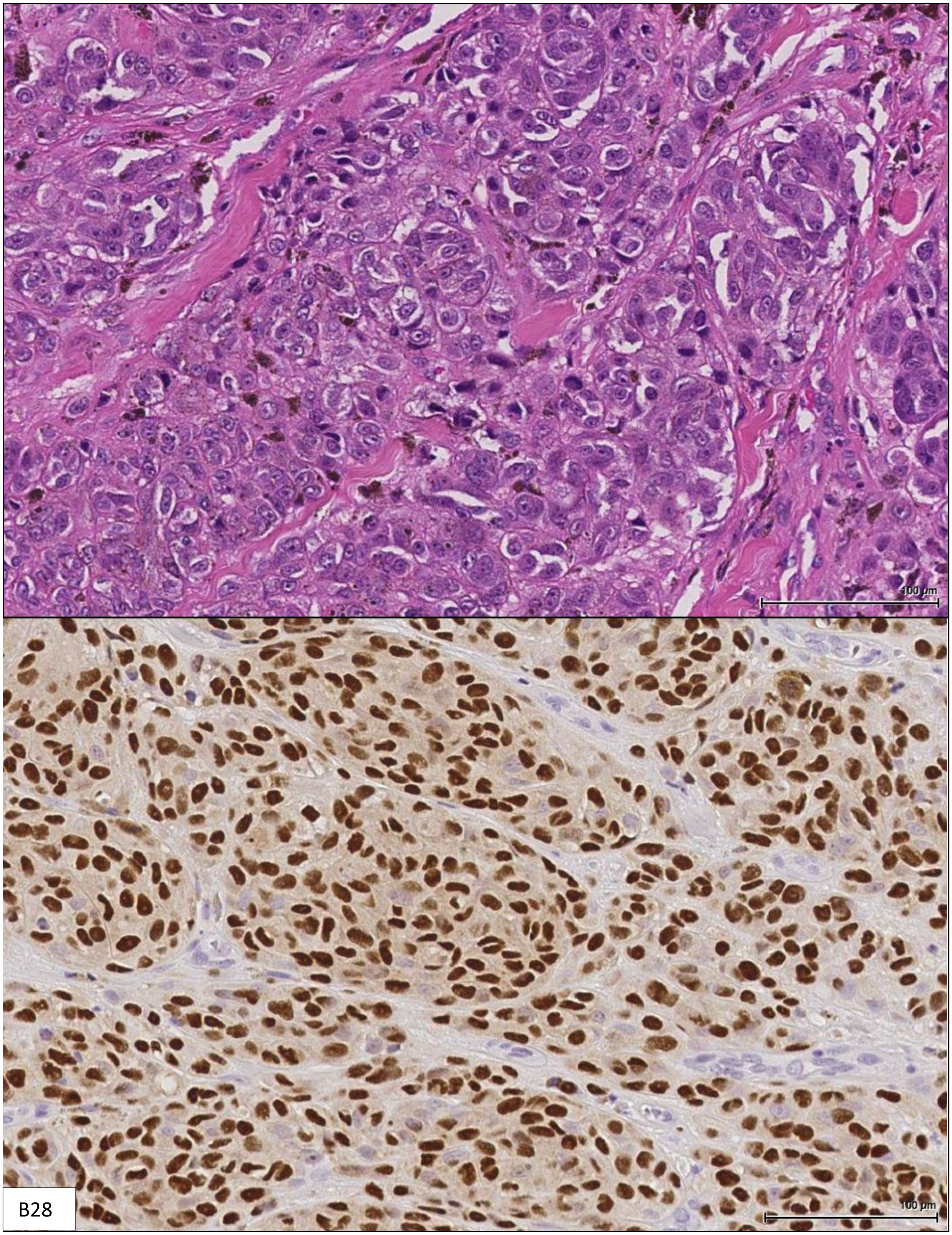

B28

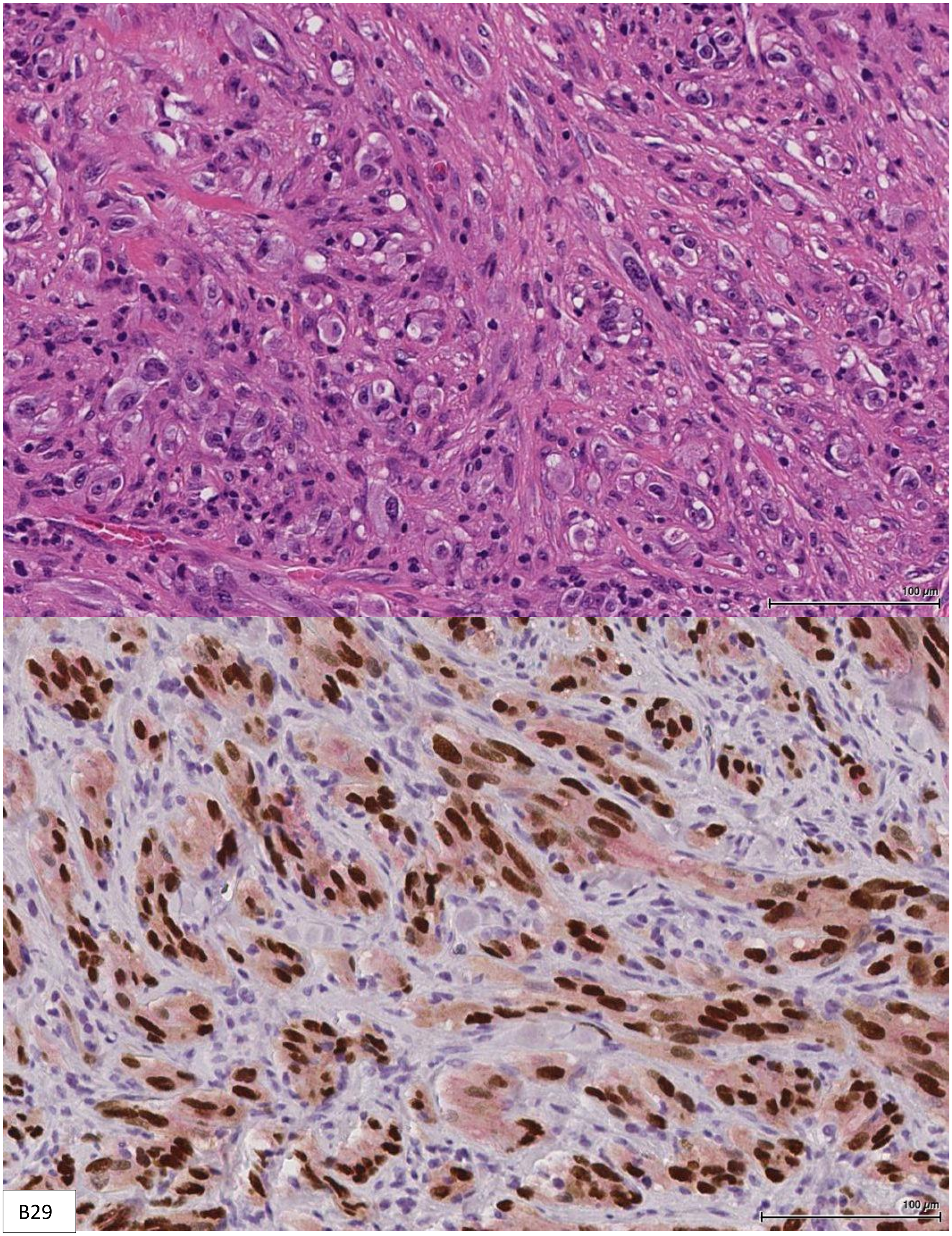

B29
